# Post-synthetic modulation of the charge distribution in a metal–organic framework for optimal binding of carbon dioxide and sulfur dioxide†
†Electronic supplementary information (ESI) available: Synthetic details, structural data, figures, PXRD patterns, TGA, IR spectroscopic and sorption analyses. This material is available in the online version of paper. Correspondence of requests for materials should be addressed to S. Y. and M. S. CCDC 1580818–1580823, 1580825–1580829, 1580831, 1580832, 1580833, 1580834. For ESI and crystallographic data in CIF or other electronic format see DOI: 10.1039/c8sc01959b


**DOI:** 10.1039/c8sc01959b

**Published:** 2018-10-31

**Authors:** Lei Li, Ivan da Silva, Daniil I. Kolokolov, Xue Han, Jiangnan Li, Gemma Smith, Yongqiang Cheng, Luke L. Daemen, Christopher G. Morris, Harry G. W. Godfrey, Nicholas M. Jacques, Xinran Zhang, Pascal Manuel, Mark D. Frogley, Claire A. Murray, Anibal J. Ramirez-Cuesta, Gianfelice Cinque, Chiu C. Tang, Alexander G. Stepanov, Sihai Yang, Martin Schroder

**Affiliations:** a School of Chemistry , University of Manchester , Oxford Road , Manchester , M13 9PL , UK . Email: Sihai.Yang@manchester.ac.uk ; Email: M.Schroder@manchester.ac.uk; b Lehn Institute of Functional Materials , School of Chemistry , Sun Yat-Sen University , Guangzhou , 510275 , China; c ISIS Neutron Facility , STFC Rutherford Appleton Laboratory , Chilton , Oxfordshire OX11 0QX , UK; d Boreskov Institute of Catalysis , Siberian Branch of Russian Academy of Sciences , Prospekt Akademika Lavrentieva 5 , Novosibirsk , 630090 , Russia; e Novosibirsk State University , Novosibirsk 630090 , Russia; f The Chemical and Engineering Materials Division (CEMD) , Neutron Sciences Directorate , Oak Ridge National Laboratory , Oak Ridge , TN 37831 , USA; g Diamond Light Source , Harwell Science Campus , Oxfordshire OX11 0DE , UK

## Abstract

Modulation of pore environment is an effective strategy to optimize guest binding in porous materials. We report the post-synthetic modification of the charge distribution in a charged metal–organic framework, MFM-305-CH_3_, [Al(OH)(L)]Cl, [(H_2_L)Cl = 3,5-dicarboxy-1-methylpyridinium chloride] and its effect on guest binding. MFM-305-CH_3_ shows a distribution of cationic (methylpyridinium) and anionic (chloride) centers and can be modified to release free pyridyl N-centres by thermal demethylation of the 1-methylpyridinium moiety to give the neutral isostructural MFM-305. This leads simultaneously to enhanced adsorption capacities and selectivities (two parameters that often change in opposite directions) for CO_2_ and SO_2_ in MFM-305. The host–guest binding has been comprehensively investigated by *in situ* synchrotron X-ray and neutron powder diffraction, inelastic neutron scattering, synchrotron infrared and ^2^H NMR spectroscopy and theoretical modelling to reveal the binding domains of CO_2_ and SO_2_ in these materials. CO_2_ and SO_2_ binding in MFM-305-CH_3_ is shown to occur *via* hydrogen bonding to the methyl and aromatic-CH groups, with a long range interaction to chloride for CO_2_. In MFM-305 the hydroxyl, pyridyl and aromatic C–H groups bind CO_2_ and SO_2_ more effectively *via* hydrogen bonds and dipole interactions. Post-synthetic modification *via* dealkylation of the as-synthesised metal–organic framework is a powerful route to the synthesis of materials incorporating active polar groups that cannot be prepared directly.

## Abstract 2

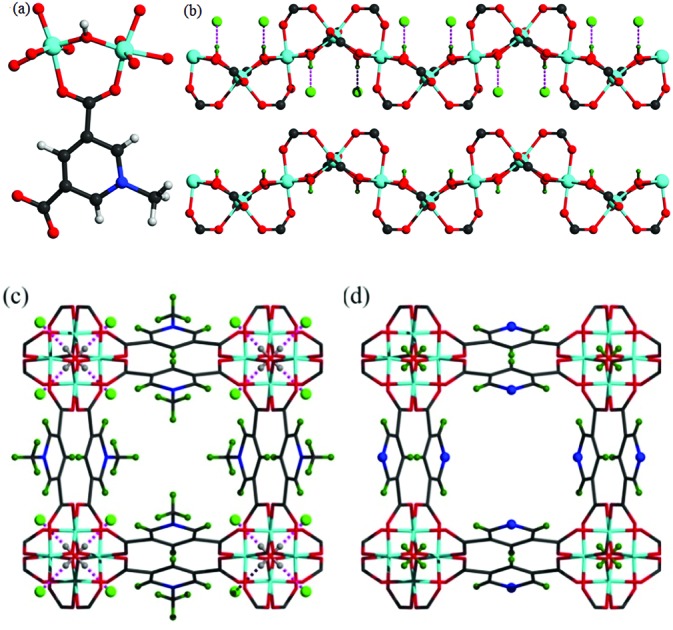
Modulation of pore environment is an effective strategy to optimize guest binding in porous materials.

## Introduction

The release of CO_2_ and SO_2_ into the atmosphere from combustion of fossil fuels causes significant environmental problems and health risks.^1^,^2^ The wholesale replacement of the carbon-based energy supply is highly challenging, not least because of the existing infrastructure.^3^ It is therefore vital to mitigate the emissions of these acidic gases post combustion. At present, several technologies are used to capture CO_2_ and SO_2_, such as amine scrubbing, absorption in organic solvents, ionic liquids and limestone slurry.^4^–^7^ However, the considerable costs and the substantial energy input required for system regeneration significantly limit their long-term application. Powerful drivers therefore exist to develop new systems showing high adsorption capacity, selectivity, storage density and excellent reversibility to sequester these gases.

Metal–organic frameworks (MOFs) constructed from metal ions and clusters bridged by organic ligands are an emerging class of porous materials showing highly crystalline structures and great promise for gas adsorption and storage.^8^ MOFs can exhibit very high surface areas and, more importantly, have tunable pore environments with predictable pore size^9^ and can incorporate specific functional groups.^10^–^14^ MOFs have been studied extensively as sorbents for CO_2_ under post-combustion conditions.^11^,^13^–^22^ In contrast, adsorption of SO_2_ in MOFs has been rarely reported due to the limited stability of coordination compounds to highly reactive and corrosive SO_2_.^21^–^27^ Development of new stable porous materials with optimal SO_2_ and CO_2_ adsorption property thus remains significant challenge. Optimising the interactions between hosts and substrate molecules to enhance the storage capacity, density and selectivity is the key to overcoming these barriers. For this reason, visualisation of the host–guest interactions involved in the MOF–gas binding interactions is crucial for the design of new materials. Interrogation of adsorption mechanisms by *in situ* experiments as a function of gas loading can afford key insights into the preferred binding sites within pores and the interaction to the pore interior.^13^,^14^,^21^,^22^ Open metal sites^11^,^28^ and pendent functional groups (*e.g.*, amine, hydroxyl group and nitrogen-containing aromatic rings)^13^,^14^,^21^,^22^,^29^ have been found to act as specific sites for CO_2_ and SO_2_ binding.

Ionic liquids are composed of cations such as ammonium, pyridinium, phosphonium and imidazolium groups, and the solubility of CO_2_ and SO_2_ in these systems is often high owing to strong acid–base interaction.^6^,^30^–^32^ The incorporation of functionalized organic ligands within MOFs,^23^–^25^ and post synthetic modification can be used to control the number and types of functional groups within the pores.^15^–^17^ Cleavage of C–N bonds and hydrolysis of methyl viologen in alkaline solution have been observed.^18^,^19^ However, the dealkylation of pre-formed MOFs has not been reported previously, although adsorption of CO_2_ in two imidazolium–pyridinium cation-containing MOFs has been observed.^10^,^33^ We report the synthesis of a highly unusual charged material MFM-305-CH_3_, [Al(OH)(L)]Cl, [(H_2_L)Cl = 3,5-dicarboxy-1-methylpyridinium chloride] incorporating cationic (methylpyridinium) and anionic (chloride) components giving the material zwitterionic features. By heating MFM-305-CH_3_ at 180 °C, the 1-methylpyridiniumdicarboxylate ligand undergoes *in situ* demethylation to give the pyridine-based neutral complex MFM-305 showing the same overall framework topology. Demethylation coupled to loss of chloride anion exposes the bridging hydroxyl group in the MOF pore for hydrogen bonding to substrates, with MFM-305-CH_3_ and MFM-305 showing distinct charge distributions and pore environments decorated with different functional groups. This provides a unique platform to investigate the precise roles of Lewis acid, Lewis base, chloride, methyl, pyridine and hydroxyl groups in the binding of guest molecules. Through *in situ* synchrotron X-ray diffraction, neutron diffraction, IR, ^2^H NMR and neutron spectroscopic experiments, the binding of CO_2_ and SO_2_ has been comprehensively investigated in these two porous materials. All experiments show that the binding domains for CO_2_ and SO_2_ molecules are directly affected by the tuning of surface charge distribution and functional groups. Significantly, simultaneously enhanced adsorption capacity and selectivity have been observed on going to MFM-305. We also report a unique study of structural dynamics of restricted guest molecules in MOFs as a function of temperature, showing the unprecedented mobility of CO_2_ molecule within the pores.

## Results and discussion

### Structural analysis of MFM-305-CH_3_

**Fig. 1:**
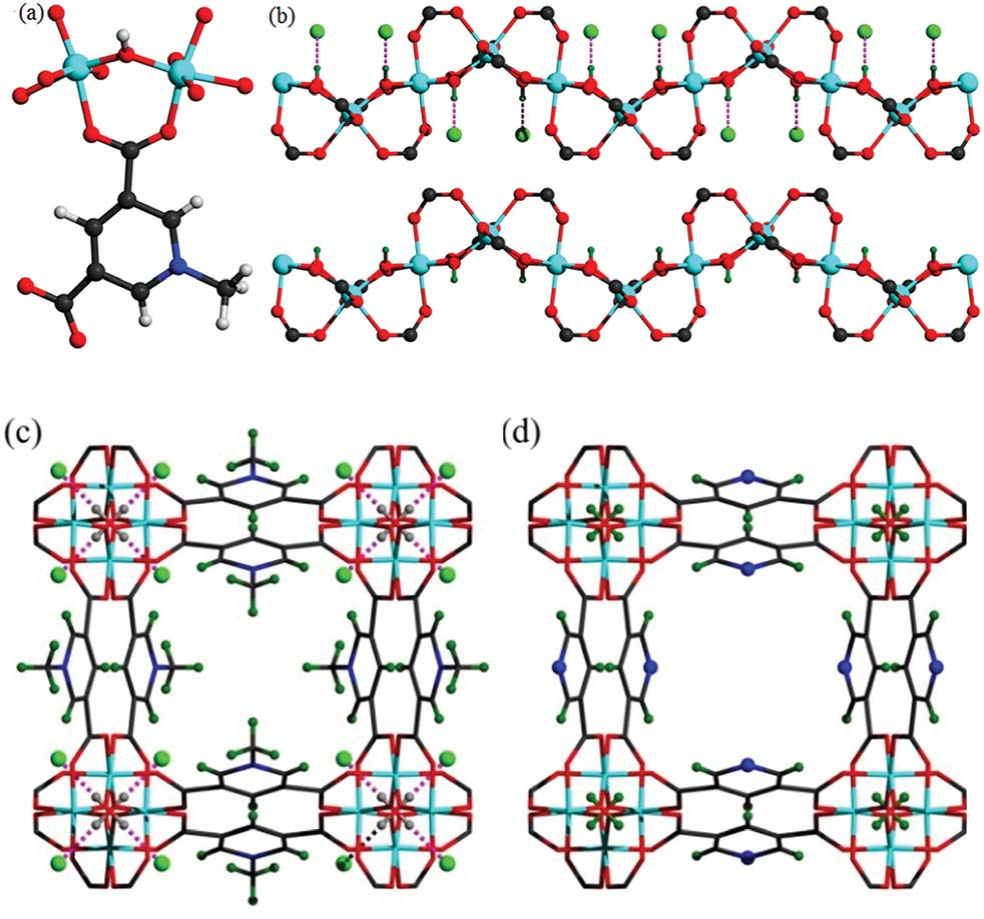
(a) View of the coordination environment for ligand L^–^ and the Al(iii) centre. (b) View of the corner-sharing octahedral chain of [AlO_4_(OH)_2_]···Cl. The μ_2_-(OH) groups form hydrogen bond to Cl^–^. Views of (c) MFM-305-CH_3_ and of (d) MFM-305. The pore size is ∼4.6 × 4.6 Å for MFM-305-CH_3_ and ∼5.6 × 5.6 Å for MFM-305 taking into consideration van der Waals radii. The methyl groups (olive) and chloride ions (green) in MFM-305-CH_3_; N atoms (blue) and hydroxyl group (olive) in MFM-305.

Solvothermal reaction of AlCl_3_·6H_2_O, [H_2_L]Cl ([H_2_L]Cl = 3,5-dicarboxy-1-methylpyridinium chloride) in a mixture of methanol and water (v/v = 12) at 130 °C for 3 days afforded MFM-305-CH_3_-solv, [Al(OH)(L)]Cl·solv, as a white crystalline powder in *ca.* 45% yield. The structure of MFM-305-CH_3_-solv has been determined by high resolution synchrotron X-ray powder diffraction (SPXRD). MFM-305-CH_3_-solv crystallizes in the space group *I*4_1_/*amd* and has an open structure comprising *cis*-connected, corner-sharing chains of [AlO_4_(OH)_2_]_∞_ bridged by dicarboxylate ligands. The Al^III^ centre shows octahedral coordination defined by four carboxylate oxygen atoms from the ligand [Al–O = 2.048(8) and 1.881(3) Å; each appears twice] and two oxygen atoms from two μ_2_-hydroxyl groups [Al–O = 1.776(6) Å]. Adjacent Al^III^ centers are linked by a μ_2_-hydroxyl group to form an extended chain of [AlO_4_(OH)_2_]_∞_ along the *c* axis. The ligands further bridge [AlO_4_(OH)_2_]_∞_ chains to give a three dimensional network with square-shaped channels filled with disordered guest solvents. The window size of the channel is approximately 4.6 × 4.6 Å taking van der Waals radii into consideration (Fig. 1 and S23†). Overall, the metal–ligand connection in MFM-305-CH_3_-solv is comparable to that in MFM-300 (ref. 21) and CAU-10.^34^ The total free solvent volume in MFM-305-CH_3_-solv was estimated by PLATON/SOLV to be 28.5%.^35^

Guest solvent molecules in the pores can be removed by heating at 110 °C under vacuum for 10 h to give the desolvated material MFM-305-CH_3_, which retains the structure of the solvated material as determined by SPXRD. As expected, the framework of desolvated MFM-305-CH_3_ is cationic since it incorporates pyridinium moieties, and these are balanced by chloride ions that hydrogen bond to the hydroxyl groups and aromatic –CH groups on the pyridinium ring [
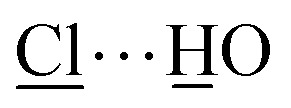
 = 2.01(1) Å; 
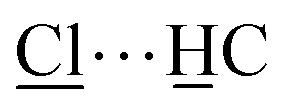
 = 2.47(1) Å].^36^,^37^ As a result, the μ_2_-OH groups in MFM-305-CH_3_ are hindered by the Cl^–^ ions and are thus not accessible to guest molecules, leaving 1-methylpyridinium and chloride ion as potential sites for guest interaction. The stability and rigidity of the framework in MFM-305-CH_3_ has been confirmed by variable temperature PXRD (50–550 °C) (Fig. S2†), which confirms framework decomposition above 450 °C.

### Structural analysis of MFM-305

TGA-MS measurements of desolvated MFM-305-CH_3_ shows that the methyl and chloride groups can be removed from the pore at 150–300 °C (Fig. S3†) giving the iso-structural neutral material MFM-305. To prepare a bulk sample of MFM-305, as-synthesized MFM-305-CH_3_-solv was heated at 180 °C under vacuum for 16 h to completely remove the guest solvents and CH_3_^+^/Cl^–^ (Fig. S4†). A change in color from white to pale yellow is observed on going from MFM-305-CH_3_ to MFM-305. SPXRD analysis of MFM-305 confirms that it retains the same space group *I*4_1_/*amd*, but shows a slight contraction along the *a*/*b* axis (*Δ* = 0.06%) and *c* axis (*Δ* = 4%) with an extended channel size of 5.6 × 5.6 Å. The total free solvent volume in MFM-305 was estimated by PLATON/SOLV to be 39.9%.^35^ The most significant change is conversion of the methylpyridinium species in MFM-305-CH_3_ to a free pyridyl moiety in MFM-305 and formal loss of CH_3_Cl. The bridging hydroxyl groups in MFM-305 are now exposed because of the removal of chloride (see below). Moreover, the pyridyl groups are now accessible within the pores of MFM-305, and IR spectroscopy confirms the loss of the stretching vibration of the –CH_3_ group at 2988 cm^–1^ on going from MFM-305-CH_3_ to MFM-305 (Fig. S7†). The complete removal of Cl^–^ in MFM-305 has been confirmed by XPS spectroscopy (Fig. S5 and S6†). Interestingly, the post-synthetic modification leads to distinct pore environments for isostructural MFM-305-CH_3_ and MFM-305, and thus provides an excellent platform to examine their capabilities of guest binding and selectivity. To the best of our knowledge, this is the first example of studying the mechanism of guest adsorption within an isostructural pair of MOFs with charged and neutral pore environments of this kind (Table 1).

**Table 1:** Unit cell parameters of MFM-305-CH_3_ and MFM-305

	MFM-305-CH_3_	MFM-305
Formula	Al(OH)(C_8_H_6_NO_4_)Cl	Al(OH)(C_7_H_3_NO_4_)
*M*_r_	259.6	209.1
Space group	*I*4_1_/*amd*	*I*4_1_/*amd*
*a*, *b* (Å)	21.48(6)	21.495(3)
*c* (Å)	10.90(3)	10.457(2)
Volume (Å^3^)	5030(30)	4831.6(1)
BET surface area/m^2^ g^–1^	256	779
Pore volume (*P*/*P*_0_ = 0.95)/cm^3^ g^–1^	0.181	0.372
Pore size (HK)/Å	5.2	6.2
Pore volume (Cal.)/cm^3^ g^–1^	0.209	0.347
Pore size (Cal.)/Å	4.6	5.6
CO_2_ uptake/mmol g^–1^ (273/298 K)	2.98/2.39	3.55/2.65
SO_2_ uptake/mmol g^–1^ (273/298 K)	5.29/5.16	9.05/6.99

### Gas adsorption analysis

**Fig. 2:**
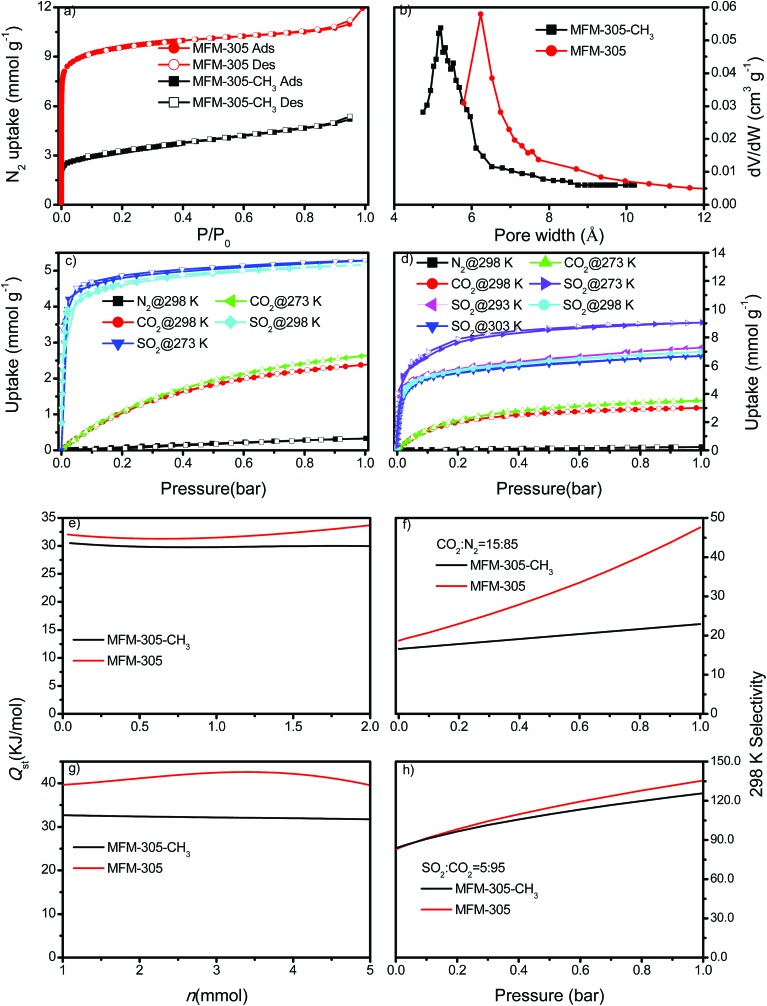
Gas adsorption data. (a) Adsorption isotherms for N_2_ in MFM-305-CH_3_ and MFM-305 at 77 K and 1.0 bar. (b) Comparison of the pore size of MFM-305-CH_3_ and MFM-305. (c) Adsorption isotherms for CO_2_, SO_2_ and N_2_ in MFM-305-CH_3_ at 273 and 298 K and 1.0 bar. (d) Adsorption isotherms for CO_2_, SO_2_ and N_2_ in MFM-305 at 273, 293, 298 and 303 K and 1.0 bar. Variation of isosteric heat of adsorption *Q*_st_ as a function of (e) CO_2_ and (g) SO_2_ uptake for MFM-305-CH_3_ and MFM-305 calculated from adsorption isotherms measured at 273 and 298 K. Comparison of the IAST selectivity of (f) CO_2_/N_2_ (15 : 85) and (h) SO_2_/CO_2_ (5 : 95) in MFM-305-CH_3_ and MFM-305 at 298 K.

N_2_ isotherms at 77 K for desolvated MFM-305-CH_3_ and desolvated MFM-305 both show type-I profiles confirming retention of microporosity. The BET surface area and total pore volume for MFM-305-CH_3_ are estimated to be 256 m^2^ g^–1^ and 0.181 cm^3^ g^–1^, respectively, and 779 m^2^ g^–1^ and 0.372 cm^3^ g^–1^, respectively, for MFM-305 confirming a ∼2.0 fold increase in porosity upon the modification (Fig. 2a, Table S1†). Horváth–Kawazoe (HK) analysis^38^ of the N_2_ isotherm confirms a pore size distribution centered at 5.2 and 6.2 Å for MFM-305-CH_3_ and MFM-305, respectively (Fig. 2b). The increase in pore size is consistent with those confirmed by X-ray structural analysis.

CO_2_ isotherms at 195 K and 1.0 bar show saturated uptake capacities of 4.23 and 5.69 mmol g^–1^ for MFM-305-CH_3_ and MFM-305, respectively (Fig. S24†). The increase (*ca.* 33%) is much smaller than that of N_2_ isotherm, consistent with the reduced kinetic diameter of CO_2_ (3.30 Å) comparing to N_2_ (3.89 Å). At 273 K and 298 K, MFM-305-CH_3_ shows CO_2_ uptakes of 2.67 and 2.41 mmol g^–1^, respectively, at 1.0 bar (Fig. 2c and d). In comparison, the values for MFM-305 were recorded as 3.55 and 3.02 mmol g^–1^, respectively. The CO_2_ uptake at 0.15 bar, which is relevant to its partial pressure in flue gas, are 0.86 mmol g^–1^ for MFM-305-CH_3_ and 1.76 mmol g^–1^ for MFM-305. This uptake is higher than that of H_3_[(Cu_4_Cl)_3_(BTTri)_8_]-en (BTTri = 1,3,5-tris(1*H*-1,2,3-triazol-5-yl)benzene) (0.682 mmol g^–1^)^39^ and NH_2_–MIL-101(Cr) (0.5 mmol g^–1^),^47^ but lower than that of MFM-300(Al) (2.64 mmol g^–1^),^21^ MOF-74(M) (M = Mg, Ni, Co) (5.3, 2.7, 2.7 mmol g^–1^, respectively)^48^ and the amine-modified MIL-101 (4.2 mmol g^–1^)^49^ under the same conditions.

The excellent stability of these two MOFs motivated us to measure the adsorption isotherm of SO_2_. At 298 K and 1.0 bar, MFM-305-CH_3_ and MFM-305 show SO_2_ adsorption capacities of 5.16 and 6.99 mmol g^–1^, respectively (Fig. 2c and d). Importantly, the SO_2_ uptake is fully reversible in both materials, and no loss of crystallinity or porosity was observed for the regenerated samples. At 298 K and 1.0 bar, the SO_2_ uptake of MFM-305 is notably higher than that of most solid sorbents in the literature (Fig. S29,† Table S2†), and only lower than that of MFM-300(In) (8.28 mmol g^–1^), Mg-MOF-74 (8.6 mmol g^–1^), Ni(bdc)(ted)_0.5_ (9.97 mmol g^–1^) (H_2_bdc = terephthalic acid; ted = triethylenediamine), MFM-202a (10.2 mmol g^–1^) and SIFSIX-1-Cu (11.01 mmol g^–1^), which have higher surface areas of 1071, 1525, 1783, 2220 and 3140 m^2^ g^–1^, respectively.^21^–^27^ At 303 K, MFM-305 shows an adsorption capacity for SO_2_ of 6.70 mmol g^–1^ at 1.0 bar, and a high uptake of 3.94 mmol g^–1^ at 0.025 bar, comparable to the best-behaving MOFs.^50^ Using the total pore volume, the storage density of SO_2_ in a given MOF system can be estimated; those for MFM-305-CH_3_, SIFSIX-1-Cu-i, MFM-305 and MFM-300(In) are calculated to be 1.83, 1.70, 1.20 and 1.27 g cm^–3^, respectively at 298 K and 1.0 bar. Thus, MFM-305-CH_3_ gives a notably higher storage density that is 70% of liquid density of SO_2_ at 263 K (2.63 g cm^–3^), indicating the presence of efficient packing of adsorbed gas molecules within the pore.

**Fig. 3:**
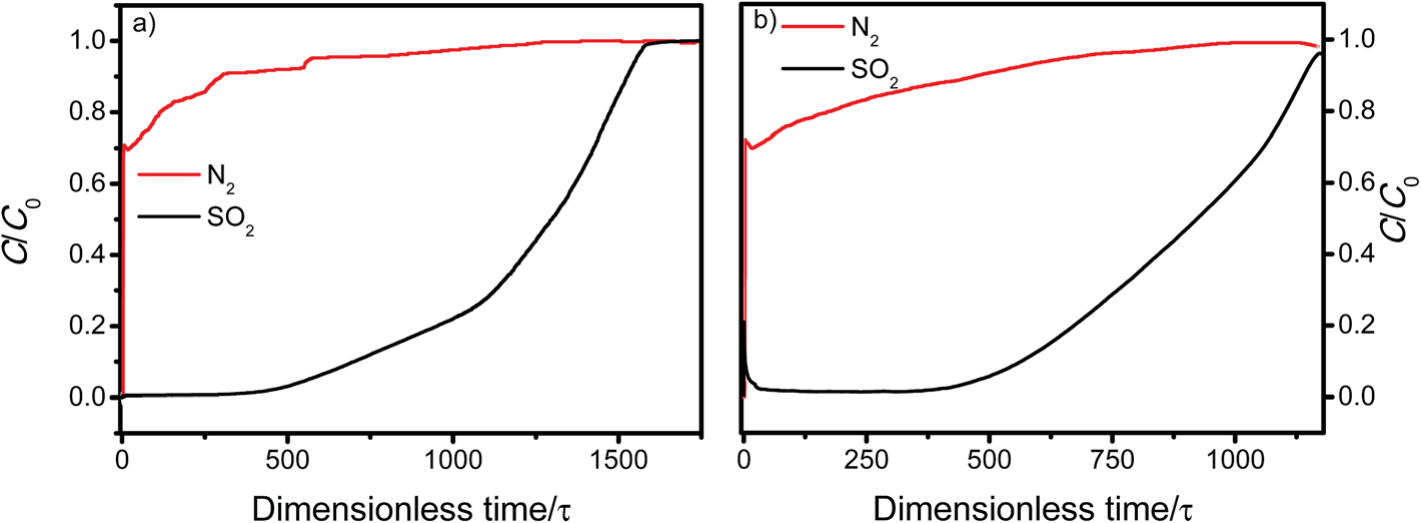
Dimensionless breakthrough curve of 0.25% SO_2_ (2500 ppm) diluted in He/N_2_ under (a) dry and (b) wet conditions through a fixed-bed packed with MFM-305 at 298 K and 1 bar.

Both MFM-305-CH_3_ and MFM-305 show highly selective SO_2_ uptakes. At 298 K, MFM-305-CH_3_ and MFM-305 exhibit very steep adsorption profiles for SO_2_ between 0 and 20 mbar, leading to an uptake of 3.59 and 3.94 mmol g^–1^, respectively, accounting for 70% and 56% of the total uptake at 1.0 bar. In contrast, under the same conditions, MFM-305-CH_3_ and MFM-305 show a much lower uptake of CO_2_ (*i.e.*, 0.15 and 0.40 mmol g^–1^, respectively). Moreover, the uptakes of N_2_ under the same conditions are negligible (<0.01 mmol g^–1^) in these two materials. The isosteric heat of adsorption (*Q*_st_) for CO_2_ in MFM-305-CH_3_ and MFM-305 both lie in the range of 29–34 kJ mol^–1^; on average the *Q*_st_ of MFM-305 is *ca.* 3 kJ mol^–1^ higher than MFM-305-CH_3_ (Fig. 2e). The *Q*_st_ for adsorption of SO_2_ in MFM-305 and MFM-305-CH_3_ is estimated to be 39–43 kJ mol^–1^ and 30–32 kJ mol^–1^, respectively (Fig. 2g). MFM-305 displays a higher *Q*_st_ value for SO_2_ uptake than MFM-305-CH_3_, indicating the presence of enhanced host–guest binding affinity upon the pore modification. To further evaluate their potential for gas separation, the selectivities for CO_2_/N_2_(*S*_CN_), SO_2_/CO_2_ (*S*_SC_) and SO_2_/N_2_ (*S*_SN_) at 298 K have been calculated using ideal adsorbed solution theory (IAST)^40^ over a wide range of molar compositions (*i.e.*, 1 : 99 to 50 : 50) (Fig. 2f, h, S31 and S32†). Significantly, for a 5 : 95 mixture of SO_2_/CO_2_ and a 15 : 85 mixture of CO_2_/N_2_, MFM-305-CH_3_ and MFM-305 both show exceptionally high IAST selectivities, and these are enhanced by the pore modification. The calculations for *S*_SN_ are subject to large uncertainties due to the extremely low uptake of N_2_. The adsorptive removal of low concentration SO_2_ by MFM-305 has been confirmed by breakthrough experiments in which a stream of SO_2_ (2500 ppm diluted in He/N_2_) was passed through a packed bed of MFM-305 under ambient conditions (Fig. 3a). As expected, He and N_2_ were the first to elute through the bed, whereas SO_2_ was retained selectively under dry condition (Fig. 3a). On saturation (dimensionless time > 500), SO_2_ breaks through from the bed and reaches saturation gradually. The ability of MFM-305 to capture SO_2_ in the presence of moisture has also been demonstrated by breakthrough experiments using a wet stream of SO_2_ (Fig. 3b). In the presence of water vapor, the breakthrough of SO_2_ from MFM-305 slightly reduces to 420 (dimensionless time) as a result of competitive adsorption of water. Thus, the marked differences in adsorption profiles, uptake capacities, *Q*_st_ between SO_2_, CO_2_ and N_2_, the corresponding IAST selectivity and the dynamic adsorption experiments indicate the potential of MFM-305-CH_3_ and MFM-305 have the potential to act as selective adsorbents for CO_2_ and SO_2_.

### Determination of binding domains for adsorbed CO_2_ and SO_2_

**Fig. 4:**
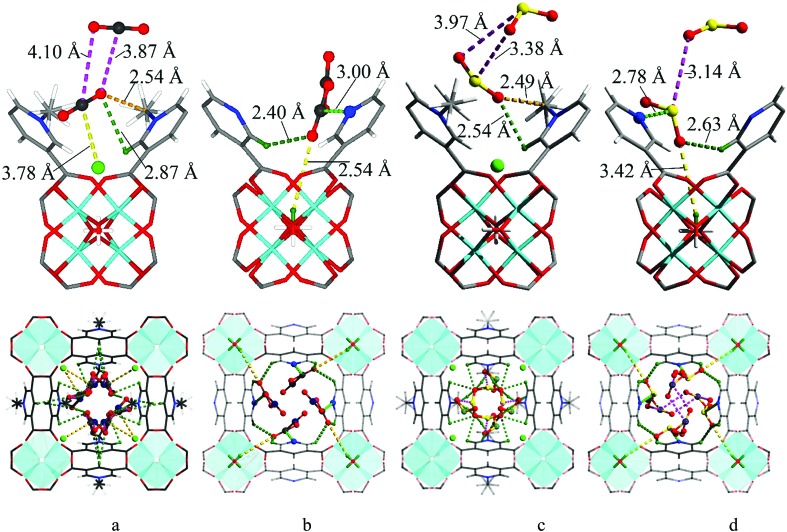
The crystal structures of CO_2_ and SO_2_-loaded MFM-305-CH_3_ and MFM-305 studied by powder diffraction at 298 K. (a) Interactions of CO_2_ molecule with the methyl, chloride ions and the –CH on pyridyl groups in MFM-305-CH_3_. (b) Interactions of CO_2_ molecule with the N- and C–H groups of pyridyl centre and the hydroxyl group in MFM-305. (c) Interactions of SO_2_ molecule with the methyl, chloride ions and the –CH on pyridyl groups in MFM-305-CH_3_. (d) Interactions of SO_2_ molecule with the N– and –CH centres of pyridyl and the hydroxyl group in MFM-305. Carbon, black; hydrogen, olive; oxygen, red; interactions between CO_2_ and frameworks C–H are shown in olive dashed line; interactions between adsorbed CO_2_ molecules and hydroxyl are shown in orange dashed line; interactions between adsorbed CO_2_ molecules and chloride ions are shown in yellow dashed line; interactions between adsorbed CO_2_ molecules and N atoms are shown in light green dashed line.

We sought to determine the binding domains for CO_2_ and SO_2_ in the pores of MFM-305-CH_3_ and MFM-305 since comparison between the binding sites within these two materials affords a direct observation of the effect of pore environment on guest binding. High resolution SPXRD data were collected at 198 K for CO_2_-loaded samples and at 298 K for SO_2_-loaded samples at 1 bar. SPXRD data enabled full structural analysis *via* Rietveld refinement (Fig. S8–S22†) to yield the positions, orientations and occupancies of adsorbed CO_2_ and SO_2_ molecules (Fig. 4 and S33†). Overall, all gas-loaded samples retain the *I*4_1_/*amd* space group and two crystallographically independent binding sites (I and II) are observed in each case.

In CO_2_-loaded MFM-305-CH_3_, COI2 (occupancy = 0.32) interacts with the methyl group with a H_3_C[combining low line]···O[combining low line]CO distance of 2.54(1) Å, indicating the formation of a weak hydrogen bond. COI2 also forms supramolecular interactions with aromatic –CH groups on neighboring pyridinium rings [OCO[combining low line]···H[combining low line]C = 2.87(2) Å]. Additionally, dipole interactions were observed between COI2 and Cl^–^ [
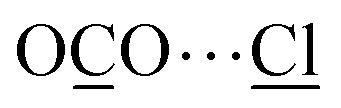
 = 3.78(1) Å, ∠O–C···Cl = 99.2(8)°].^30^,^41^ COII2 (occupancy = 0.41) binds to the aromatic –CH group [OCO[combining low line]^II^···H[combining low line]C = 3.50(1) Å]. Further dipole interactions were observed between COI2 and COII2 [O^I^···C^II^ = 3.87(1) Å; O^II^···C^I^ = 4.10(1) Å] in a typical T-shape arrangement.^42^

In CO_2_-loaded MFM-305, 
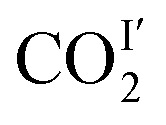
 (occupancy = 0.48) interacts with the hydroxyl group in an end-on mode [OH[combining low line]···O[combining low line]CO = 3.45(3) Å]. This distance is longer than that obtained for CO_2_-loaded MFM-300(Al) [2.298(4) Å] studied by PXRD at 273 K.^20^
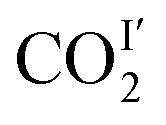
 also interacts with the exposed pyridine nitrogen atom *via* a dipole interaction [OC[combining low line]O···N = 2.89(1) Å]. Additionally, a series of weak supramolecular contacts of 
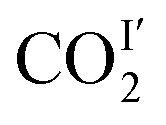
 to surrounding –CH groups [OCO[combining low line]···H[combining low line]C = 2.44(1); 2.73(1) Å] are also observed. Further dipole interactions are found between COI2 and COII2 with intermolecular C^I′^···O^II′^/C^II′^···O^I′^ distances of 3.09(1)/3.14(1) Å, comparable to that observed in dry ice [3.178(1) Å].^42^

At 298 K SO_2_-loaded MFM-305-CH_3_ shows the SOI2 molecule (occupancy = 0.30) binding to two adjacent methyl groups simultaneously [OSO[combining low line]···H_3_C[combining low line] = 2.49(3) Å]. SOI2 also forms hydrogen bonds with surrounding –CH groups [OSO[combining low line]···H[combining low line]C = 2.54(2) Å]. SOII2 (occupancy = 0.47) lies perpendicular to SOI2 [S^I^···O^II^/S^II^···O^I^ = 3.38(2) and 3.97(3) Å] and parallel to the pyridinium ring [OS[combining low line]O···HC[combining low line] = 3.95(1) Å]. The SO_2_ intermolecular distances within MFM-305-CH_3_ are comparable to that observed in the crystal structure of SO_2_ (3.10 Å),^43^ confirming the very efficient packing of adsorbed SO_2_ molecules leading to its high observed storage density.

In MFM-305, 
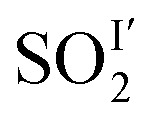
 (occupancy = 0.39) primarily binds to the pyridyl N-atom *via* a dipole interaction [OS[combining low line]O···N = 2.78(1) Å] and also forms hydrogen bonds with the exposed hydroxyl group [–OH[combining low line]···O[combining low line]SO = 3.42(4) Å] as well as the surrounding –CH groups [–CH[combining low line]···O[combining low line]SO = 2.63(3) and 3.14(3) Å]. Further dipole interactions were observed between SO_2_ molecules on sites I′ and II′ with intramolecular distance of 4.29(2) Å. Overall, these observations confirm that the methyl group and –CH groups are the primary binding sites in MFM-305-CH_3_ for both CO_2_ and SO_2_. In contrast, modulation of the pore environment in MFM-305 induces notable shifts of binding sites to the free pyridyl nitrogen center and hydroxyl group. This study offers a comprehensive understanding of the synergistic effect of functional groups on the binding of CO_2_ and SO_2_ in these materials.

### Analysis of host–guest binding *via* inelastic neutron scattering (INS)

**Fig. 5:**
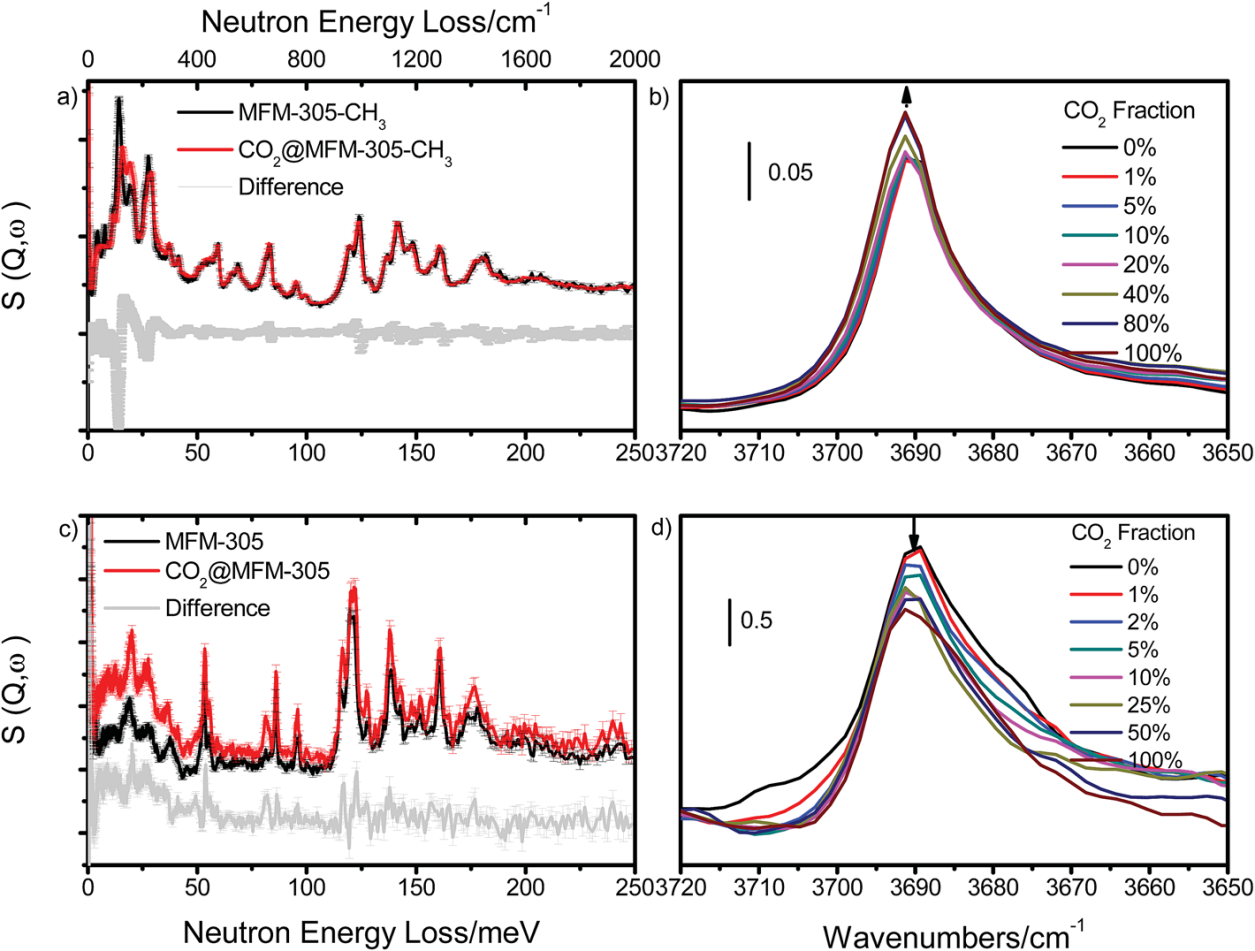
Comparison of (a and c) the INS spectra and (b and d) IR spectra for bare MFM-305-CH_3_ and MFM-305. INS and IR spectra offer vibrational information on the –CH_3_/–CH/–OH stretching and deformation region. (a) Comparison of the INS spectra for bare and CO_2_-loaded MFM-305-CH_3_. (b) IR spectra in the *ν*(μ_2_-OH) stretch region of CO_2_-loaded MFM-305-CH_3_. (c) Comparison of the INS spectra for bare and CO_2_-loaded MFM-305. (d) IR spectra of the *ν*(μ_2_-OH) stretch region of MFM-305 for CO_2_-loading. IR and INS data were collected at 298 K and 5 K, respectively.

To directly visualise the multiple supramolecular host–guest binding modes in these systems, INS spectra of bare and CO_2_-loaded MFM-305-CH_3_ and MFM-305 (1 gas/Al) were collected at 5 K (Fig. 5). In addition, the structural models obtained from X-ray crystallographic studies were optimized by DFT calculations. The corresponding DFT-calculated INS spectra show good agreement with the experimental data (Fig. S35–S39†). Comparison of the INS spectra of desolvated MFM-305-CH_3_ and MFM-305 shows (Fig. S35†) three distinct low-energy peaks at 13, 27, 36 and 19, 28, 37 meV, respectively, related to the lattice modes of MFM-305-CH_3_ and MFM-305. The most significant change is the absence of the –CH_3_ rotation/torsion mode at 20 meV in MFM-305, confirming full demethylation by the post-synthetic modification procedure. The groups of peaks observed at 40–85 meV and 85–200 meV in MFM-305-CH_3_ are assigned to wagging/bending modes of the bridging hydroxyl group and of the aromatic C–H bonds, respectively. As crystallographic studies confirm above, the hydroxyl groups within MFM-305-CH_3_ form hydrogen bonds to the chloride ion in the pores. Upon demethylation, the peaks at 40, 69 and 83 meV in MFM-305-CH_3_ shifts to higher energies at 53, 83, 115 and 119 meV in MFM-305, consistent with the removal of the chloride ions.

Addition of CO_2_ in MFM-305-CH_3_ is accompanied by significant change to peaks at 13 and 27 meV, indicating stiffening of the lattice modes as a result of CO_2_ inclusion. Large intensity changes were observed for the peaks at 20 meV, indicating the hindrance of rotation motion of –CH_3_ groups upon CO_2_ binding, consistent with the formation of hydrogen bonds as observed in the structural models. This is further accompanied by small red shifts for the peaks between 85 and 200 meV (bending modes of –CH groups). Thus, the INS result confirms that the methyl and –CH groups are the effective binding sites for CO_2_ in MFM-305-CH_3_. Addition of CO_2_ in MFM-305 leads to similar changes of peaks at 19, 28 and 37 meV. Significant changes to peak at 53, 83, 115 and 119 meV indicate that the bridging hydroxyl is directly involved in binding to CO_2_. The peaks between 120 and 200 meV also show notable changes in the C–H modes on CO_2_ binding. This result confirms unambiguously that the –OH group and pyridine ring are the primary binding site for CO_2_ in MFM-305, in excellent agreement with the crystallographic results. Thus, the INS results have confirmed the shifts of primary binding sites upon the modification of pore charge distribution.

### Analysis of CO_2_ binding *via in situ* synchrotron infrared micro-spectroscopy

In order to study the interaction between adsorbed CO_2_ molecules and the MOF hosts at 298 K, an *in situ* synchrotron IR micro-spectroscopic study^22^ was carried out as a function of CO_2_ loading. Upon desolvation of MFM-305-CH_3_ under a dry He flow, an absorption band at 3690 cm^–1^ corresponding to the *ν*(μ_2_-OH) stretching mode is observed. Upon dosing CO_2_ up to 1.0 bar, this peak remained at the same position, but the peak intensity increased slightly indicating a through-space effect due to the weak interaction between Cl^–^ and CO_2_ molecules (Fig. 5b). In contrast, upon dosing desolvated MFM-305 with 1.0 bar CO_2_, the peak at 3690 cm^–1^ shifts very slightly but the peak intensity decreases notably (Fig. 5d) indicating the presence of an strong interaction between CO_2_ and hydroxyl groups, entirely consistent with the crystallographic study. The combination bands of the –C=C– vibrations^44^ within MFM-305-CH_3_ centered at 1555 cm^–1^ shift to 1560 cm^–1^, and that at 1552 cm^–1^ in MFM-305 shifts to 1566 cm^–1^ upon CO_2_ loading, consistent with the formation of supplementary interactions between CO_2_ molecules and pyridinium or pyridyl rings (Fig. S40†). Thus, the observed changes in IR experiments gives further evidence and supports the distinct interactions between guest CO_2_ molecules and these two porous MOFs.

### Analysis of host–CO_2_ binding *via in situ*^2^H NMR spectroscopy

The –CH_3_ group in the 1-methylpyridinium dicarboxylate linker represents a fast stochastic rotor or natural isolated gyroscope. As such, its rotational parameters can be used to track the possible binding interaction with the guest molecules.^45^ We were interested to probe further the dynamic changes of the methyl groups on CO_2_ binding by synthesising MFM-305-CD_3_ and studying it by solid state ^2^H NMR spectroscopy over a wide temperature range (90–300 K). MFM-305-CD_3_ was obtained using the same synthetic route described above but using the deuterated ligand, 3,5-dicarboxy-1-methyl-*d*_3_-pyridinium chloride. Two samples were used in this ^2^H NMR study: guest-free MFM-305-CD_3_ and CO_2_-loaded MFM-305-CD_3_.

**Fig. 6:**
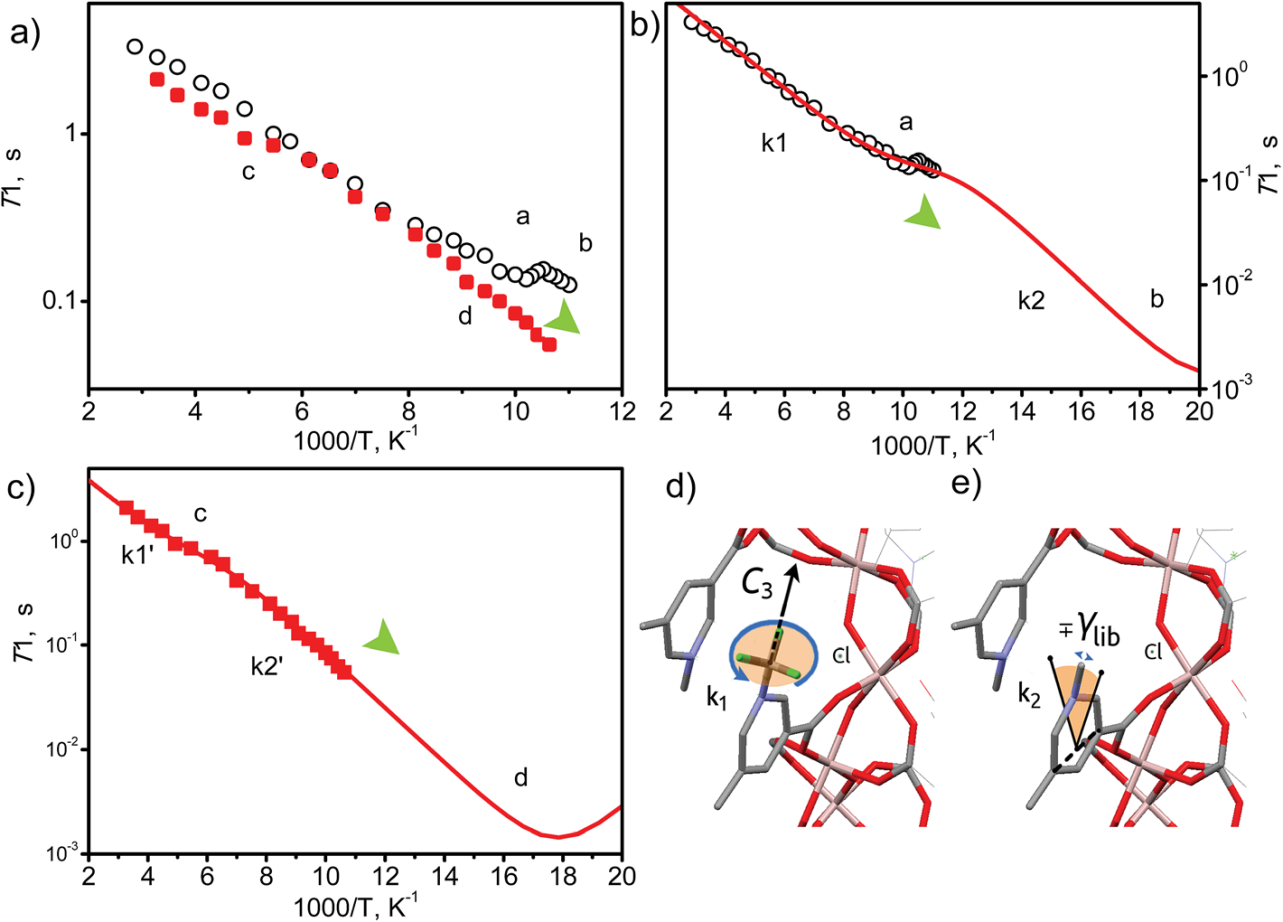
View of the temperature dependence of ^2^H NMR spin-lattice relaxation for –CD_3_ groups. (a) Experimental results for guest free MFM-305-CH_3_ (black circles) and for CO_2_@MFM-305-CH_3_ (1 mol per cavity) (red squares). (b and c) The simulated curve for MFM-305-CH_3_ and CO_2_@MFM-305-CH_3_ based upon two relaxation mechanisms corresponding to the two dynamic states of the –CD_3_ groups. (d and e) Representation of possible –CD_3_ motions in MFM-305-CH_3_ cavity.

Typically the –CD_3_ group is expected to exhibit very fast uniaxial rotation and hence its dynamics can be probed by ^2^H NMR spin-lattice (*T*_1_) relaxation, which is sensitive to rapid (rate > 10^6^ s^–1^) motions.^45^,^46^ The *T*_1_ relaxation curves (Fig. 6) show for both materials that the temperature dependence is characterised by two regions of monotonous decrease separated by a local minimum (marked as a and c in Fig. 6). Such behavior shows that in addition to the usual uniaxial rotations, the methyl groups perform another type of motion – a faster one, as it governs the relaxation curve notably at lower temperatures (marked b and d in Fig. 6). In CO_2_-loaded MFM-305-CD_3_, both modes are notably slower which is reflected in the behavior of the *T*_1_ curves behavior at ∼180 K and ∼100 K. A quantitative analysis requires a model of rotation (Fig. 6d and e): considering the close contact of the –CD_3_ group with the neighboring linker (*d* ∼ 3 Å), it is reasonable to assume that the slower (a, c) motion (*k*_1_, *k*_1_′) reflects the uniaxial rotation of the –CD_3_ group about its *C*_3_ symmetry axis aligned along the N–CD_3_ bond, while the faster (b, d) motion (*k*_2_, *k*_2_′) represents small angular librations of the rotating axis restricted within borders ±*γ*_lib_.

Parameters derived within such a model confirm that the rotation along N–CD_3_ axis (*E*_1_ = 4.2 kJ mol^–1^, *k*_10_ = 2.1 × 10^10^ s^–1^) reaches a rate of *k*_1_ ∼ 10^8^ s^–1^ at 100 K (a), while in the presence of the CO_2_ it is ∼10 times slower with *k*_1_′ ∼ 10^7^ s^–1^ (*E*_1_′ = 4.2 kJ mol^–1^, *k*_10_′ = 1.8 × 10^9^ s^–1^), and reaches 10^8^ s^–1^ at 180 K (c). Hence, by interacting with CO_2_, the pre-exponential factor of the –CD_3_ rotation is affected. This indicates that although the interaction with CO_2_ is not sufficient to increase the activation barrier of the rotation, the dynamic density of guests around the methyl groups is tight enough and sufficient to slow down the rotation rate by 10-fold by random collisions. Similarly, the libration mode is affected as well and the pre-exponential is faster and reaches its characteristic minimum^45^ below 90 K (b, d). For MFM-305-CD_3_ its rate at 100 K is *k*_2_ ∼ 10^11^ s^–1^, while for CO_2_-loaded MFM-305-CD_3_ it is notably slower and can be resolved unambiguously (*E*_2_′ = 5.1 kJ mol^–1^, *k*_20_′ = 0.65 × 10^13^ s^–1^) with *k*_2_′ ∼ 10^10^ s^–1^ (100 K). Notably, the amplitude of the restricted librations also sense the presence of CO_2_ with *γ*_lib_ ∼ 5° decreasing to *γ*_lib_′ ∼ 2°. This decrease in libration angle coupled with the strong deceleration of both motional modes by ∼10 times in the presence of CO_2_ evidences the interaction of CO_2_ guests with the methyl groups within the MOF pores.

### Structural dynamics of restricted CO_2_ molecules in the pore

**Fig. 7:**
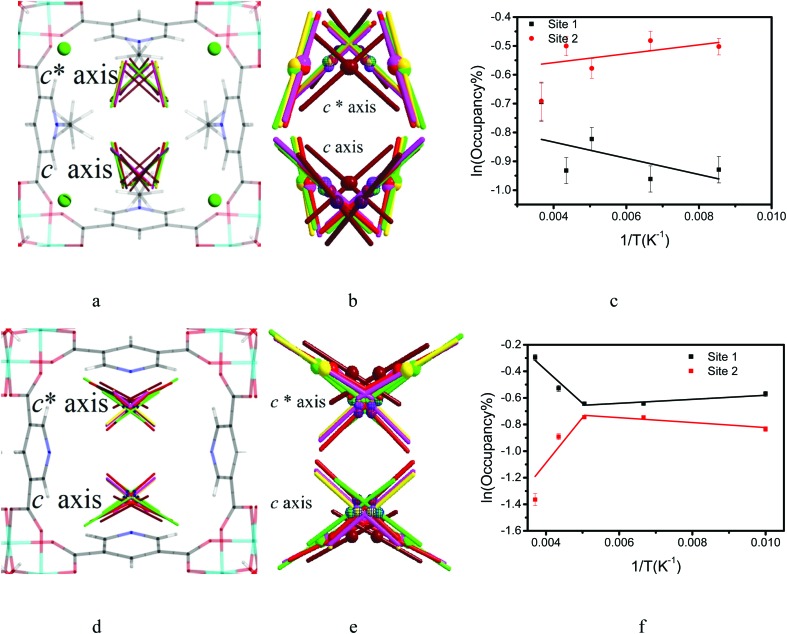
Comparison of the crystal structures of CO_2_-loaded MFM-305-CH_3_ and MFM-305 studied by synchrotron X-ray powder diffraction at variable temperatures. Dynamic structures of CO_2_ under different temperature (270 K, dark red; 230 K, red; 198 K, pink; 150 K, yellow; 117/100 K, bright green) in (a) MFM-305-CH_3_ and (d) MFM-305. (b and e) Enlarged views of the multiple CO_2_ regions in (a) and (d) at site I (solid symbol) and site II (patterned symbol). Occupancies of CO_2_ at site I and II in (c) MFM-305-CH_3_ and (f) MFM-305.

To investigate the dynamics of host–guest interaction, we sought to study the structural flexibility of restricted CO_2_ molecules (*e.g.*, positions, orientations and occupancies) within the pores of MFM-305-CH_3_ and MFM-305 *via* variable temperature SPXRD. *Le Bail* analysis reveals changes in lattice parameters of CO_2_-loaded samples as a function of temperature (Fig. 7, Table S3†). As the temperature decreases from 273 K to 117 K, the lattice parameters of CO_2_-loaded MFM-305-CH_3_ contract along all directions (Δ*V*/*V* = 0.7%), whereas MFM-305 expands along *c* axis (Δ*c*/*c* = 1.3%) and contracts along the *a*/*b* axes (Δ*a*/*a* = 0.02%) as the temperature decreases from 270 K to 100 K (Fig. S41†). Throughout the temperature range studied, two independent binding sites for CO_2_ molecules were observed in both samples. In general, the intermolecular distances of MOF–CO_2_ and CO_2_–CO_2_ decrease continuously as the temperature decreases (Tables S3 and S4†).

The CO_2_ supply was maintained at 1.0 bar on going from room temperature to 198 K. In MFM-305-CH_3_ the occupancy of CO_2_ (I + II) increased from 0.60 to 0.70 from 273 K to 230 K, with COI2 hydrogen bonding with the methyl group and the –CH groups on the pyridyl ring. The hydrogen bond distances [OCO[combining low line]^I^–H_3_C[combining low line]] decrease steadily from 3.10(2) to 2.38(2) Å from 273 K to 117 K, indicating that the strength of these hydrogen bonds is highly sensitive to temperature (Fig. 7, Table S4†). Interestingly, as the temperature decreases, we observe significant re-arrangement of restricted CO_2_ molecules at site I and II. COI2 molecules move closer to the pore surface and rotate to drive the oxygen atoms closer to the ligands to form stronger hydrogen bonds. COI2 and COII2 retain their T-shape arrangement, but move closer to each other. From 273 K to 117 K, the ratio [*i.e.* COI2/(COI2 + COII2) × 100%] of the occupancy of site I decreases from 50(2)% to 40(2)%, while the occupancy of site II increases from 50(2)% to 60(1)%. The activation energy (*E*_a_) of the site configuration and occupancy was calculated using the Arrhenius equation as a function of temperature. From 273 K to 117 K, *E*_a_ for site I is 0.233 kJ mol^–1^, and for site II it is –0.128 kJ mol^–1^. The changes of site configuration and occupancy reveal that the host–guest binding is highly sensitive to temperature *via* intra-pore re-arrangement.

Variable temperature PXRD data were collected for MFM-305 using the same method. 
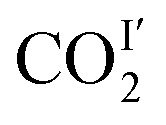
 forms hydrogen bonds with hydroxyl group and the –CH groups of the pyridyl ring, and the free N-center of the pyridyl ring interacts with CO_2_*via* dipole interactions. The hydrogen bond distance (OCO[combining low line]^I′^–H[combining low line]O) and the distance of CO_2_ to the N-center (N–OC[combining low line]O^I′^) both reduce from 4.20(3) to 3.34(4) and from 3.16(1) to 2.96(1) Å, respectively, as the temperature is lowered from 270 K to 100 K. The intermolecular distances between 
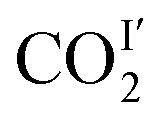
 and 
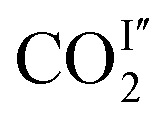
 (OCO[combining low line]^I′^–OC[combining low line]O^II′^ and OC[combining low line]O^I′^–O[combining low line]CO^II′^) reduce from 5.03(2) to 3.07(1) Å and 5.04(3) to 2.94(1) Å, respectively, from 270 to 100 K (Fig. 7, Table S4†). From 270 K to 198 K, the total CO_2_ occupancy increases from 0.5 to 0.92, which was retained to 100 K. From 270 K to 198 K, the ratio of 
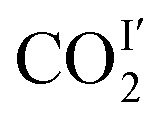
 decreases from 74(1)% to 53(1)%, and that of 
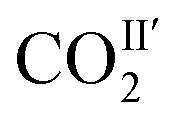
 increases from 26(1) to 47(1)%. Below 198 K, the ratio of 
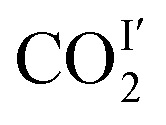
 increase slightly to 57(1)% and that of 
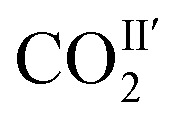
 decreases to 43(1)%. 
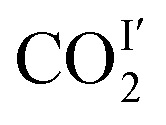
 is in a cross-tunnel mode at 270 K. 
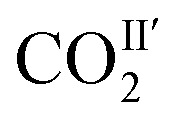
 molecules are parallel to the pyridine ring at 270 K, and rotate to be almost parallel to 
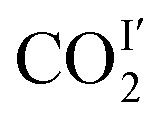
 with reducing temperature. From 270 K to 198 K, the *E*_a_ of 
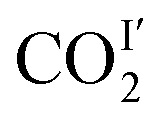
 and 
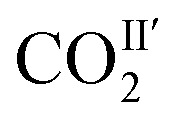
 are 2.068 and –2.842 kJ mol^–1^, respectively, and from 198 K to 100 K, the *E*_a_ values are –0.125 kJ mol^–1^ and 0.154 kJ mol^–1^ for 
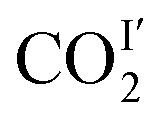
 and 
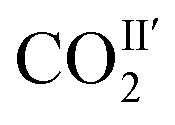
, respectively. The *E*_a_ for adsorbed CO_2_ molecules in MFM-305 is significantly higher than that of MFM-305-CH_3_ at 270–198 K, indicating the formation of stronger host–guest interactions in MFM-305. This study confirms that the host–guest interaction between functional groups and CO_2_ molecules in these two MOFs follows the trend of hydroxyl/pyridyl groups–CO_2_ > CO_2_–CO_2_ > methyl group–CO_2_. Thus, the simultaneous enhancements of adsorption capacity and host–guest binding affinity upon pore modification on going from MFM-305-CH_3_ to MFM-305 can be fully rationalised.

The crystal structure of CO_2_-loaded MFM-305-CH_3_ was also determined at 7 K by neutron powder diffraction which confirms retention of the space group *I*4_1_/*amd*. Interestingly, a completely new structure was resolved where only one binding domain for adsorbed CO_2_ was located near the methyl group (Fig. S34†). The adsorbed CO_2_ molecules are disordered about a 2-fold rotation axis and in a cross-tunnel mode interacting with the methyl group with a C[combining low line]H_3_···O[combining low line]CO distance of 3.58(2) Å. CO_2_ also binds to the pyridinium–hydrogen atoms with a CH[combining low line]···O[combining low line]CO distance at 3.08(2) Å. This result confirms the significant impact of temperature on the binding sites and orientation of adsorbed CO_2_ molecules in MFM-305-CH_3_.

## Conclusions

We have reported here the synthesis and characterisation of porous MFM-305-CH_3_, and its transformation *via* post-synthetic demethylation to give the isostructural, neutral MFM-305. The post-synthetic modification has enabled direct modulation of the pore environment, including changes in charge distribution and accessible functional groups. Significantly, MFM-305 shows simultaneously enhanced CO_2_ and SO_2_ uptake and CO_2_/N_2_ and SO_2_/CO_2_ selectivities over MFM-305-CH_3_; these two factors are widely known to display a trade-off in porous materials. A comprehensive investigation of the host–guest binding using a combinations of synchrotron X-ray and neutron powder diffraction, INS, ^2^H NMR and IR spectroscopy and modelling has unambiguously revealed the role of Lewis acid, Lewis base, chloride ions, methyl and hydroxyl groups in the supramolecular binding of guest molecules within the pores of both MOFs. Considering that these two MOFs have similar pore shape and size, the distinct binding mechanisms to guest molecules between these two samples is a direct result of the charge modulation. We have also confirmed that post-synthetic modification *via* dealkylation of the as-synthesised metal–organic framework is a powerful route to the synthesis of materials incorporating active polar groups, in this case a free pyridyl N-donor. Thus, deprotection of the as-synthesized MOF allows the synthesis of materials that cannot as yet, in our hands, be synthesized directly.

## Conflicts of interest

The authors declare no competing financial interests.

## Supplementary Material

Supplementary information

Crystal structure data
