# Supplementary material for: Post-synthetic modulation of the charge distribution in a metal–organic framework for optimal binding of carbon dioxide and sulfur dioxide
Source: Chem Sci. 2018 Oct 31;10(5):1472–82. doi: 10.1039/c8sc01959b (PMC6369579; doi:10.1039/c8sc01959b)
Supplement: Supplementary file 1 [file SC-010-C8SC01959B-s001.pdf]

Electronic Supplementary Material (ESI) for Chemical Science.
This journal is © The Royal Society of Chemistry 2018

Supplementary Information

Post-synthetic Modulation of the Charge Distribution in a Metal-Organic Framework for Optimal Binding of Carbon Dioxide and Sulfur Dioxide

Lei Li,[1,2] Ivan da Silva,[3] Daniil I. Kolokolov,[4,5] Xue Han,[1] Jiangnan Li,[1] Gemma Smith,[1] Yongqiang Cheng,[6] Luke L. Daemen,[6] Christopher G. Morris,[1,7] Harry G.W. Godfrey,[1] Nicholas M. Jacques,[1] Xinran Zhang,[1] Pascal Manuel,[3] Mark D. Frogley,[7] Claire A. Murray,[7] Anibal J. Ramirez-Cuesta,[6] Gianfelice Cinque,[7] Chiu C. Tang,[7] Alexander G. Stepanov,[4,5] Sihai Yang[1]* and Martin Schröder[1]*

[1] School of Chemistry, University of Manchester, Oxford Road, Manchester, M13 9PL (UK) Sihai.Yang@manchester.ac.uk, M.Schroder@manchester.ac.uk

[2] Lehn Institute of Functional Materials, School of Chemistry, Sun Yat-Sen University, Guangzhou, 510275 (China)

[3] ISIS Neutron Facility, STFC Rutherford Appleton Laboratory, Chilton, Oxfordshire, OX11 0QX (UK)

[4] Boreskov Institute of Catalysis, Siberian Branch of Russian Academy of Sciences, Prospekt Akademika Lavrentieva 5, Novosibirsk, 630090 (Russia)

[5] Novosibirsk State University, Novosibirsk 630090 (Russia)

[6] The Chemical and Engineering Materials Division (CEMD), Neutron Sciences Directorate, Oak Ridge National Laboratory, Oak Ridge, TN 37831 (USA)

[7] Diamond Light Source, Harwell Science Campus, Oxfordshire, OX11 0DE (UK)

EXPERIMENTAL SECTION

Materials and measurements

All reagents were commercially available and used as received. Powder X-ray diffraction (PXRD) patterns were recorded on a Phillips PANalytical diffractometer for Cu-Kα radiation (λ = 1.5406 Å) at a scan rate of 2° min^{-1} and a step size of 0.02° in 2θ. Variable-temperature PXRD were carried out under vacuum on a Phillips PANalytical diffractometer. The diffractograms were recorded in the low angle 2θ range, heating *in situ* by increments of 50°C until significant loss of crystallinity was observed. Fourier transform infrared (FT-IR) spectra were measured on a Nicolet Avatar 360 FT-IR spectrophotometer in the 400-4000 cm^{-1} region. Thermal gravimetric analyses (TGA) were performed under N_2 (100 ml/min) with a heating rate of 2°C/min using a TA SDT-600 thermogravimetric analyzer in the range of 25-800 °C. X-Ray photoelectron spectra (XPS) were measured on a Kratos Axis Ultra X-ray photoelectron spectrometer equipped with an aluminium/magnesium dual anode and a monochromated aluminium X-ray source.

Synthesis of ligand

3,5-Dicarboxy-1-methylpyridinium chloride was synthesized according to a previously reported procedure.[1] Diethyl pyridine-3,5-dicarboxylate hydrochloride (10.0 g, 23.79 mmol) and iodomethane (10mL) were placed into a 250 mL round bottomed flask and dissolved in a mixture of toluene and acetonitrile (2:1, 90mL). The flask was stirred overnight at 65°C and the solvent removed under vacuum to give a residue which was dissolved in 100 mL of 50% HCl. This aqueous solution was heated at 90°C for 7 days after which the solvent was removed under vacuum and the residue washed with acetone. The white product was dried at 60 °C (isolated yield: 5.0 g, 90%). ^{1}H NMR (300 MHz, D_2O): δ 9.17 (s, 2H,), 9.11 (s, 2H), 4.400 (s, 3H), MS (ESI) *m*/*z* $(M\text{-}Cl)^+$: 182.109 (100%).

3,5-Dicarboxy-1-methyl(d_3)pyridinium chloride was synthesized according to the same procedure but starting with iodomethane-d_3.

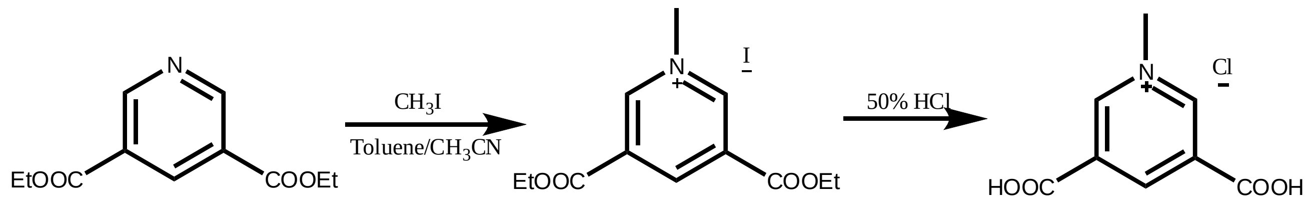

Synthesis of MFM-305-CH_3

3,5-Dicarboxy-1-methylpyridinium chloride (84 mg, 0.4 mmol) and $AlCl_3 \cdot 6H_2O$ (46.2 mg, 0.2 mmol) were placed in a Teflon lined stainless-steel autoclave and dissolved in a mixture of MeOH and H_2O (1.235/0.103 mL). The vial was sealed and heated at 130 °C for 3 days. The material was obtained as white powder and washed with MeOH and dried in air. (isolated yield = 45%). Elemental Analysis for MFM-305-$CH_3 \cdot 3H_2O$: [found (calc.%)]: C 31.64(30.64), H 4.01(4.18), N 5.4(4.47), Cl 12.20(11.30).

Synthesis of MFM-305-CD_3

Deuterated MFM-305-CD_3 was synthesized according to the same procedure as above but using 3,5-dicarboxy-1-methyl(d_3)pyridinium chloride.

Synthesis of MFM-305

MFM-305 was synthesized by heating MFM-305-CH_3 at 180 °C under vacuum for 16 h. Elemental analysis for MFM-305$\cdot 3H_2O$: [found (calc.%)]: C 31.32(31.95), H 3.92(3.83), N 5.00(5.32).

Gas Adsorption Isotherms

Sorption isotherms for N_2, CO_2 and SO_2 were recorded at 77 K (liquid N_2), 195 K (acetone-dry ice) or at 273-298 K (temperature-programmed water bath) using a 3Flex system (Micromeritics Instrument Corporation, USA) and a Xemis (Hiden) under ultra-high vacuum from a diaphragm and turbo pumping system. All gases used were ultra-pure research grade (99.999%) purchased from BOC or Air Liquide.

Calculation of isosteric heats of adsorption

$$\ln P = \ln N + \sum_{i=0}^{m} a_i N^i + \sum_{i=0}^{n} \binom{n}{k} b_i N^i$$

$$Qst = -R \sum_{i=0}^{m} a_i N^i$$

A virial-type expression of the above form was used to fit the combined isotherm data at 273 and 298 K, where P is the pressure, N is the adsorbed amount in mmol/g, T is the temperature in K, a_i and b_i are virial coefficients, and m and n are the number of coefficients used to describe the isotherms. Q_{st} is the coverage-

dependent enthalpy of adsorption and R is the universal gas constant.

Analysis and Derivation of the Ideal Adsorption Solution Theory (IAST) Selectivity

Ideal Adsorbed Solution Theory (IAST) was used to determine the selectivity factor, S, for binary mixtures using pure component isotherm data. S is defined according the equation,

$$S = \frac{x_1 / y_1}{x_2 / y_2}$$

where x_i is the amount of each component adsorbed, as determined by IAST and y_i is the mole fraction of each component in the gas phase at equilibrium. The IAST adsorption selectivities were calculated for binary mixture of CO_2/N_2, SO_2/N_2 at concentrations of 1:99 to 50:50 at 298 K up to a pressure of 1 bar.

Breakthrough experiments

Breakthrough experiments were carried out in a 7mm diameter tube of 120 mm length packed with ~3 g of MFM-305 powder (particle size < 5 μm). The total volume of the bed was about 5 cm^3. The sample was heated at 100 °C under a flow of He for 2 days for complete activation. The fixed bed was then cooled to room temperature (298 K) using a temperature-programmed water bath and the breakthrough experiment performed with a stream of 0.25% SO_2 (diluted in He and N_2) at atmospheric pressure and room temperature. The flow rate of the entering gas mixture was maintained at 40 ml min^{-1}, and the gas concentration, C, of SO_2 and N_2 at the outlet was determined by mass spectrometry and compared with the corresponding inlet concentration C_0, where $C/C_0 = 1$ indicates complete breakthrough.

***In situ* neutron powder diffraction (NPD)**

Prior to NPD experiments, MFM-305-CH_3 was thermally activated at 110 °C for 96 h under dynamic vacuum. Time-of-flight NPD measurements were carried out on the GEM diffractometer at the pulsed neutron source at the ISIS Facility, Rutherford Appleton Laboratory, UK. The solvent-free material, 1.7 g, was loaded into a vanadium cell, and the sample cooled *in situ* using a He cryostat. The NPD measurements were performed at 7K. Diffraction data as a function of neutron time-of-flight were collected from the sample in five GEM detector banks centred at 2θ = 9.1, 18.0, 35.1, 63.9, and 94.4°. Data were normalized to the wavelength distribution of the incident neutron beam, and focused into 5 separate histograms, one for each detector bank.

After collecting the NPD data for the bare material, CO_2 loadings up to 1.0 CO_2 per Al were introduced from a calibrated volume. The CO_2 loadings were performed at 298 K and the sample cooled to 7 K after all the gas had been adsorbed, allowing sufficient time to achieve thermal equilibrium before data collection.

In situ **powder diffraction data collection and Rietveld refinement**

High resolution *in situ* synchrotron powder X-ray diffraction (PXRD) data were collected at Beamline I11 of Diamond Light Source using multi-analysing crystal-detectors and monochromated radiation (λ = 0.825582 Å) . These *in situ* diffraction measurements were carried out in capillary mode and the temperature controlled by an Oxford Cryosystems open-flow nitrogen gas cryostat. In a typical experiment, the powder sample of MFM-305 was dried in air and ground for 10 min before loading into a capillary tube (0.7 mm diameter). The capillary tube was connected to high vacuum (10^{-5}mbar) and heated at 130 ℃ for ~4 h to generate fully desolvated MFM-305. After collecting the HR-PXRD data for the bare material, gas was loaded up to 1 bar at 298 K. After dosing, ~30 mins were allowed for equilibration and the sample was then cooled to measurement temperature, allowing sufficient time to achieve thermal equilibrium before data collection. The powder pattern of MFM-305-CH_3, MFM-305, CO_2@MFM-305-CH_3, SO_2@MFM-305-CH_3, CO_2@MFM-305, SO_2@MFM-305 were collected.

Variable temperature *in situ* synchrotron X-ray diffraction data were also collected at Beamline I11 of Diamond Light Source. These *in situ* diffraction measurements were carried out in capillary mode with a CO_2 supply line which can control the pressure accurately, and the temperature was controlled by an Oxford Cryosystems open-flow N_2 cryostat. In a typical experiment, the powder sample was loaded into a capillary tube (0.7 mm diameter) which was connected to high vacuum (10^{-5} mbar) and heated at 130 ℃ for ~4 h to generate the fully desolvated material. Gas was loaded into the sample up to 1 bar at room temperature and the CO_2 supply was then sealed to prevent the formation of dry ice in the sample cell. After dosing, ~30 min were allowed for equilibration, sufficient time to achieve thermal equilibrium. The sample was then cooled for data collection.

Inelastic neutron scattering

INS spectra were recorded on the TOSCA spectrometer at the ISIS Facility at the Rutherford Appleton Laboratory, UK, and at the VISION spectrometer at Oak Ridge National Laboratory, USA for energy transfers between ~-2 and 500 meV. Desolvated MFM-305-CH_3 and MFM-305 were loaded into a cylindrical vanadium

sample container with an annealed copper vacuum seal and connected to a gas handling system. The sample was degassed at 10^{-7} mbar and 130 °C for 4 days to remove any remaining trace of guest water molecules. The temperature during data collection was controlled using a helium cryostat (5 ± 0.2 K). The loading of CO_2 was performed at room temperature in order to ensure that CO_2 was present in the gas phase when not adsorbed and also to ensure sufficient mobility of CO_2 inside the crystalline structure of MFM-305-CH_3 and MFM-305. The temperature was reduced to below 5 K in order to perform the scattering measurements with the minimum achievable thermal motion for CO_2. Background spectra were subtracted to obtain the difference spectra. INS was used to study the binding interaction and structure dynamics in these gas loaded materials.

DFT Calculations for INS Spectroscopy

Modelling using Density Functional Theory (DFT) of the bare and CO_2-loaded MOFs was performed using the Vienna Ab initio Simulation Package (VASP).[2] The calculation used the Projector Augmented Wave (PAW) method[3,4] to describe the effects of core electrons, and Perdew-Burke-Ernzerhof (PBE)[5] implementation of the Generalized Gradient Approximation (GGA) for the exchange-correlation functional. Energy cutoff was 500 eV for the plane-wave basis of the valence electrons. The lattice parameters and atomic coordinates determined by NPD in this work were used as the initial structure. Some of the CO_2 sites have partial occupancy, and to account for this properly a supercell calculation would be desirable, but too costly in practice. Instead, a single unit cell was used and the partially occupied sites modified to be either occupied or unoccupied, according to their local environment and symmetry (there needs to be either a complete CO_2 molecule or no molecule, and the overall probability of being occupied needs to be proportional to the actual occupancy). The total energy tolerance for electronic energy minimization was 10^{-8} eV, and 10^{-7} eV for structure optimization. The maximum interatomic force after relaxation was below 0.001 eV/Å. The optB86b-vdW functional[6] was applied for dispersion corrections. The vibrational eigen-frequencies and modes were then calculated by solving the force constants and dynamical matrix using Phonopy.[7] The aClimax software[8] was used to convert the DFT-calculated phonon results to the simulated INS spectra.

***In situ* Solid state ^{2}H NMR Spectroscopy**

^{2}H NMR spectroscopic experiments were performed at Larmor frequency $\omega_Z/(2\pi) = 61.42$ MHz on a Bruker Avance 400 spectrometer using a high power probe with 5 mm horizontal solenoid coil. All ^{2}H NMR spectra were obtained by Fourier transformation of quadrature-detected phase-cycled quadrupole echo arising

in the pulse sequence ($90°_x$–τ_1–$90°_y$–τ_2– acquisition – t), where τ_1 = 20 μs, τ_2 = 22 μs, and t is a repetition time of the sequence during the accumulation of the NMR signal. The duration of the π/2 pulse was 1.6μs. Spectra were obtained typically with 50–1000 scans with repetition time ranging from 1 to 15 s. Inversion-recovery experiments to derive spin-lattice relaxation times (T_1) were carried out using the pulse sequence ($180°_x$ – t_v – $90°_x$ – τ_1 – $90°_y$ – τ_2 – acquisition– t), where t_v is the variable delay between the 180° and 90° pulses. The sample was then heated at 433 K for 24 h under vacuum of 10^{-5} Torr (1 Torr = 133.3 Pa). After the sample was cooled to room temperature, the sample was loaded with a defined amount of CO_2 and sealed. The loading was ~1.0 molecules per Al(III) center of the sample. The adsorption was performed from the gas phase with the powder at 77K. The sealed gas-loaded sample was then left at room temperature for several days to obtain a uniform distribution of the adsorbed CO_2 over the sample. The temperature of the samples was controlled with a flow of N_2 gas using a variable-temperature unit BVT-3000 with a precision of about 1 K. The ^2H NMR line shape analysis and numerical simulation of the spin-lattice relaxation (T_1) temperature dependence curves were performed using in-house FORTRAN routines based on the general formalism reported elsewhere.

***In situ* IR spectroscopy**

In situ IR spectroscopic studies on MFM-305-CH_3 and MFM-305 were carried out on the Multimode InfraRed Imaging and Microspectroscopy (MIRIAM) beam line at Diamond Light Source. The instrument is comprised of a Bruker Hyperion 3000 microscope in transmission mode with a 15x objective and a liquid N_2 cooled MCT detector coupled to a Bruker Vertex 80 V Fourier Transform IR interferometer using radiation generated from a bending magnet source. Spectra were collected (256 scans) in the range 650-4000 cm^{-1} at 4 cm^{-1} resolution and an infrared spot size at the sample of approximately 25 × 25 μm. MFM-305-CH_3 and MFM-305 were placed onto a ZnSe disk and placed within a Linkam FTIR 600 gas-tight sample cell equipped with ZnSe windows, a heating stage and gas inlet and outlets. He and CO_2 were pre-dried using individual zeolite filters. The analysis gases were dosed volumetrically into the sample cell using mass flow controllers, the total flow rate being maintained at 100 cm^3 min^{-1} for all experiments. The gases were directly vented to an exhaust system, and the total pressure in the cell was 1 bar for all experiments. Dry He and CO_2 were dosed as a function of partial pressure maintaining a total flow of 100 cm^3 min^{-1}.

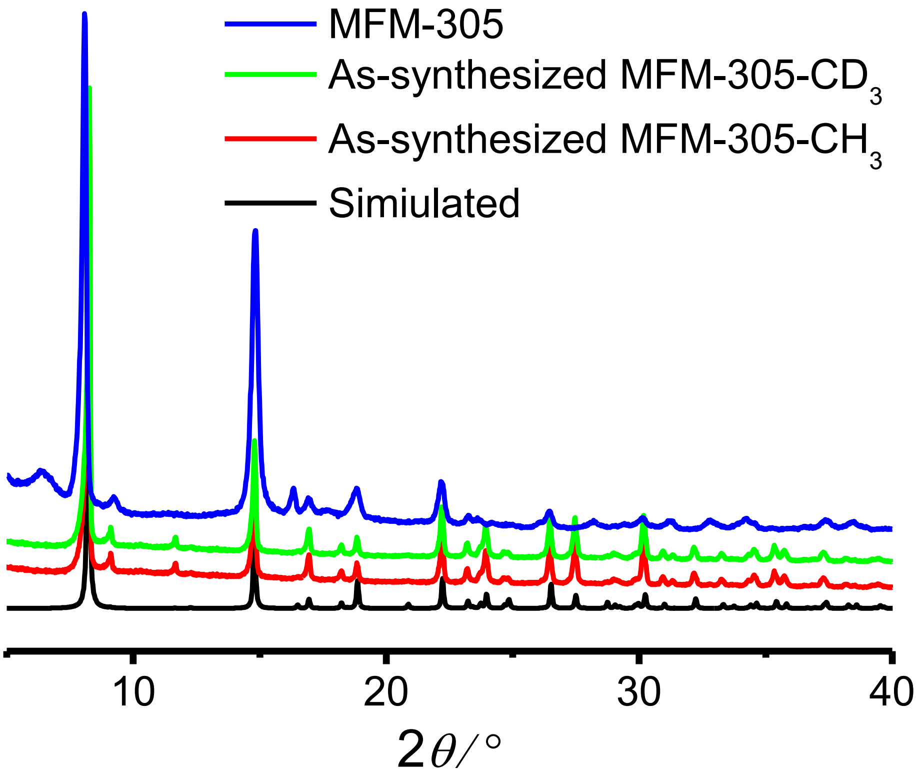

Figure S1. PXRD patterns for MFM-305-CH_3 and MFM-305.

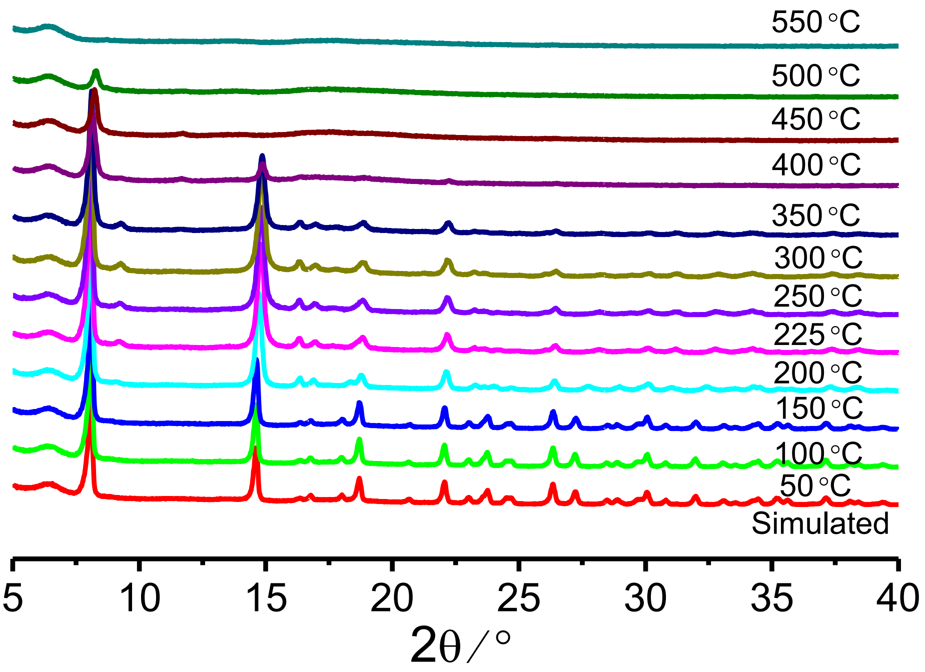

Figure S2. Variable temperature PXRD patterns for MFM-305-CH_3.

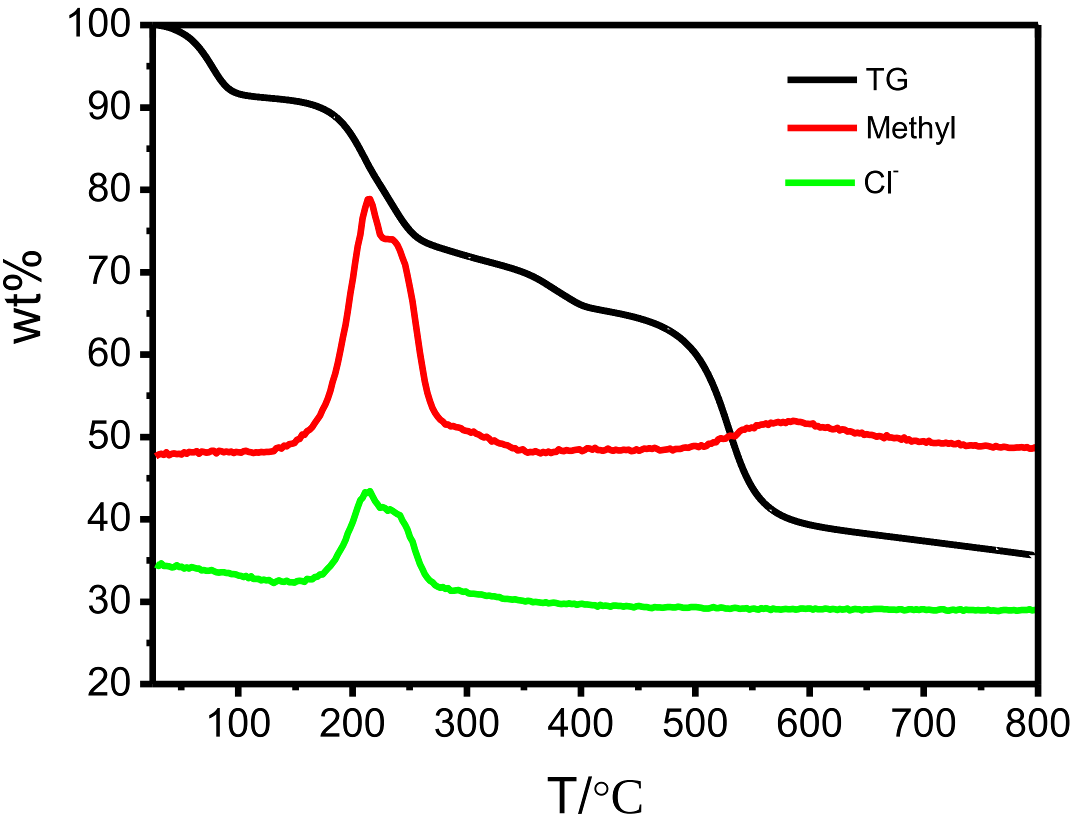

Figure S3. Thermogravimetric and mass spectrometric data for MFM-305-CH_3.

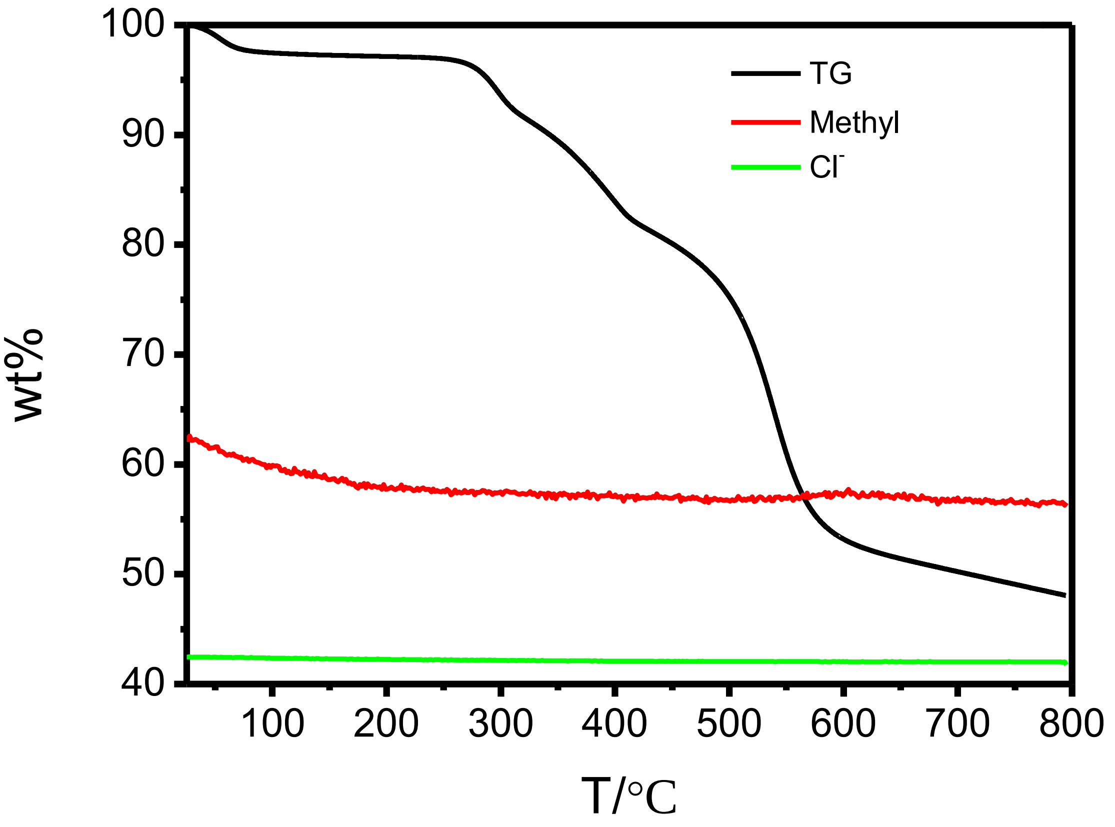

Figure S4. Thermogravimetric and mass spectrometric data for MFM-305.

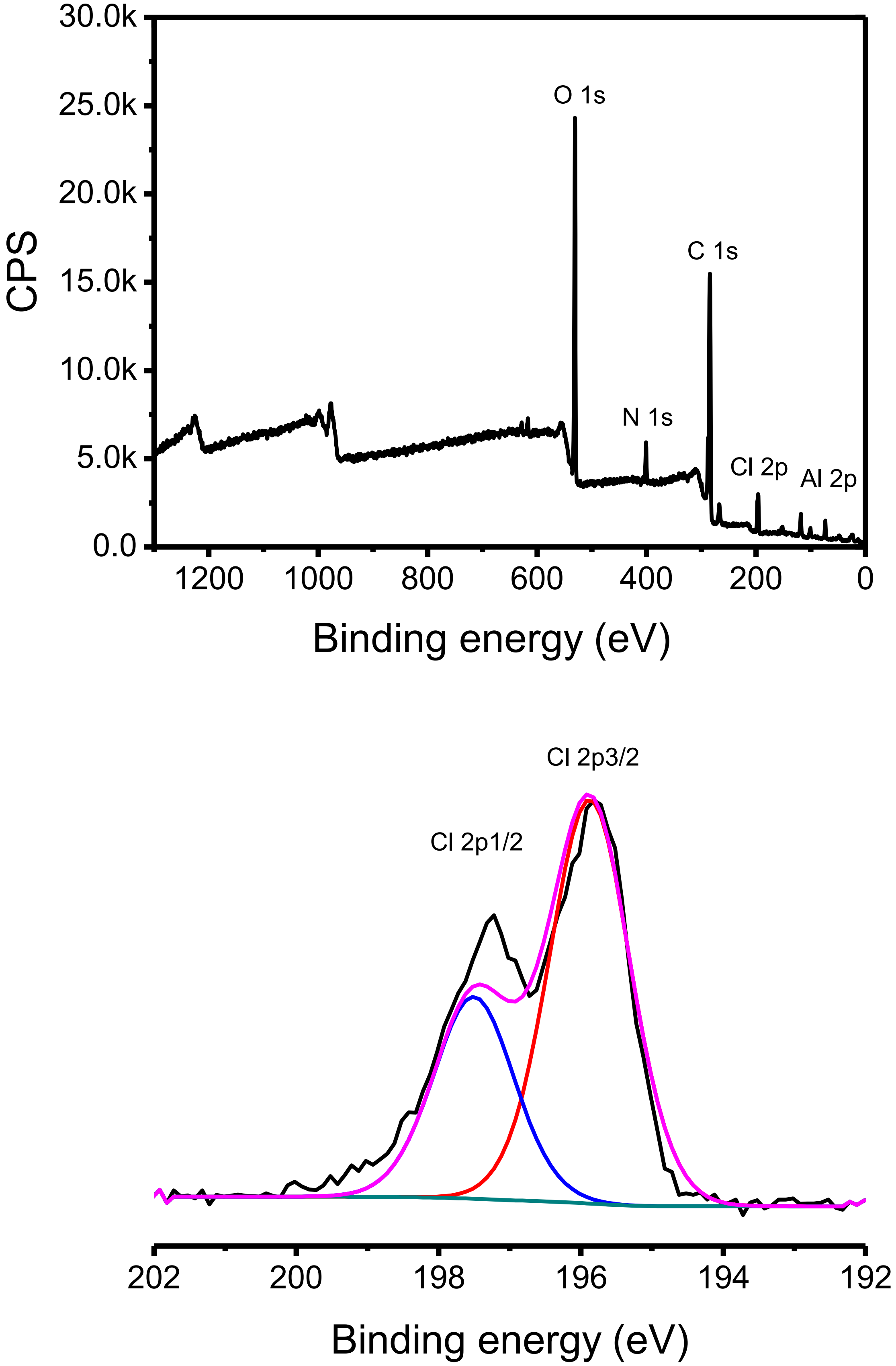

Figure S5. XPS spectra and fittings for MFM-305-CH_3.

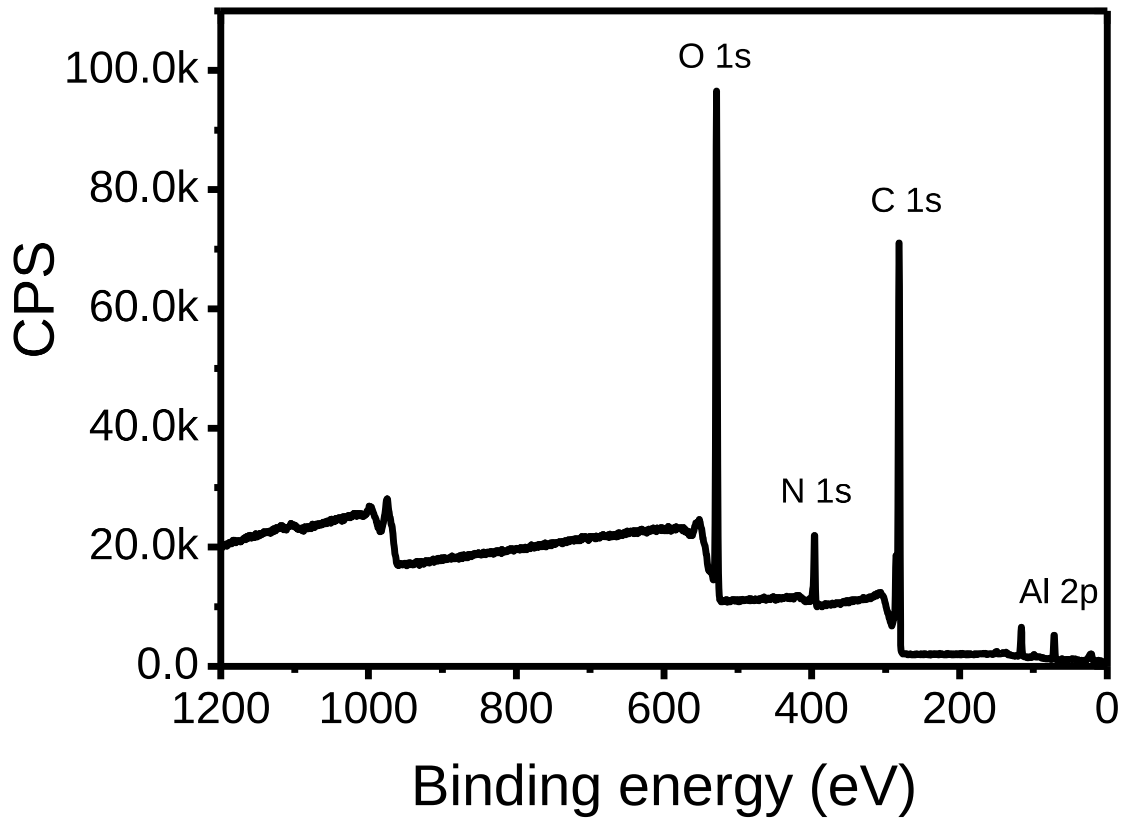

Figure S6. XPS spectra of MFM-305.

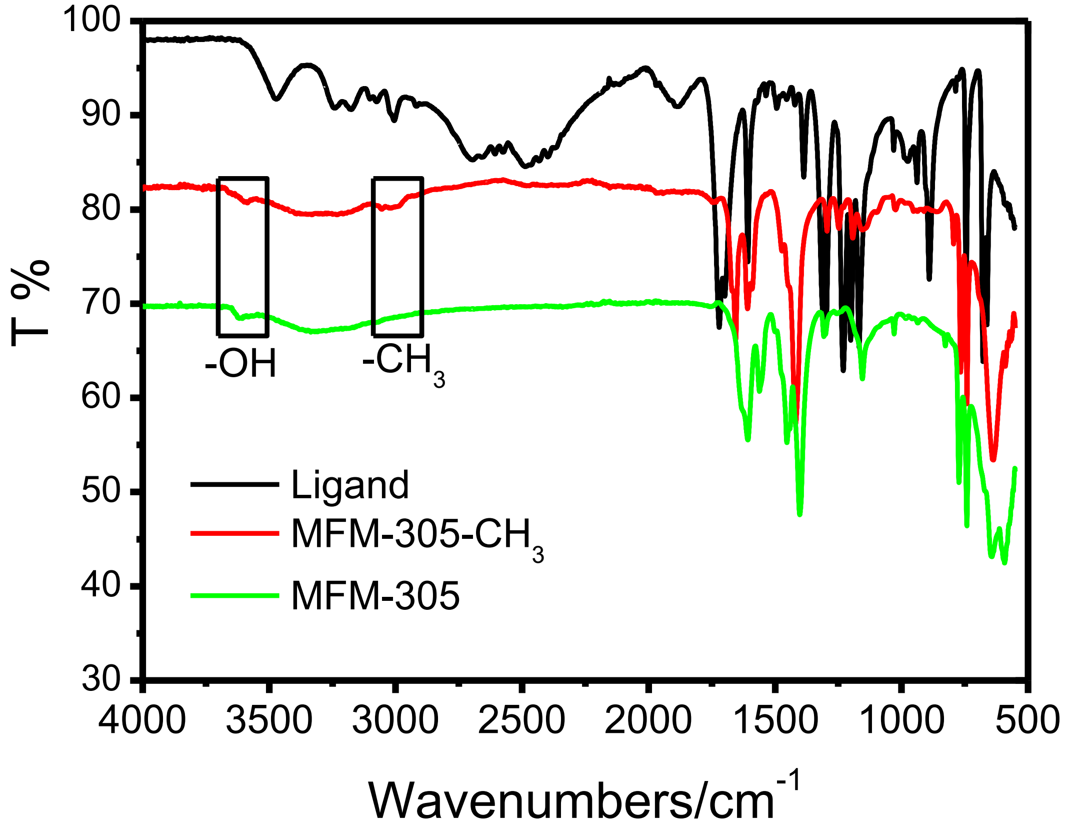

Figure S7. Infrared spectra of MFM-305-CH_3 and MFM-305.

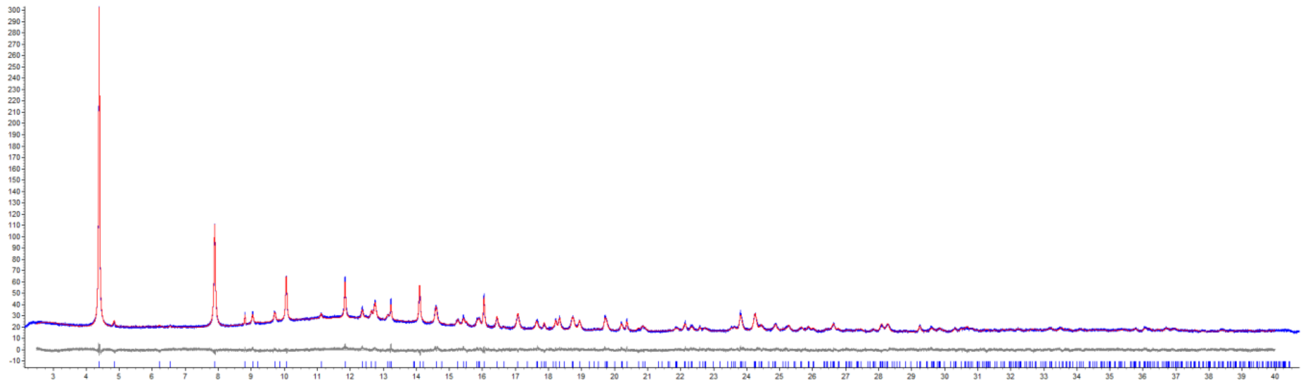

Figure S8. Synchrotron powder X-ray diffraction pattern and Rietveld refinement for MFM-305-CH_3.

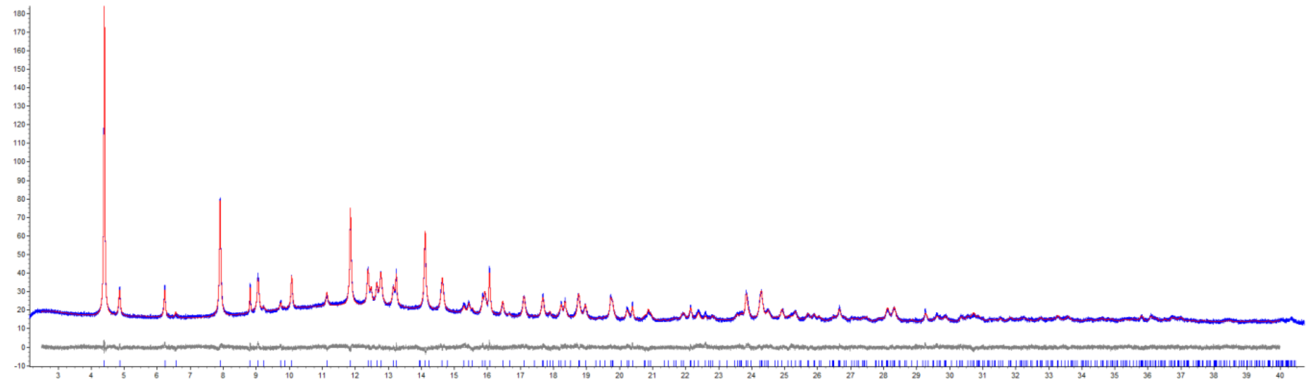

Figure S9. Synchrotron powder X-ray diffraction pattern and Rietveld refinement for CO_2@MFM-305-CH_3 at 273K.

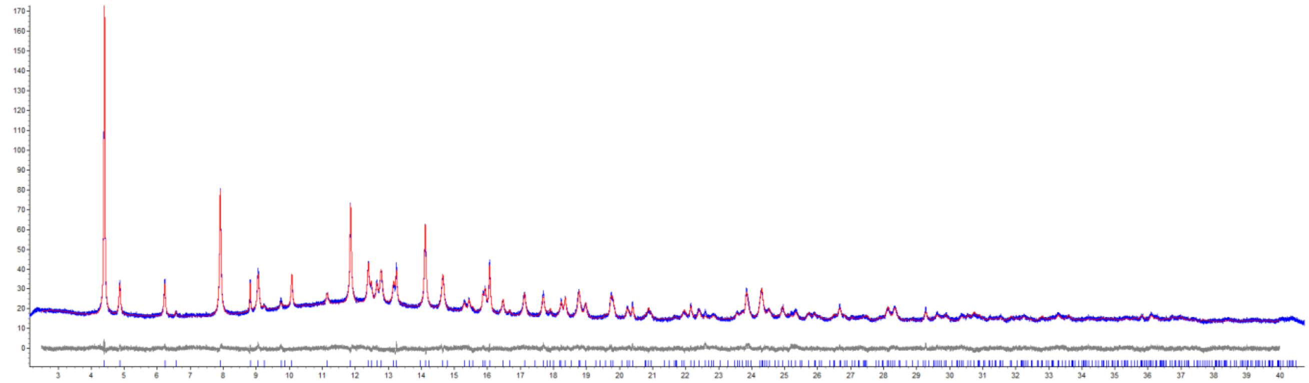

Figure S10. Synchrotron powder X-ray diffraction pattern and Rietveld refinement for CO_2@MFM-305-CH_3 at 230K.

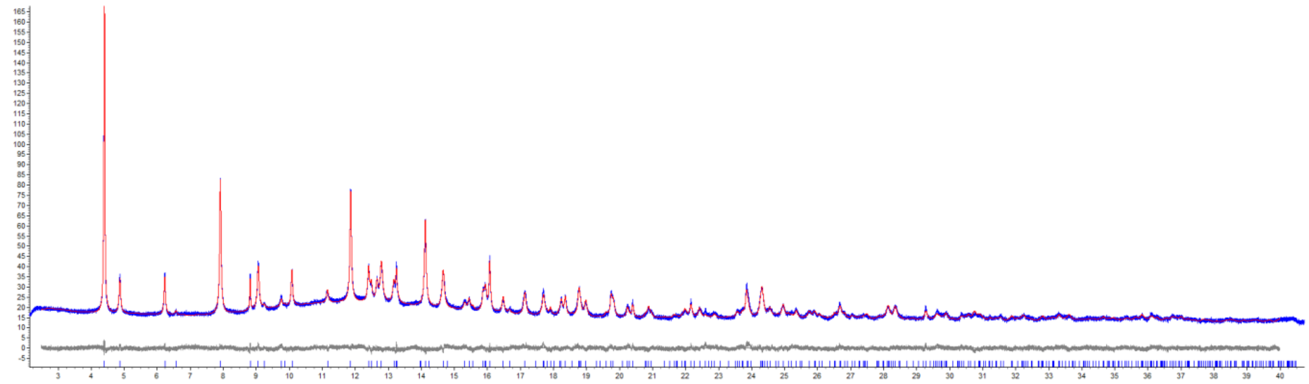

Figure S11. Synchrotron powder X-ray diffraction pattern and Rietveld refinement for CO_2@MFM-305-CH_3 at 198K.

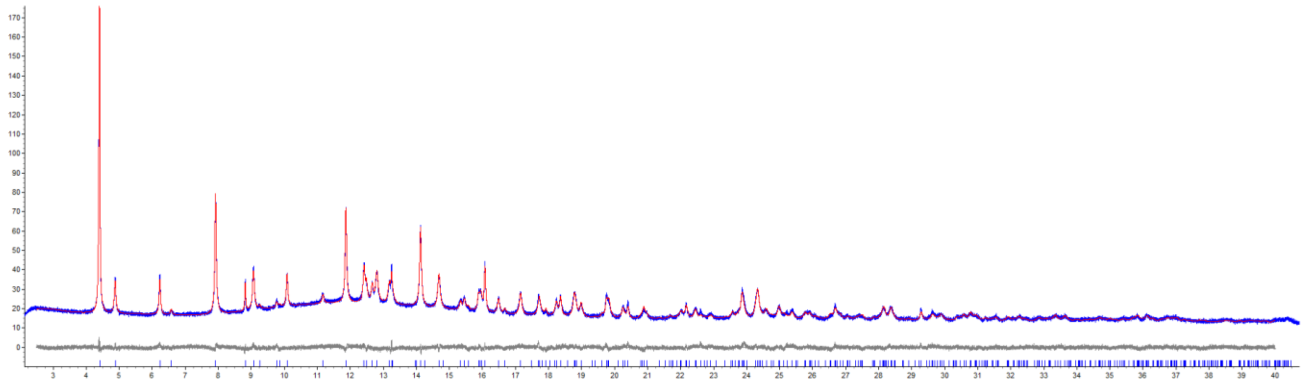

Figure S12. Synchrotron powder X-ray diffraction pattern and Rietveld refinement for CO_2@MFM-305-CH_3 at 150K.

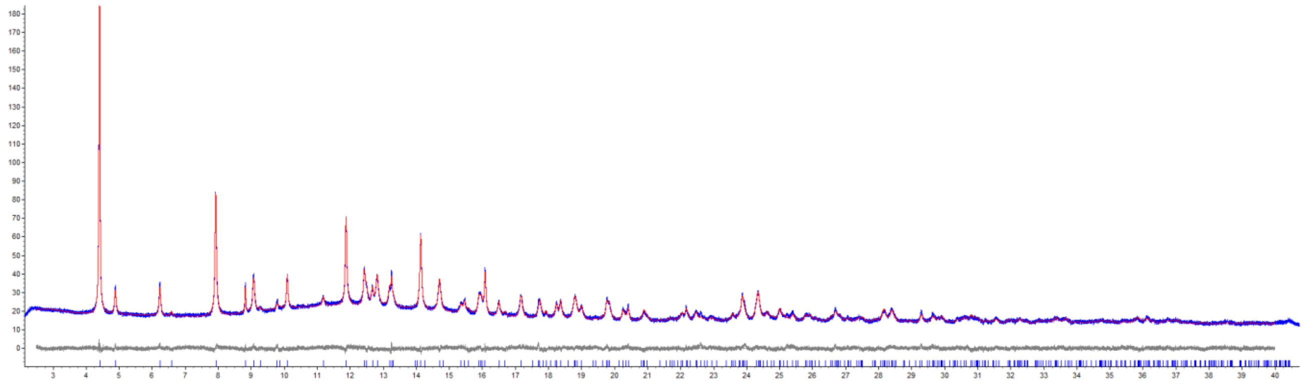

Figure S13. Synchrotron powder X-ray diffraction pattern and Rietveld refinement for CO_2@MFM-305-CH_3 at 117K.

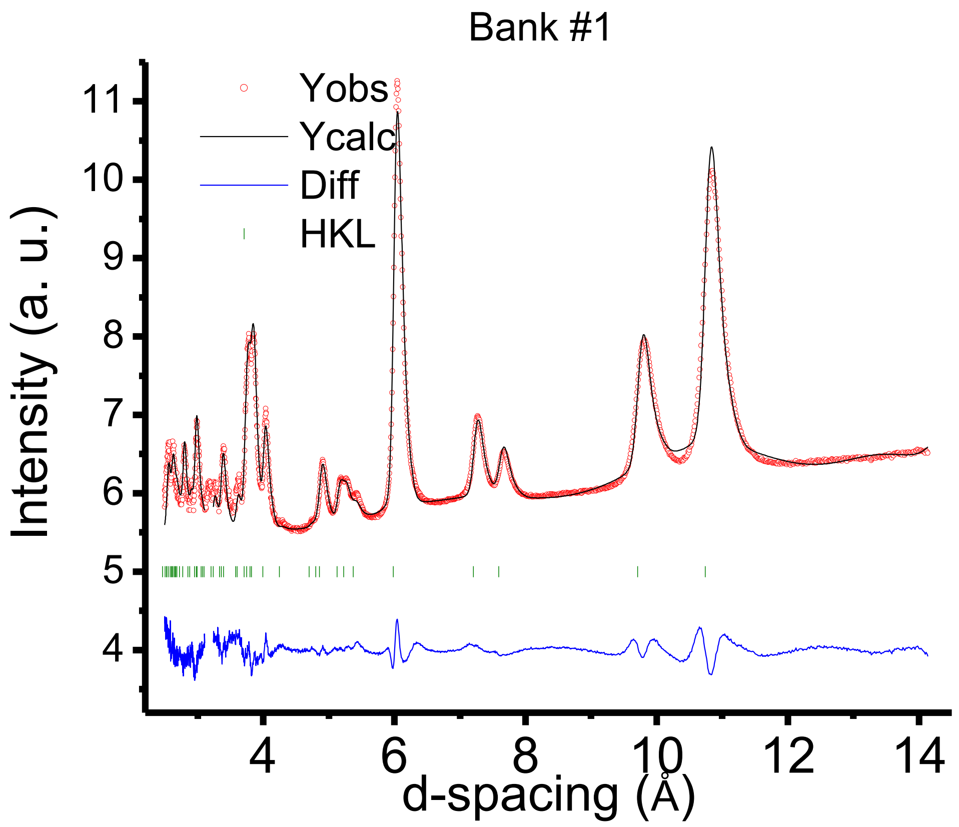
Bank #1
Yobs
Ycalc
Diff
HKL
Intensity (a. u.)
11
10
9
8
7
6
5
4
4
6
8
10
12
14
d-spacing (Å)

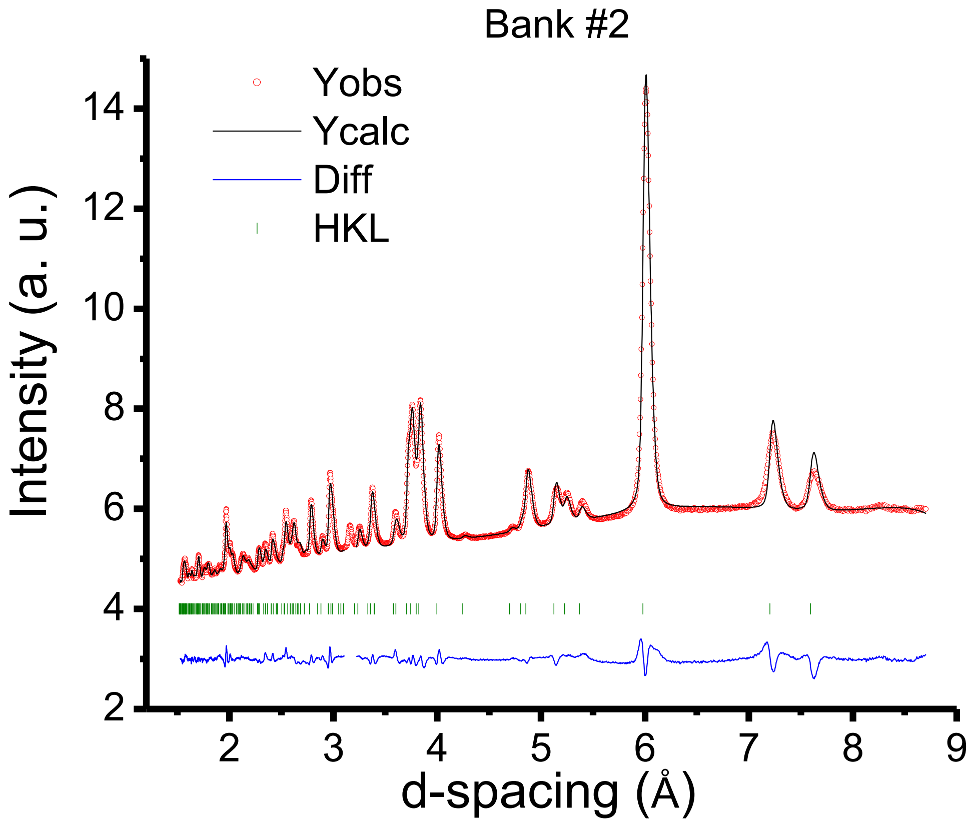
Bank #2
Yobs
Ycalc
Diff
HKL
Intensity (a. u.)
14
12
10
8
6
4
2
2
3
4
5
6
7
8
9
d-spacing (Å)

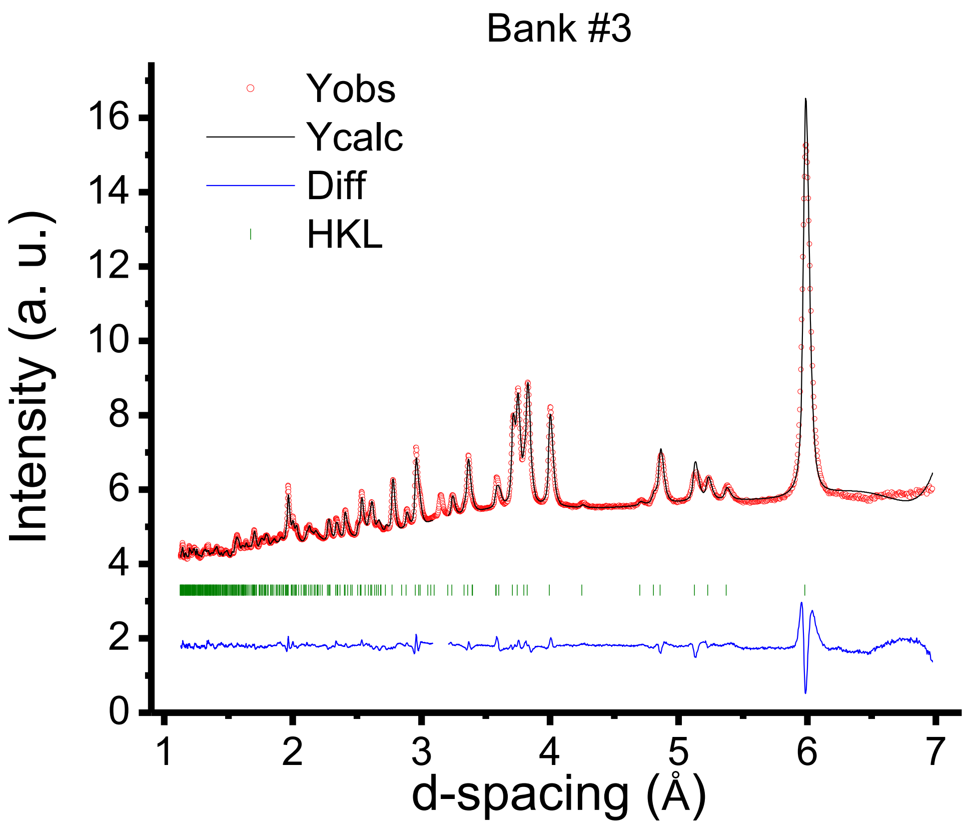

Bank #3
Yobs
Ycalc
Diff
HKL
Intensity (a. u.)
d-spacing (Å)
0
2
4
6
8
10
12
14
16
1
2
3
4
5
6
7

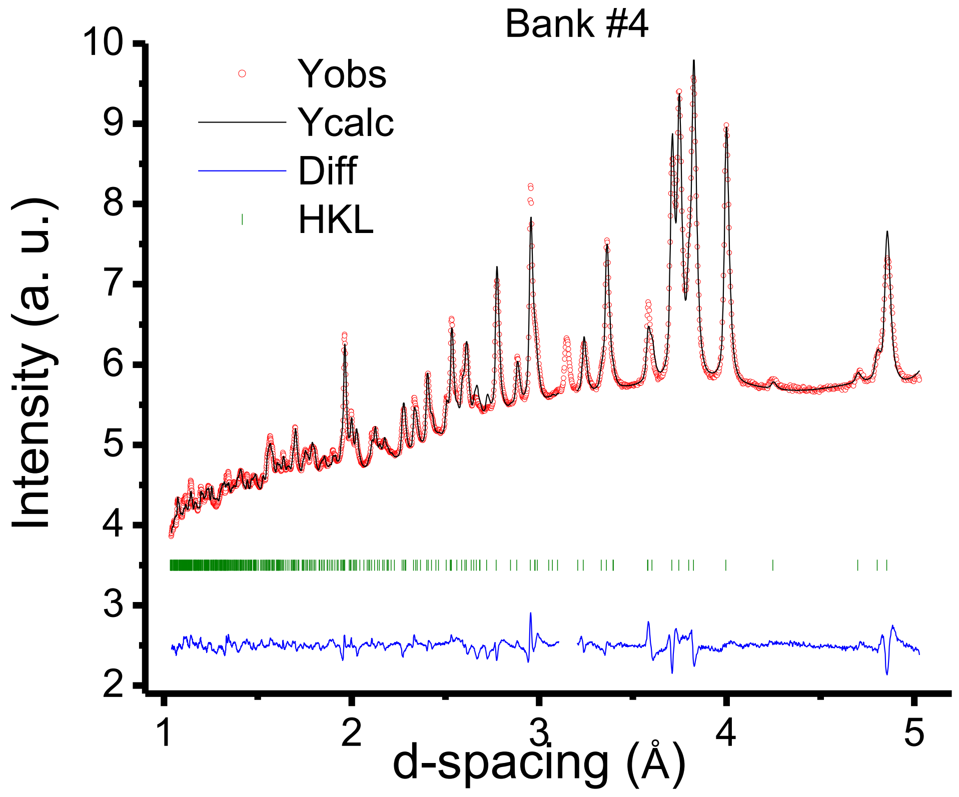

Bank #4
Yobs
Ycalc
Diff
HKL
Intensity (a. u.)
d-spacing (Å)
2
3
4
5
6
7
8
9
10
1
2
3
4
5

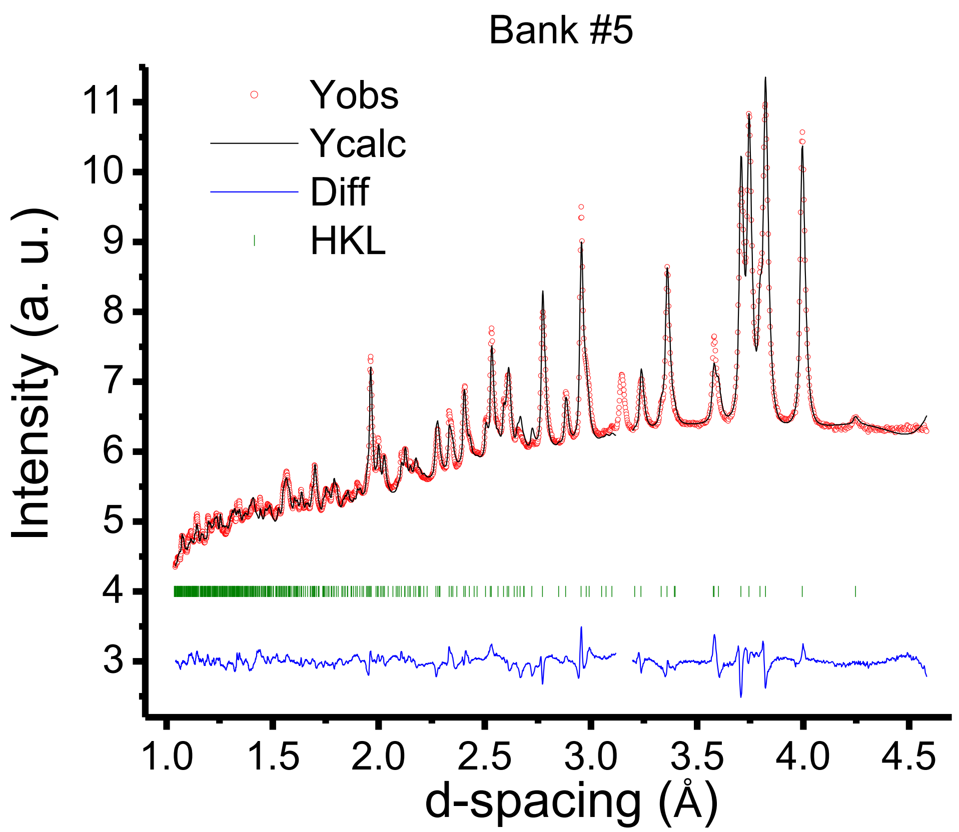

Figure S14. Neutron diffraction pattern and Rietveld refinement for CO_2@MFM-305-CH_3 at 7K (banks 1 to 5).

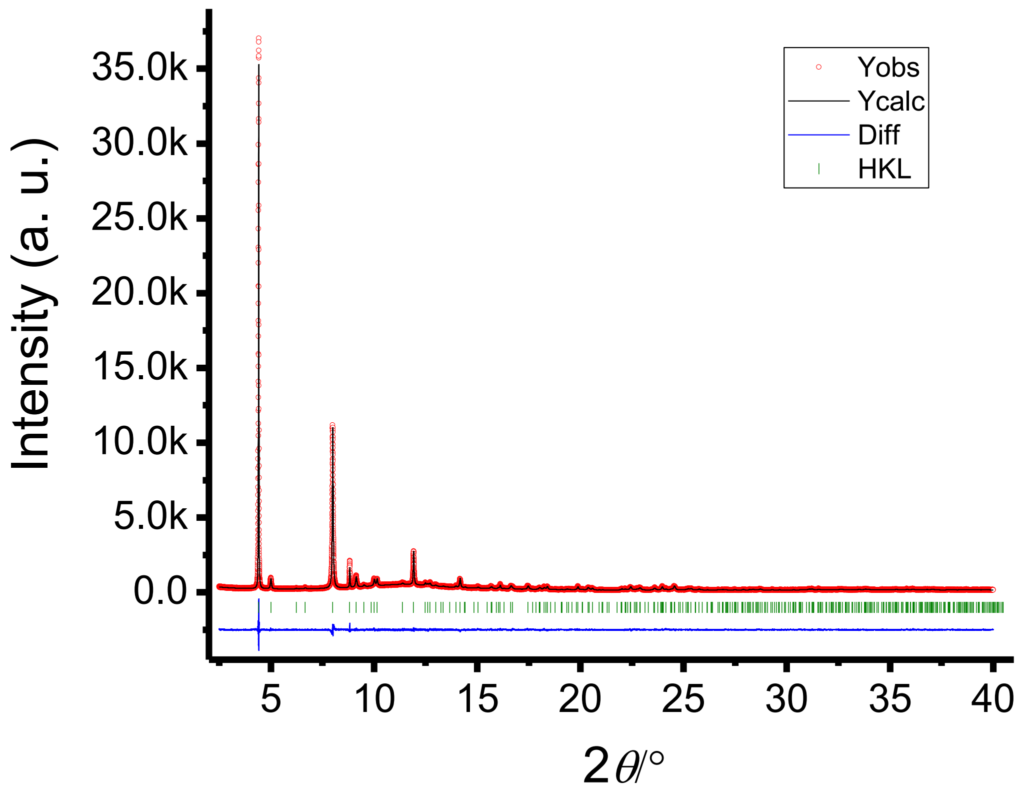

Figure S15. Synchrotron powder X-ray diffraction pattern and Rietveld refinement for MFM-305.

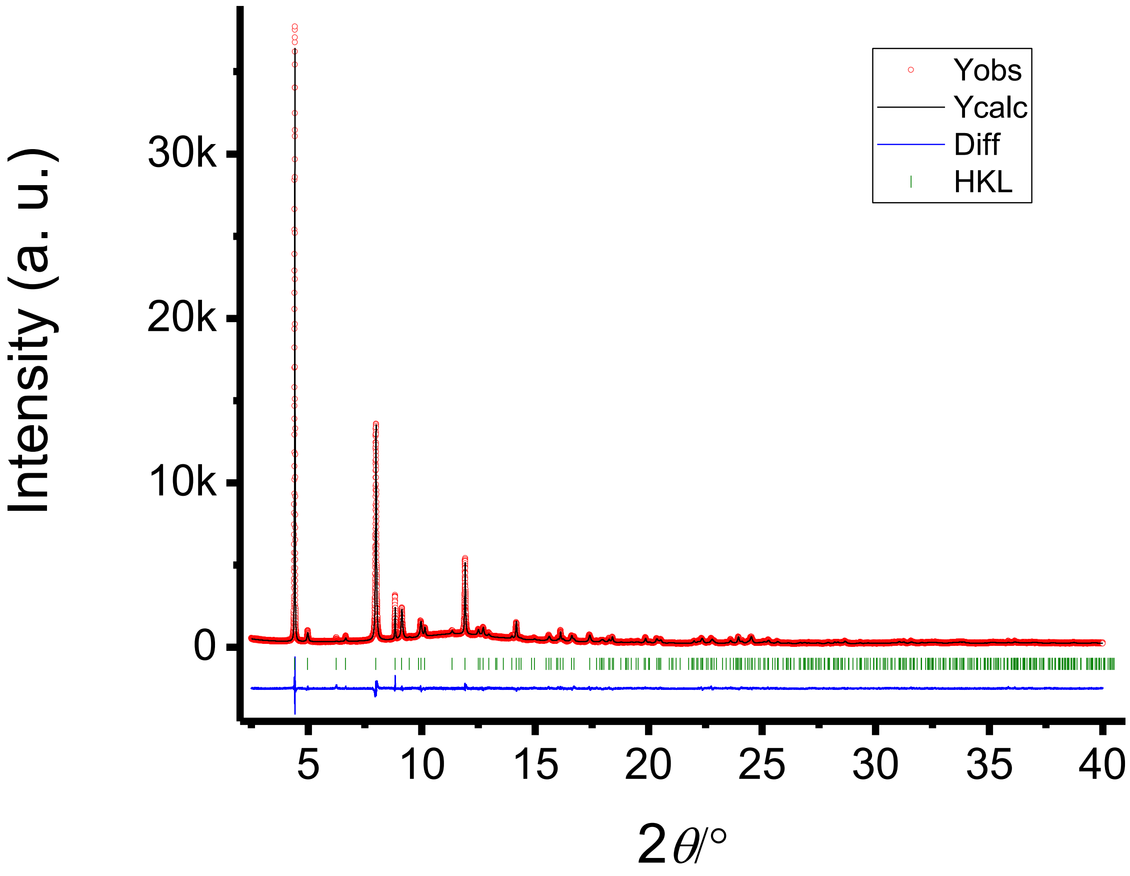

Figure S16. Synchrotron powder X-ray diffraction pattern and Rietveld refinement for CO_2@MFM-305 at 270K.

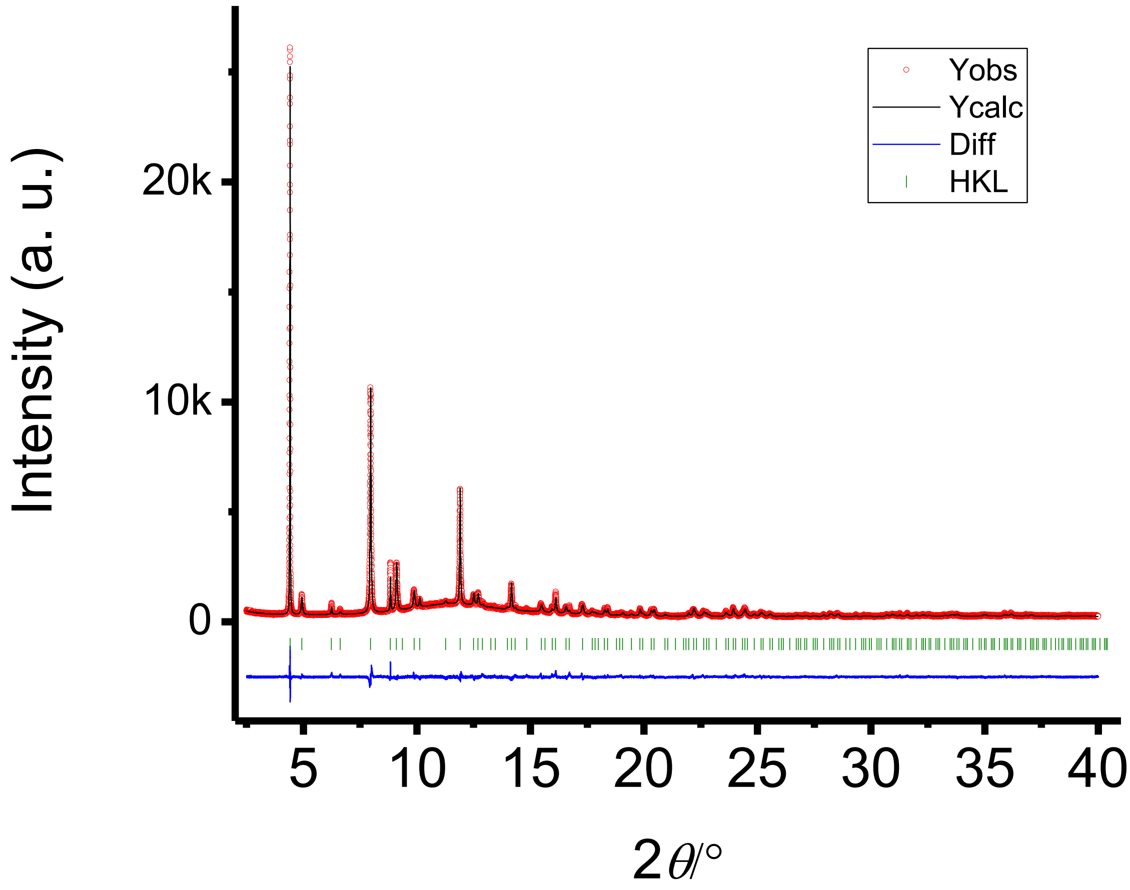

Figure S17. Synchrotron powder X-ray diffraction pattern and Rietveld refinement for CO_2@MFM-305 at 230K.

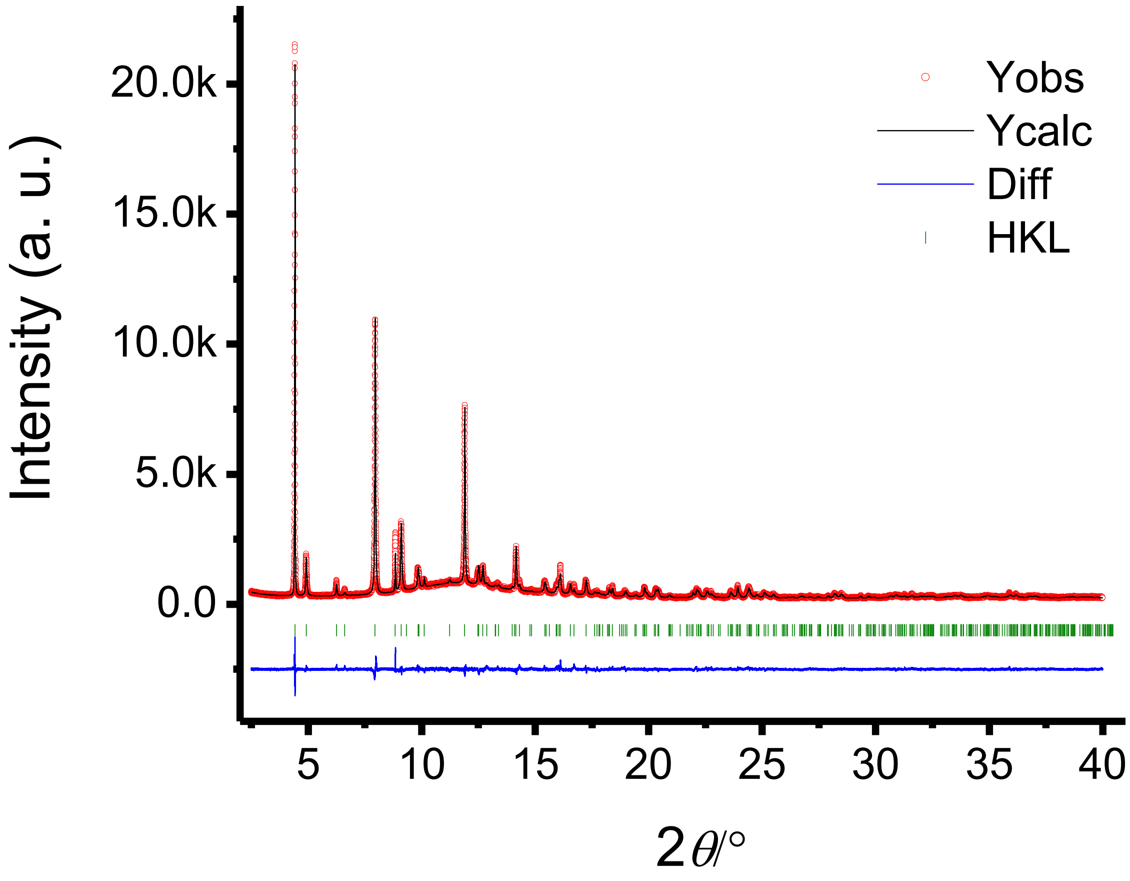

Figure S18. Synchrotron powder X-ray diffraction pattern and Rietveld refinement for CO_2@MFM-305 at 198K.

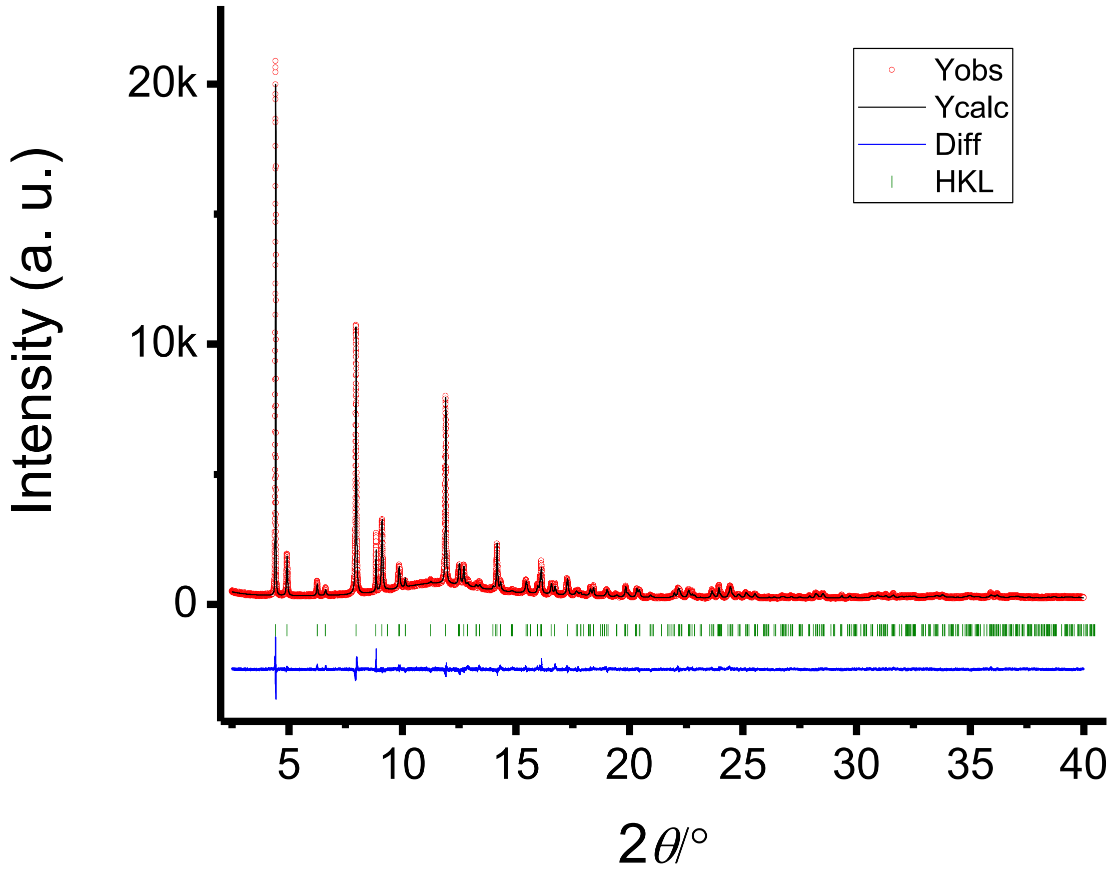

Figure S19. Synchrotron powder X-ray diffraction pattern and Rietveld refinement for CO_2@MFM-305 at 150K.

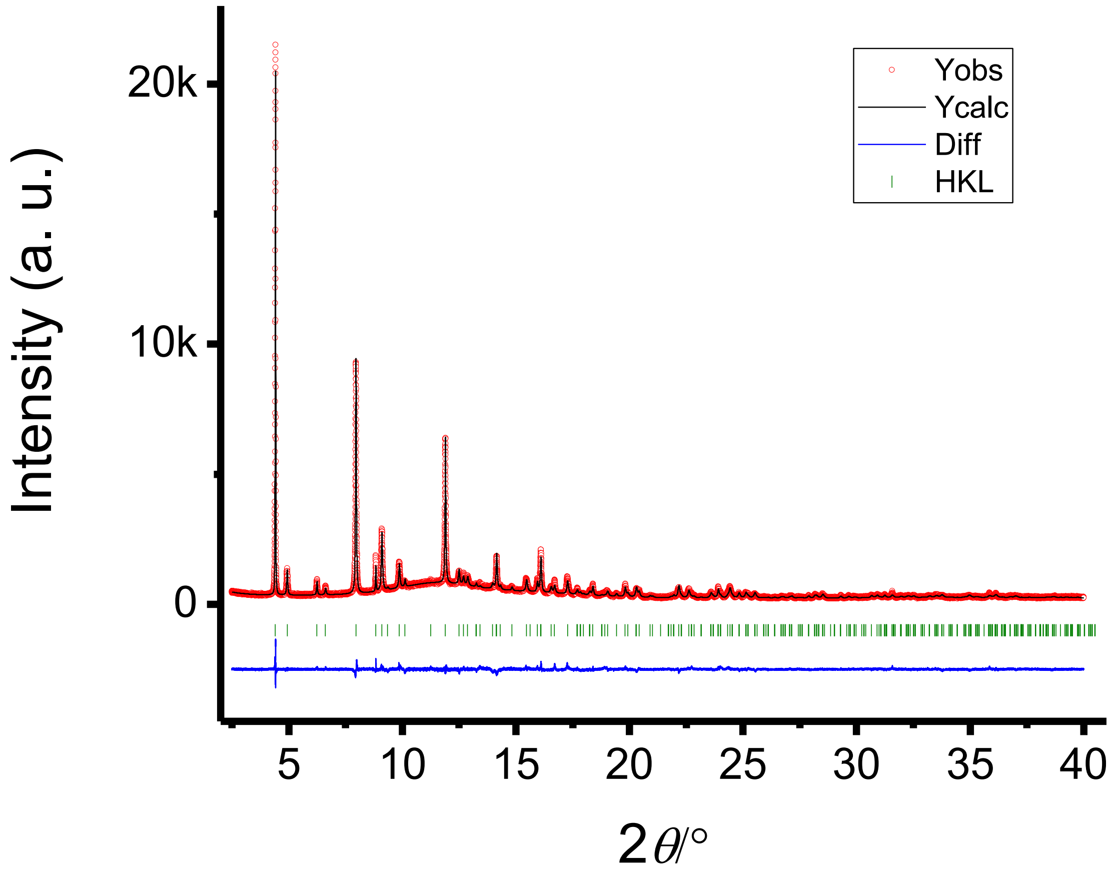

Figure S20. Synchrotron powder X-ray diffraction pattern and Rietveld refinement for CO_2@MFM-305 at 100K.

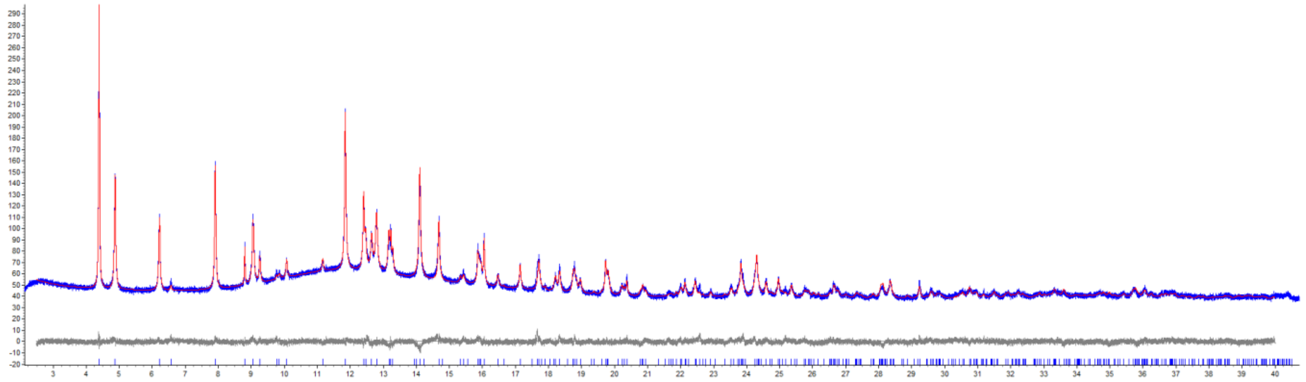

Figure S21. Synchrotron powder X-ray diffraction pattern and Rietveld refinement for SO_2@MFM-305-CH_3 at 298K.

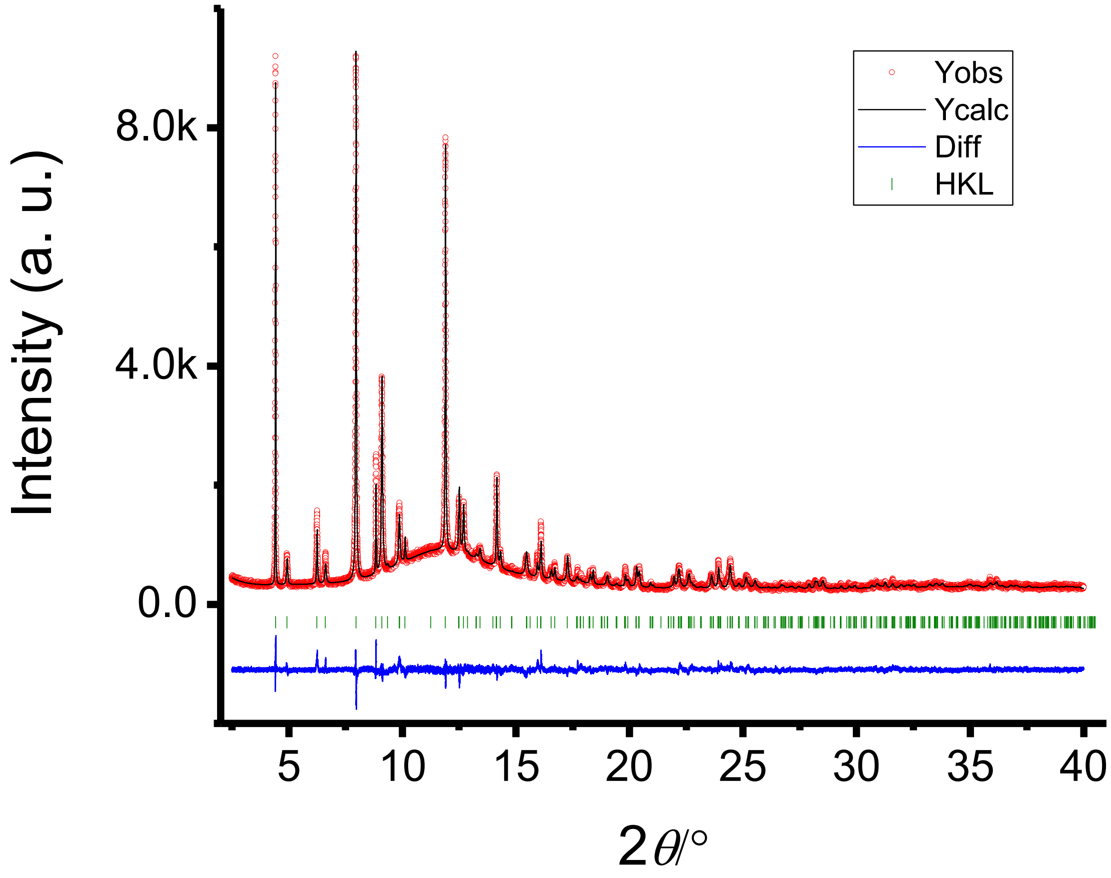

Figure S22. Synchrotron powder X-ray diffraction pattern and Rietveld refinement for SO_2@MFM-305at 298K.

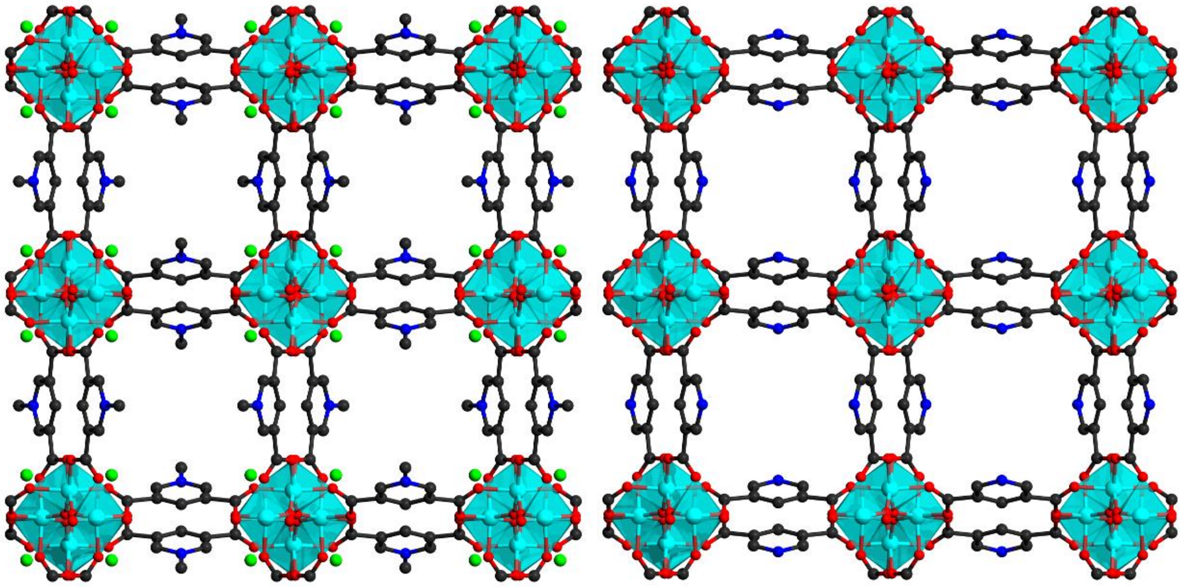

Figure S23. Structure of MFM-305-CH_3 (left) and MFM-305 (right). The structure was solved from high-resolution PXRD data. View of the three-dimensional framework structure with a channel formed along the *c*-axis. (aluminium, turquoise, carbon, grey, oxygen, red, chlorine, bright green).

Table S1. The adsorption capacities of N_2, CO_2, SO_2 in MFM-305-CH_3 and MFM-305 at 1.0 bar.

		MFM-305-CH_3/mmol g^{-1}	MFM-305/mmol g^{-1}
N_2	77 K	5.20	11.93
	298 K	0.33	0.23
CO_2	195 K	4.23	5.69
	273 K	2.41	3.02
	298 K	2.67	3.55
SO_2	273 K	5.28	9.05
	298 K	5.16	6.99

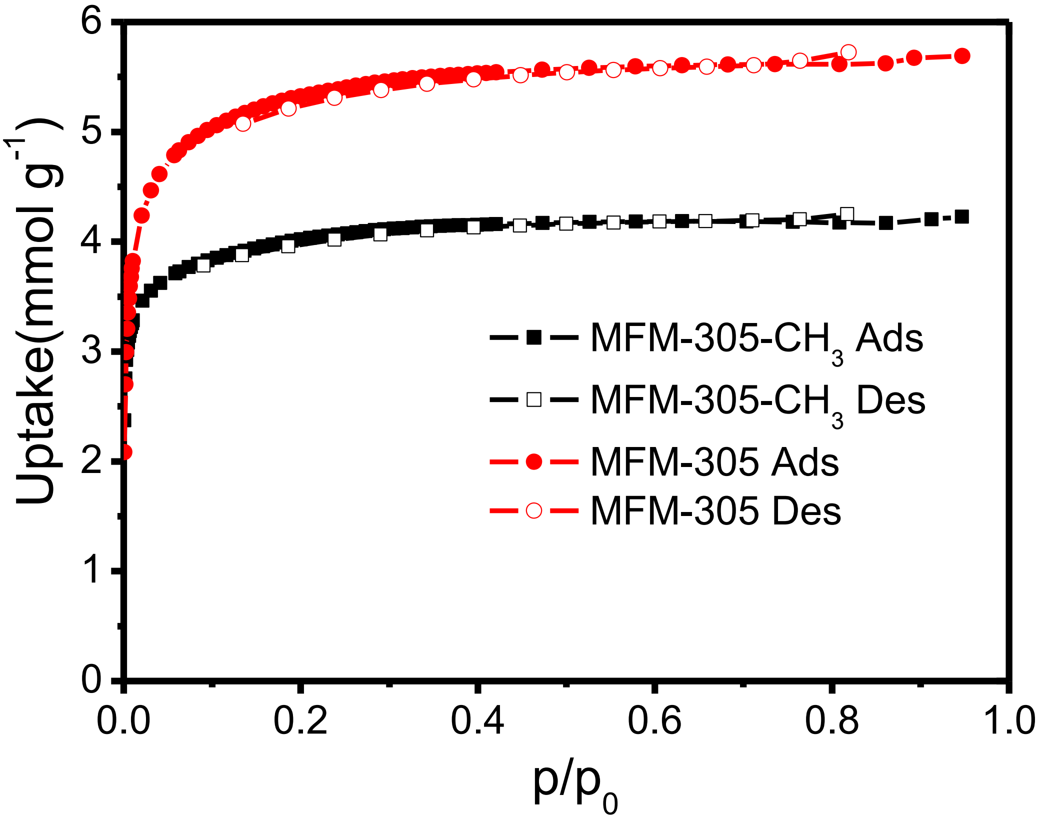

Figure S24. Adsorption isotherm (195K) of CO_2 in MFM-305-CH_3 and MFM-305.

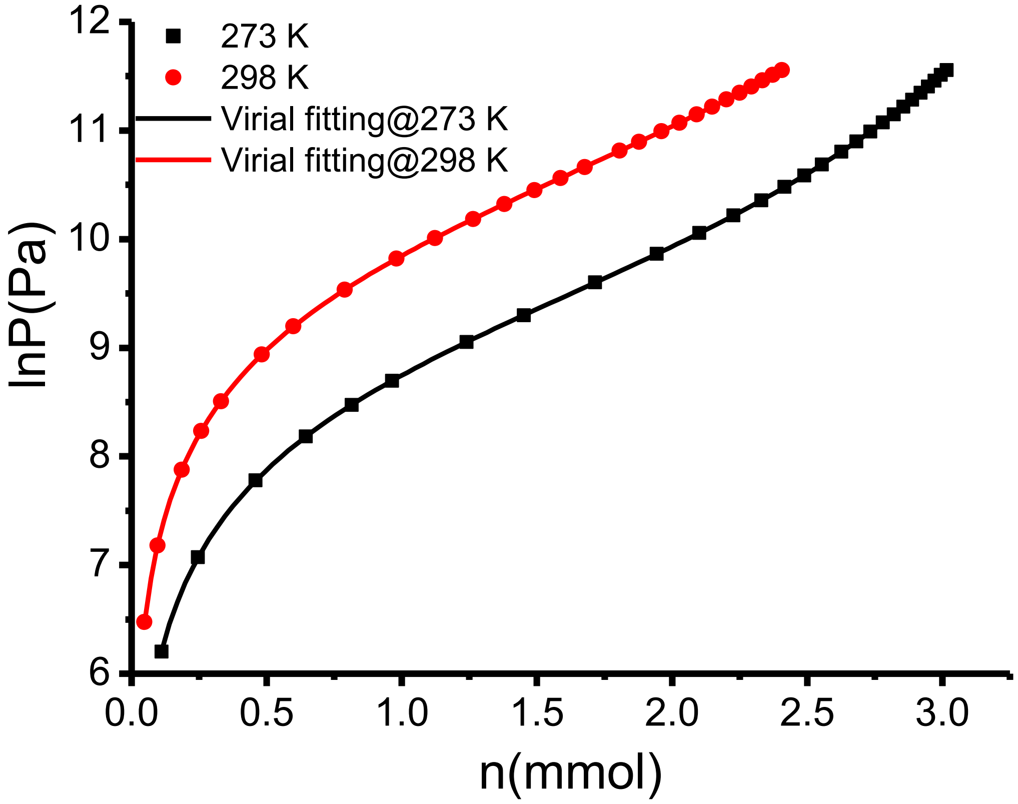

Figure S25. The virial fitting of CO_2 sorption data for MFM-305-CH_3.

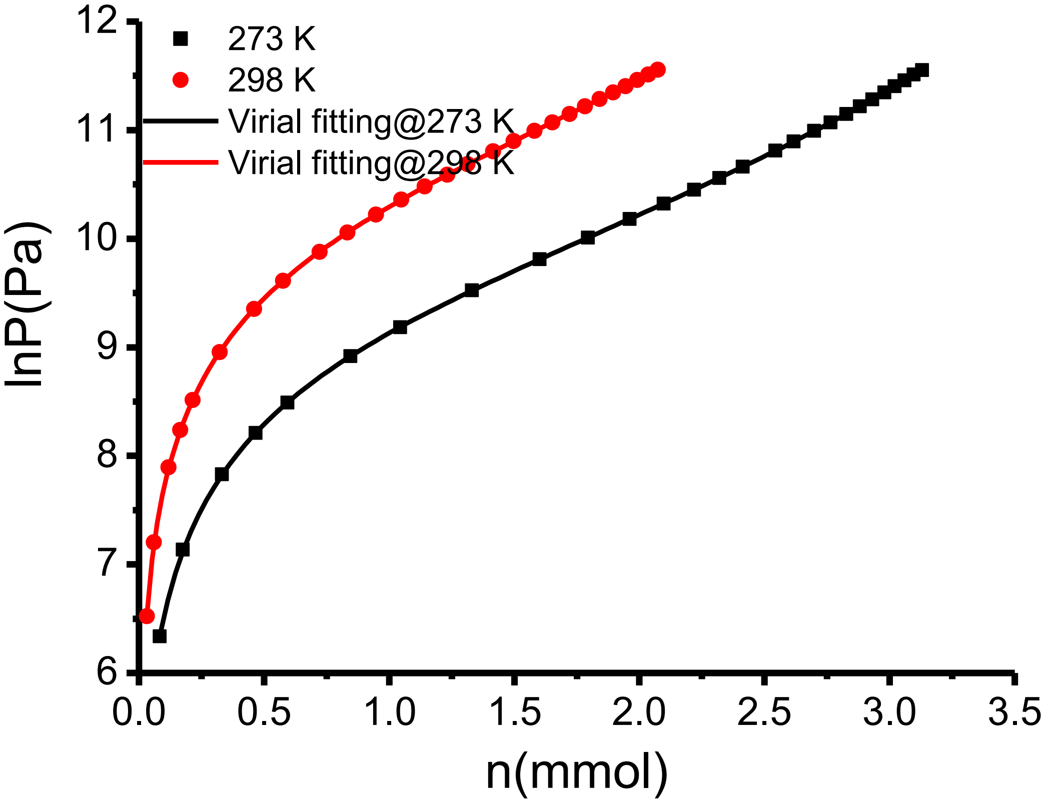

Figure S26. The virial fitting of CO_2 sorption data for MFM-305.

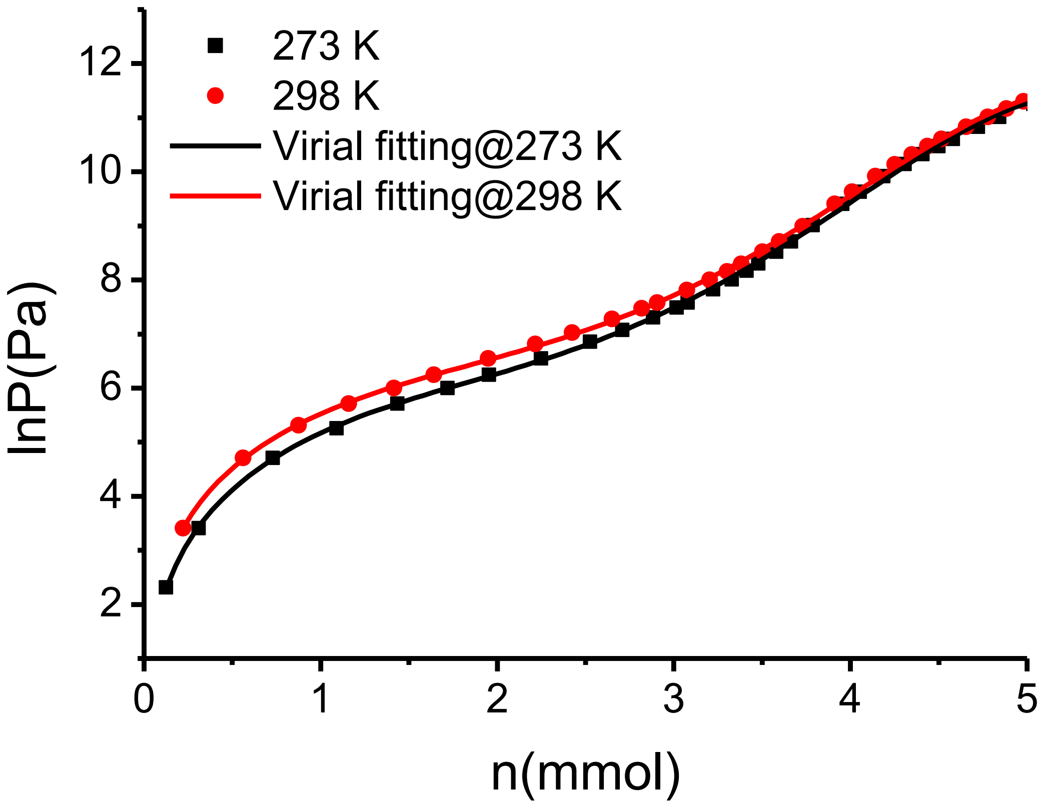

Figure S27. The virial fitting of SO_2 sorption data for MFM-305-CH_3.

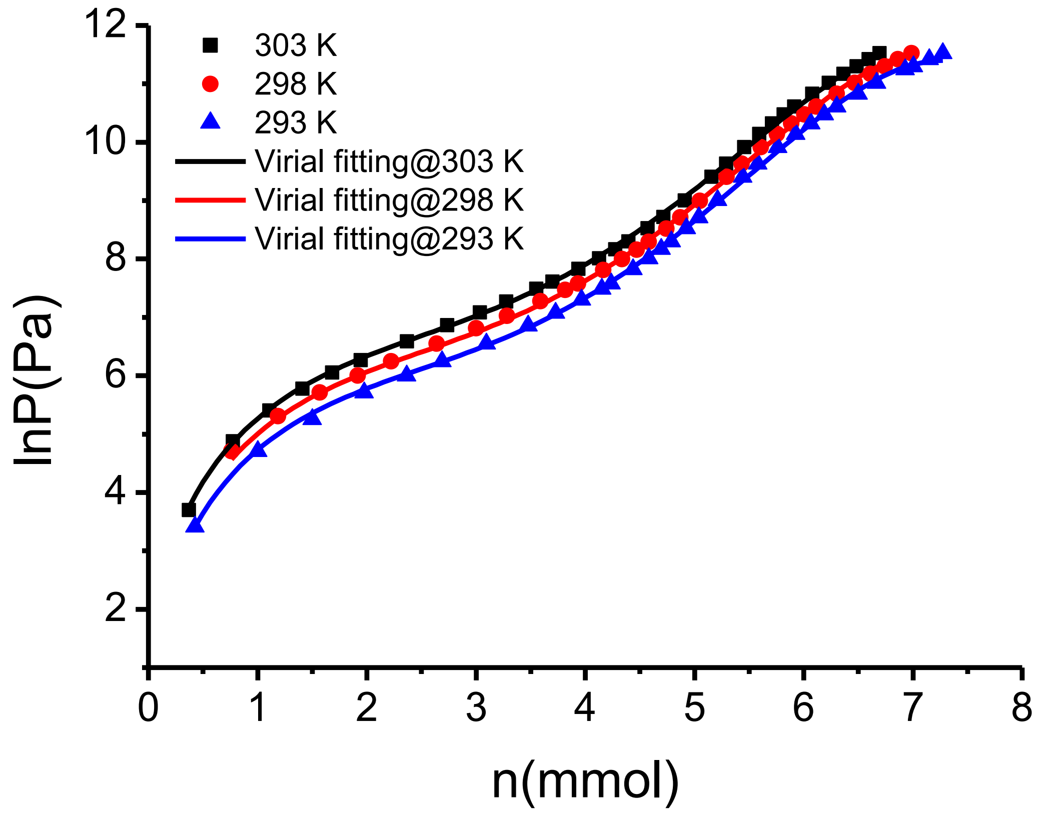

Figure S28. The virial fitting of SO_2 sorption data for MFM-305.

Table S2. The adsorption capacities of SO_2 in various porous materials at 1bar and 298 K.

	Surface area /m^2 g^{-1}	Pore volume cm^{-3} g^{-1}	SO_2 uptake /mmol g^{-1}	SO_2 packing density/ g cm^{-3}	refs
MFM-305-CH_3	256	0.181	5.16	1.825	This work
FMOF-2	378	0.217	2.19	0.647	9
SIFSIX-3-Zn	250	0.188	1.89	0.644	10
SIFSIX-3-Ni	368	0.191	2.74	0.919	10
Mg-MOF-74	1525	0.62	8.6	0.888	11
MFM-305	779	0.372	6.99	1.199	This work
ZnCo	700	0.356	1.8	0.3249	12
CoCo	712	0.658	2.5	0.243	12
SIFSIX-2-Cu-i	735	0.26	6.9	1.700	10
MFM-300(Al)	1037	0.375	7.1	1.213	13
MFM-300(In)	1071	0.419	8.28	1.266	14
SIFSIX-1-Cu	1337	0.683	11.01	1.033	10
IRMOF-3	1568	1.07	6	0.359	11
Ni(bdc)(ted)0.5	1783	0.665	9.97	0.961	15
Zn(bdc)(ted)0.5	1888	0.65	4.41	0.435	15
MOF-5	2205	1.216	1	0.053	11
MFM-202a	2220	0.953	10.2	0.686	16
SIFSIX-2-Cu	3140	1.15	6.5	0.362	10

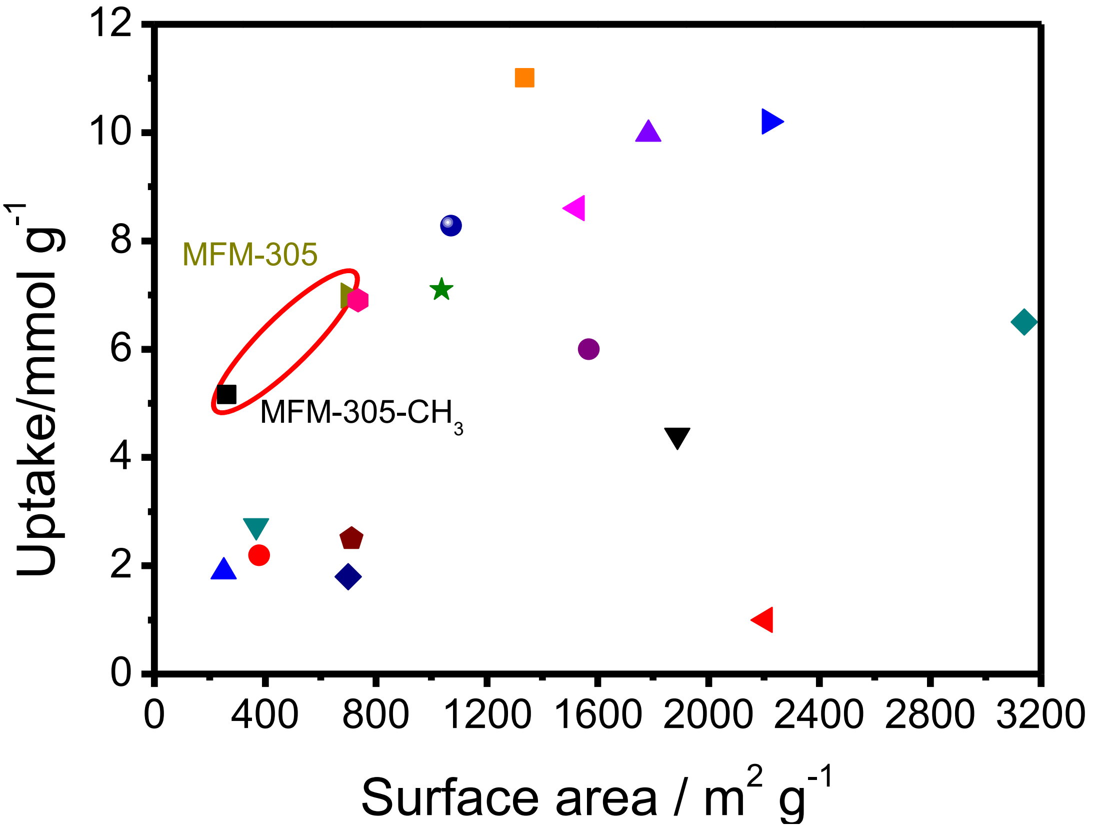

Figure S29. Comparison of the SO_2 uptake in reported MOFs (1 bar, 298K) with respect to surface area.

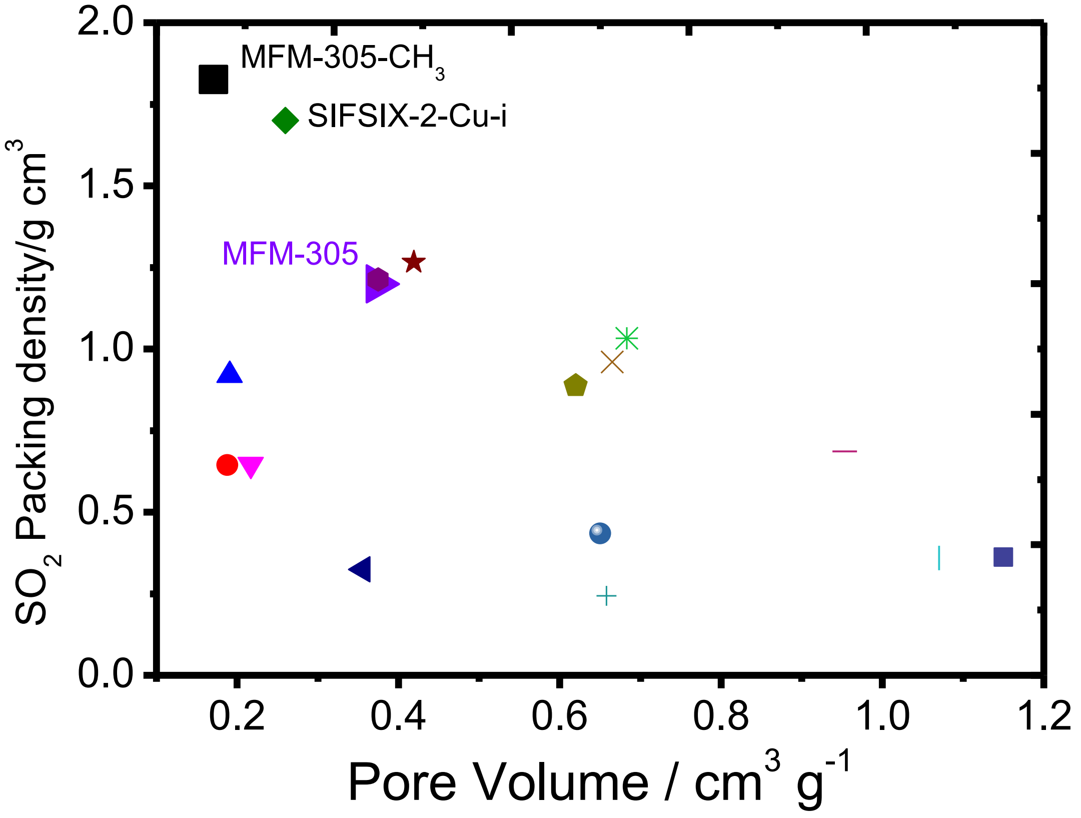

Figure S30. Comparison of the SO_2 packing density in reported MOFs (1 bar, 298K) with respect to pore volume.

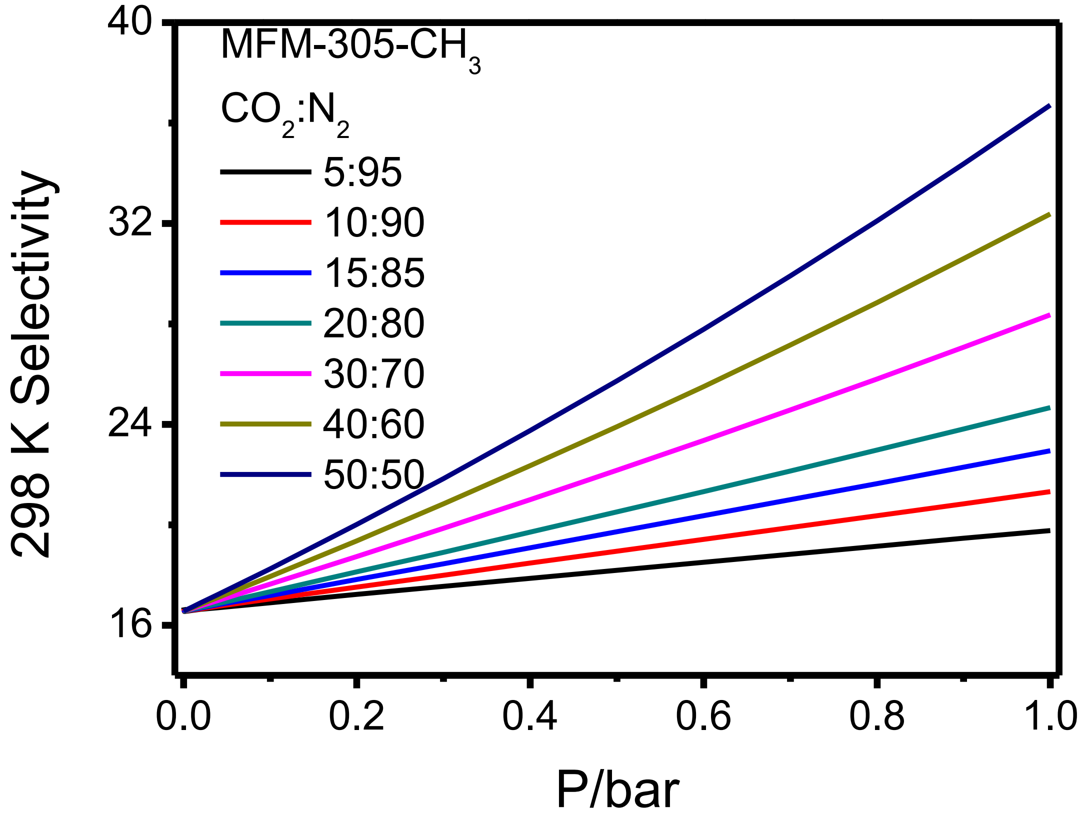

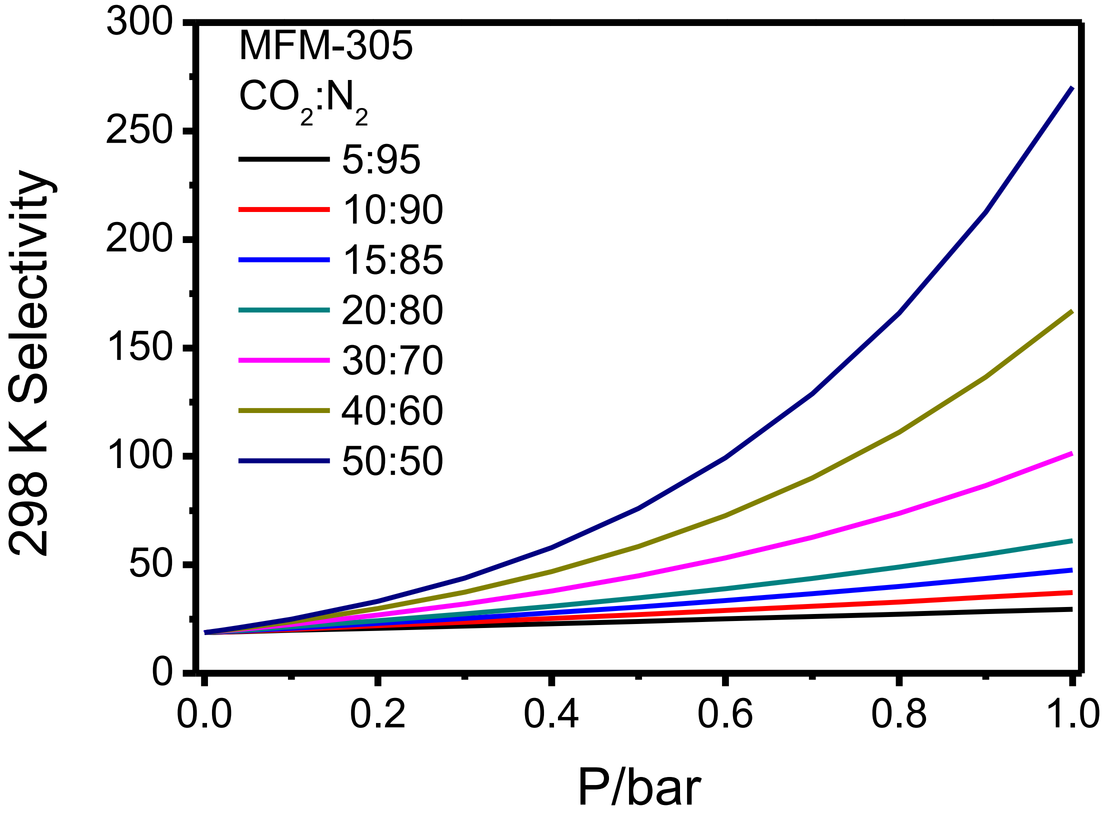

Figure S31. IAST selectivity for CO_2/N_2 mixtures in MFM-305-CH_3 (top) and MFM-305(bottom) up to a pressure of 1 bar at 298 K.

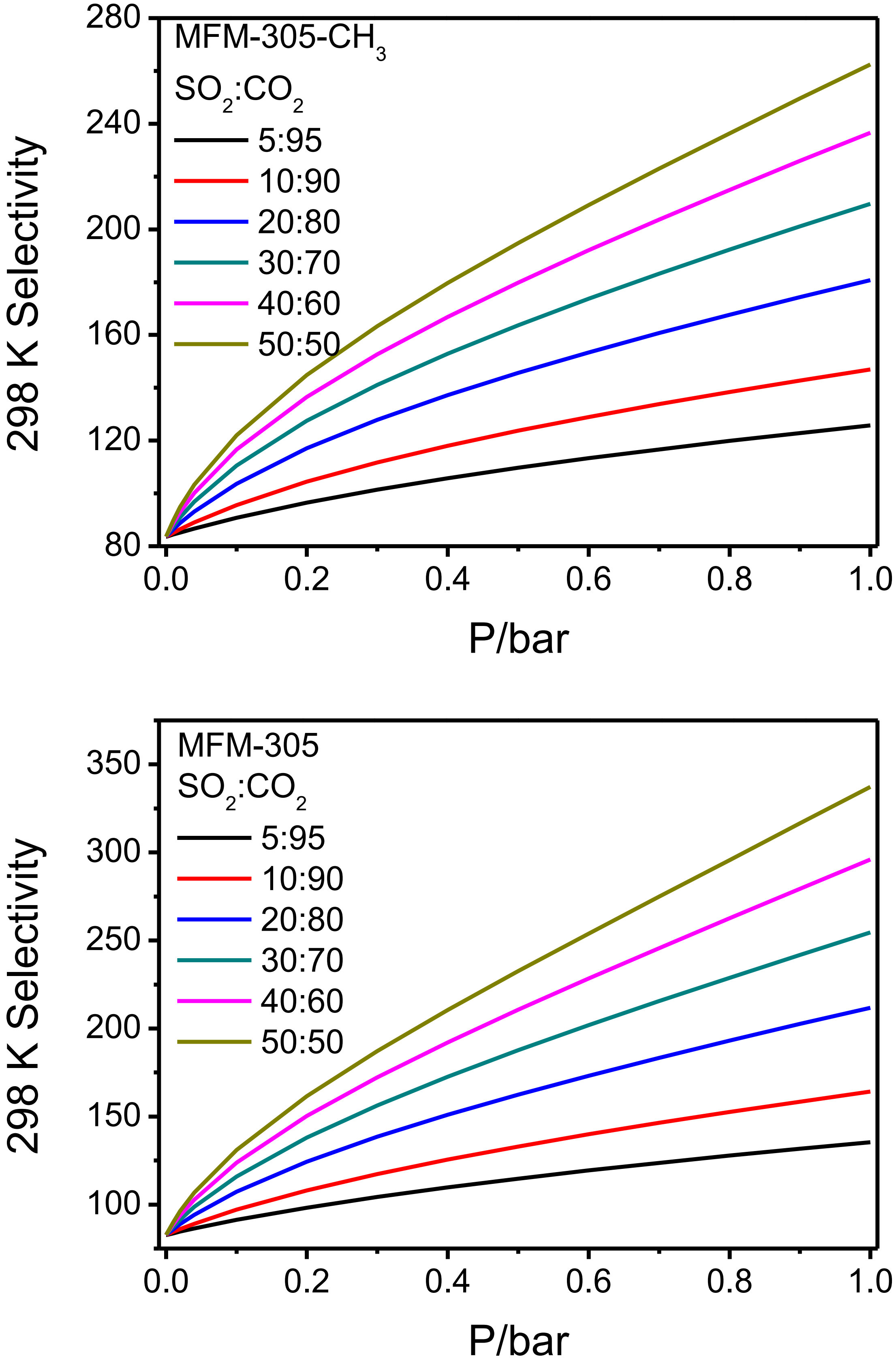

Figure S32. IAST selectivity for SO_2/CO_2 mixtures in MFM-305-CH_3 (up) and MFM-305 (bottom) up to a pressure of 1 bar at 298 K.

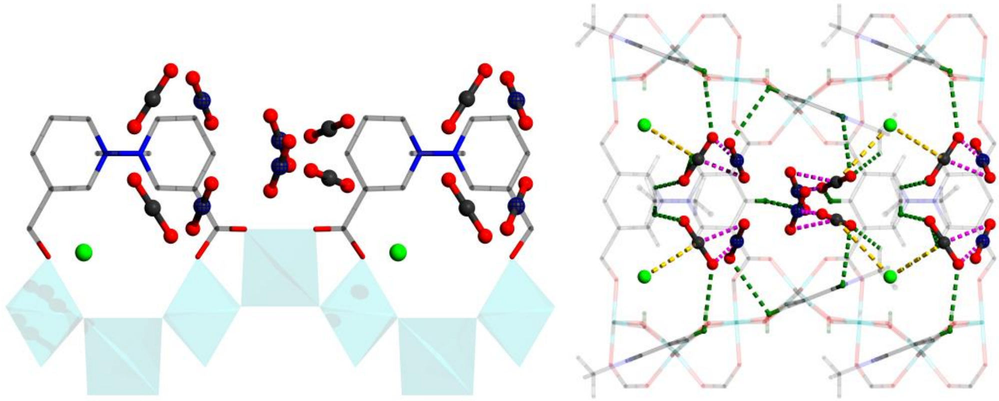

a.

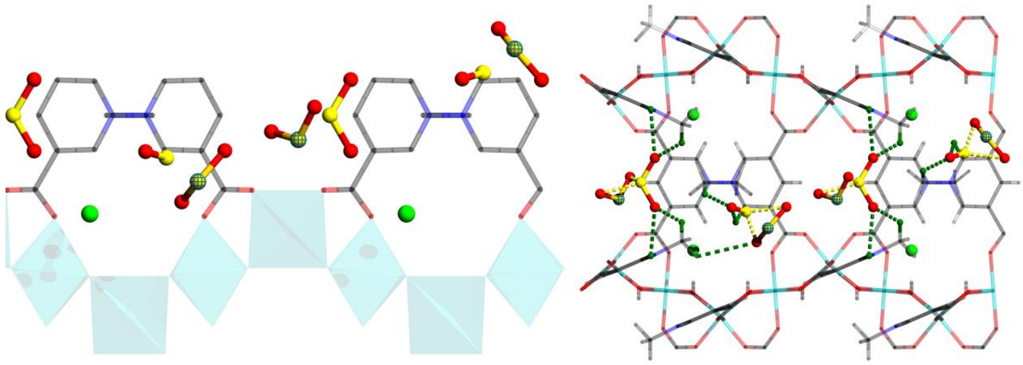

b.

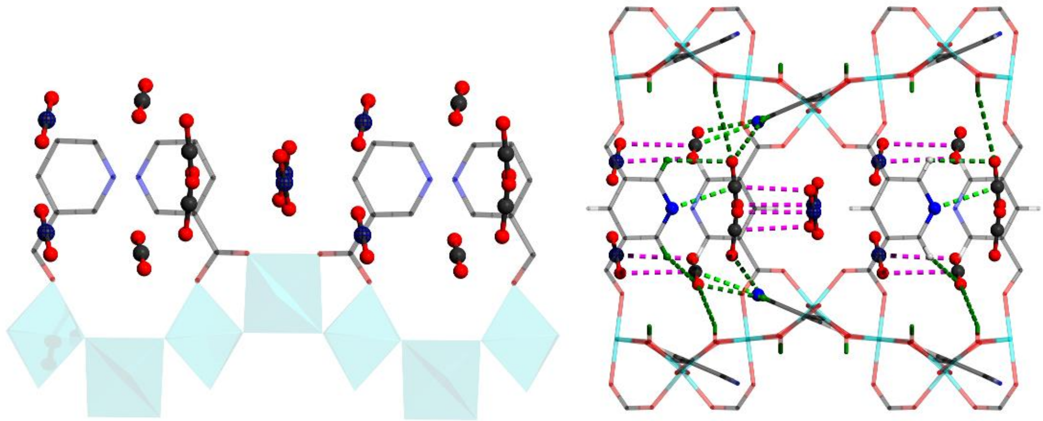

c.

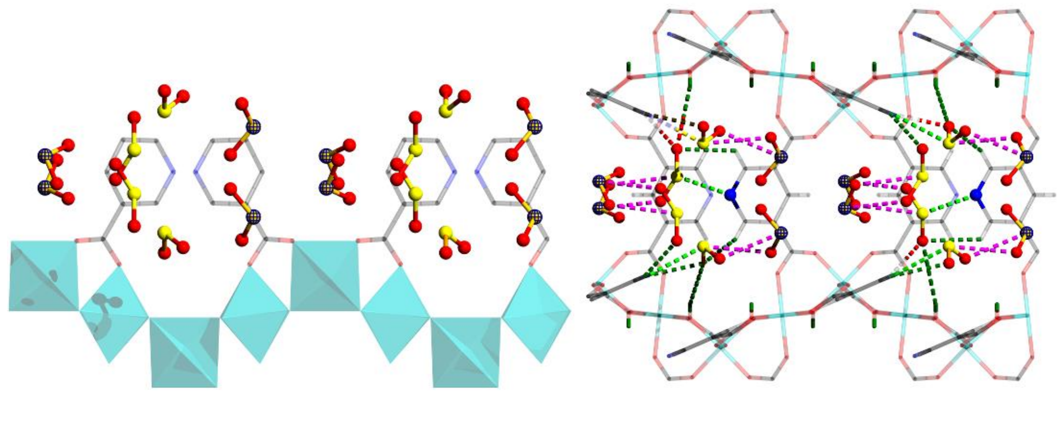

d.

Figure S33. Views of the packing and interaction of adsorbed CO_2 within (a) MFM-305-CH_3 and (c) MFM-305, and of adsorbed SO_2 within (b) MFM-305-CH_3 and (d) MFM-305.

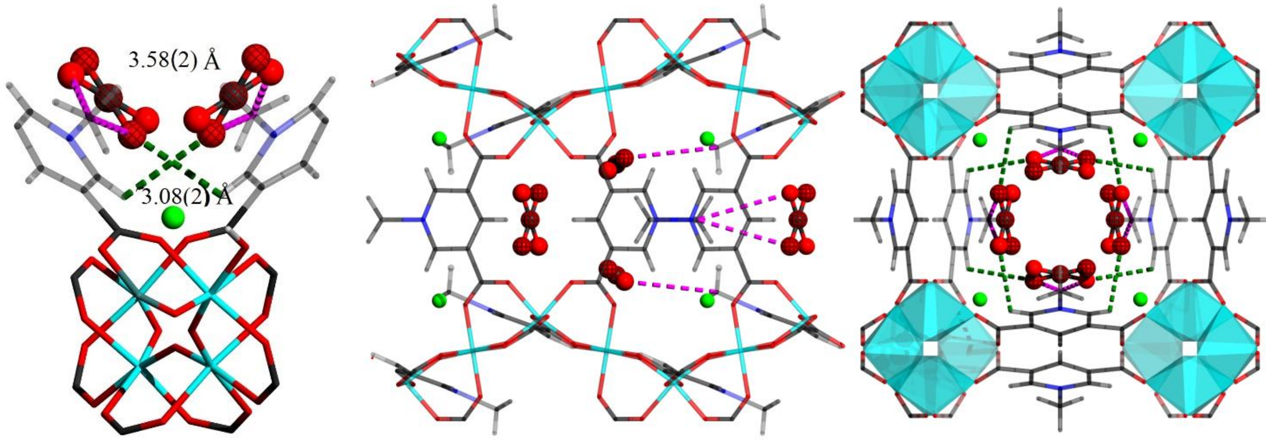

Figure S34. Views of the packing and interaction of adsorbed CO_2 within MFM-305-CH_3 at 7K.

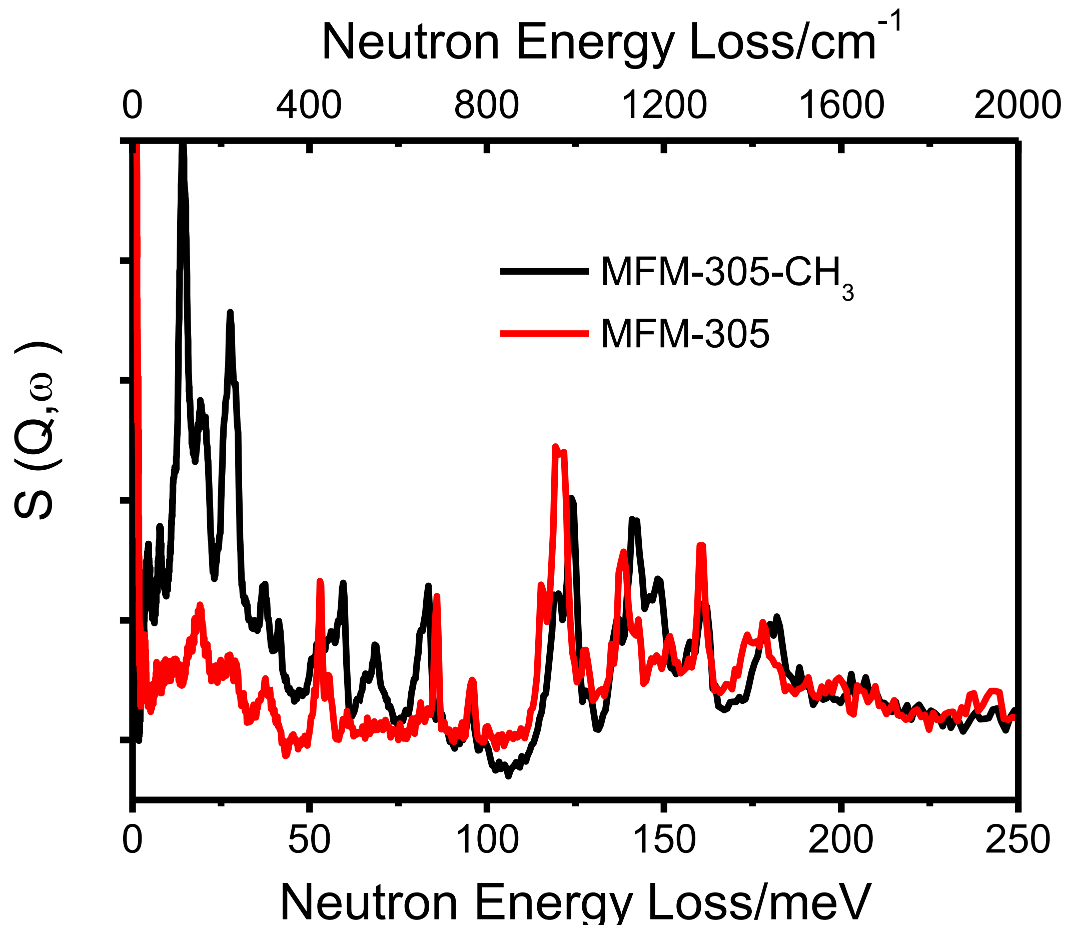

Figure S35. Comparison of the experimental INS spectra for MFM-305-CH_3 and MFM-305.

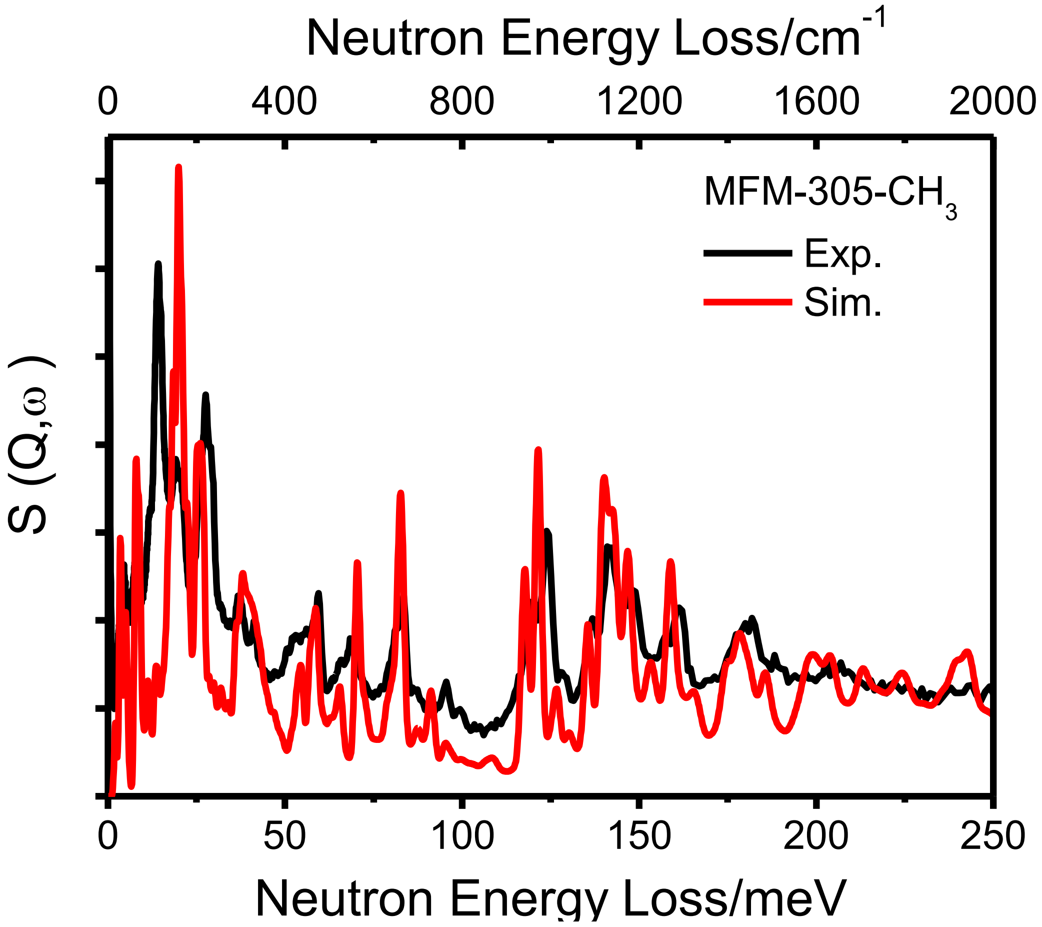

Figure S36. Comparison of the experimental and simulated INS spectra for MFM-305-CH_3.

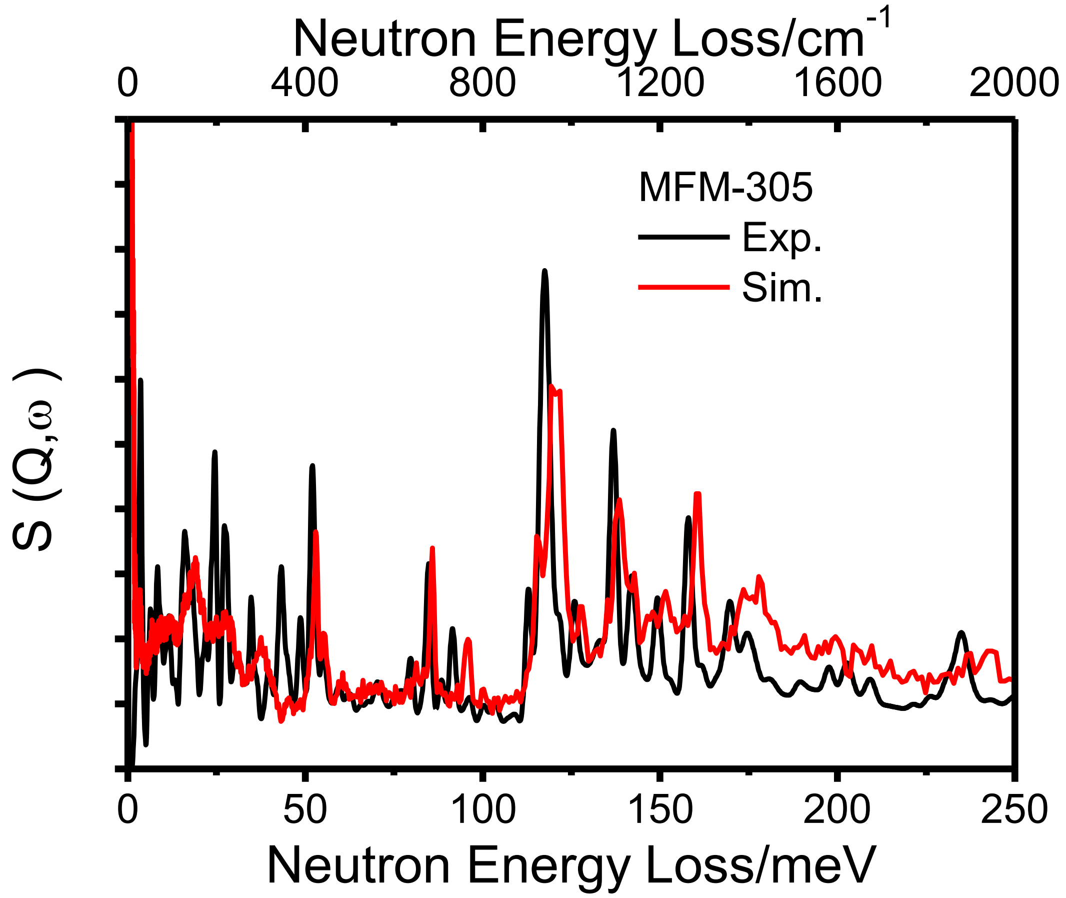

Figure S37. Comparison of the experimental and simulated INS spectra for MFM-305.

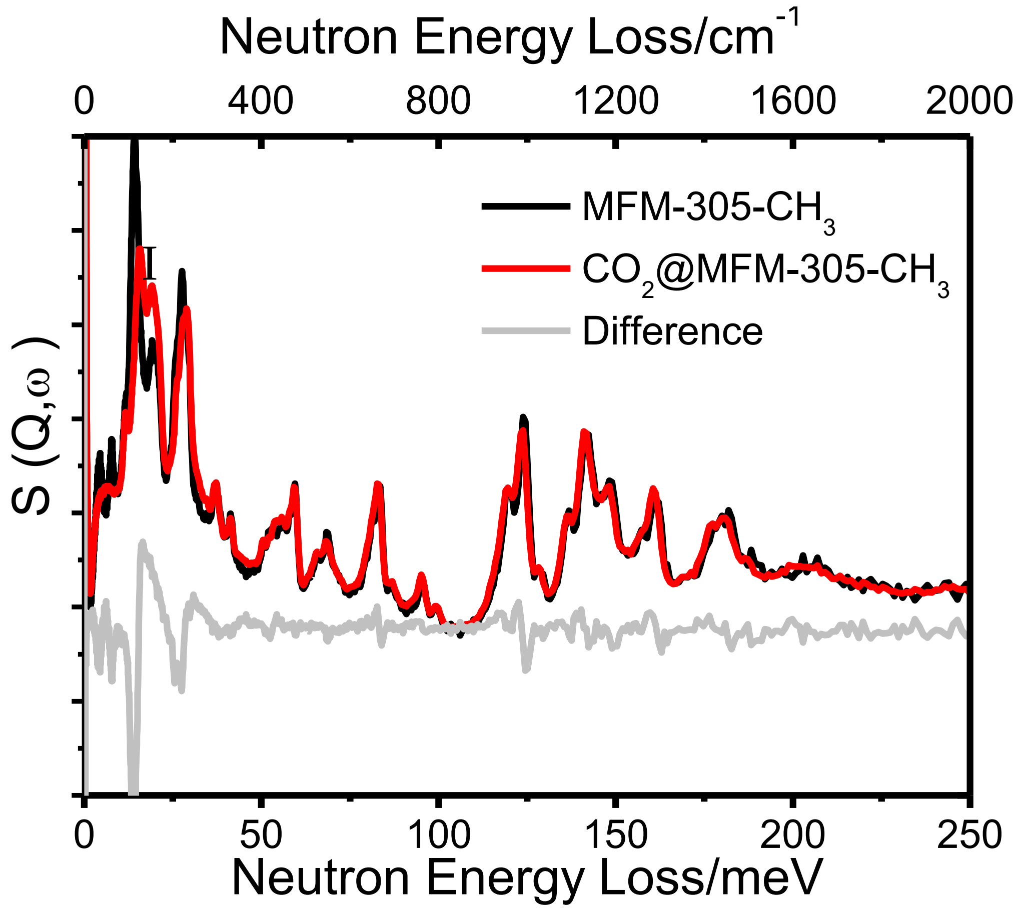

Figure S38. Comparison of the experimental INS spectra for bare and CO_2-loaded MFM-305-CH_3.

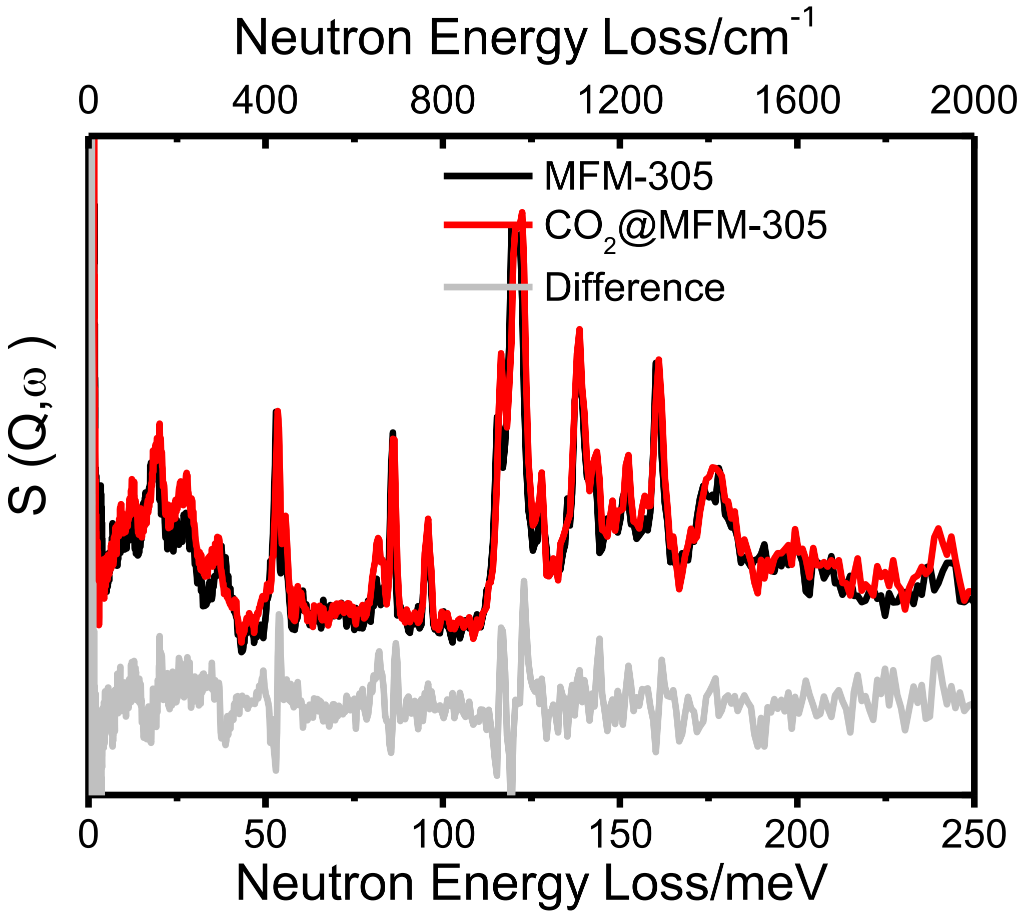

Figure S39. Comparison of the experimental INS spectra for bare and CO_2-loaded MFM-305.

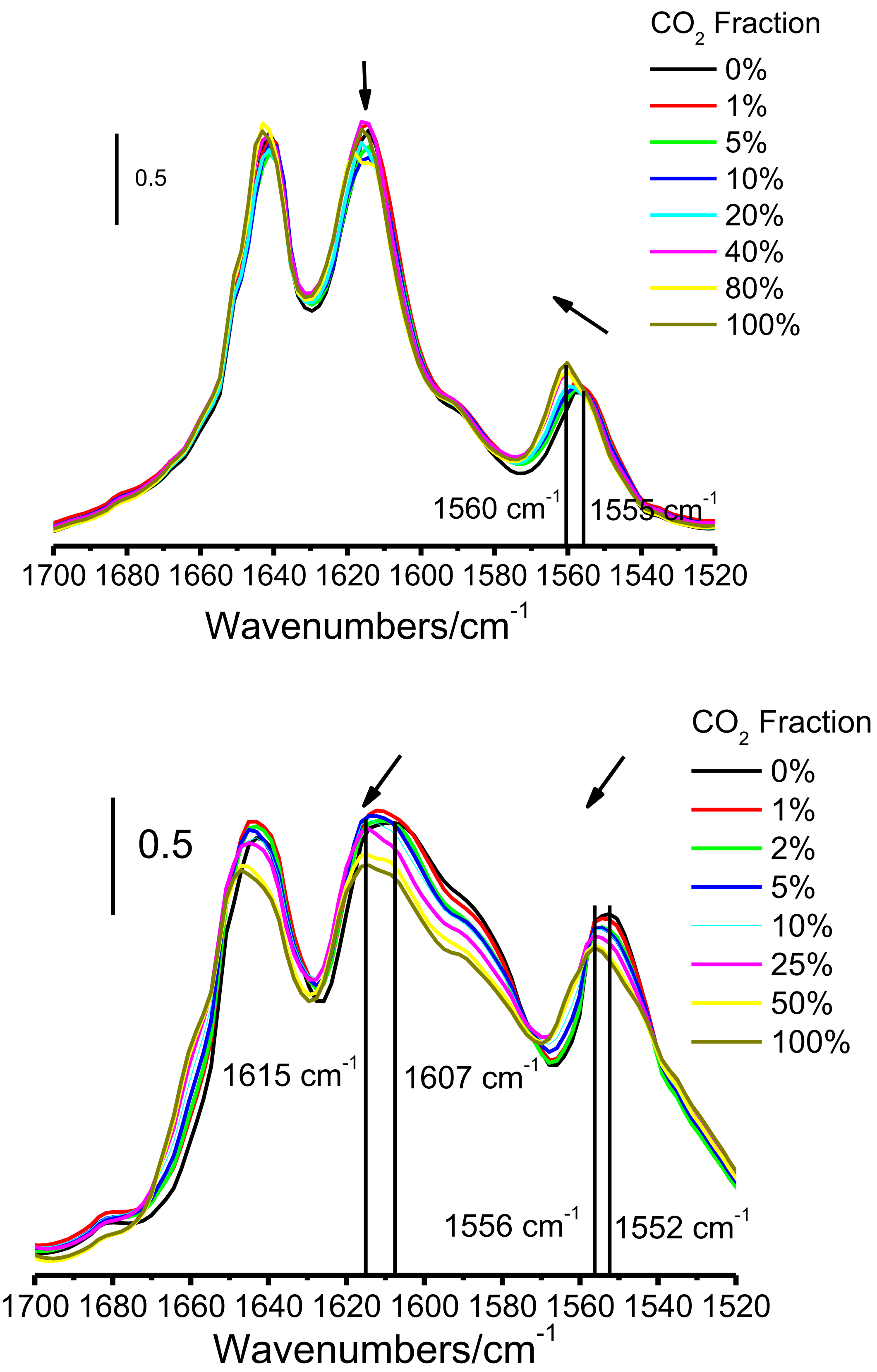

Figure S40. DRIFTS spectra of CO_2-loaded MFM-305-CH_3 (top) and MFM-305 (bottom) recorded from 0% to 100% (1bar).

Table S3. Summary of Crystal Structure Data

**MFM-305-CH_3**		273K	230K	198K	150K	117K	7K	SO_2
Formula	$Al(OH)(C_8H_6NO_4)Cl$	$Al(OH)(C_8H_6NO_4)Cl\cdot 0.60CO_2$	$Al(OH)(C_8H_6NO_4)Cl\cdot 0.70CO_2$	$Al(OH)(C_8H_6NO_4)Cl\cdot 0.72CO_2$	$Al(OH)(C_8H_6NO_4)Cl\cdot 0.71CO_2$	$Al(OH)(C_8H_6NO_4)Cl\cdot 0.67CO_2$	$Al(OH)(C_8H_6NO_4)Cl\cdot 0.71CO_2$	$Al(OH)(C_8H_6NO_4)Cl\cdot 0.77SO_2$
Crystal system	Tetragonal	Tetragonal	Tetragonal	Tetragonal	Tetragonal	Tetragonal	Tetragonal	Tetragonal
Space group	$I4_1/amd$	$I4_1/amd$	$I4_1/amd$	$I4_1/amd$	$I4_1/amd$	$I4_1/amd$	$I4_1/amd$	$I4_1/amd$
a, b/ Å	21.48(6)	21.4749(1)	21.4715(1)	21.4703(1)	21.4630(1)	21.4577(1)	21.48(6)	21.4987(1)
c / Å	10.90(3)	10.9000(1)	10.8861(1)	10.8751(1)	10.8553(1)	10.8407(1)	10.88(3)	10.8528(1)
V/ Å^3	5030(30)	5026.8(1)	5018.8(1)	5013.1(1)	5000.6 (1)	4991.4(1)	5020(40)	5016.1(1)
Z	8	8	8	8	8	8	8	8
Sample size (mm)	Cylinder, 20 × 0.7						Cylinder, 65 x 8	Cylinder, 20 × 0.7
Radiation type	Synchrotron X-Ray λ = 0.825250						Neutron	Synchrotron X-Ray λ = 0.825250
Scan method	Continuous scan						Time of Flight	Continuous scan
R_{exp}	3.18	3.74	3.74	3.72	3.68	3.64	0.19	1.94
Rwp/%	6.09	6.07	6.27	6.29	6.26	6.14	1.13	5.64
Rp/%	4.76	3.74	3.74	3.72	4.91	4.77	0.94	4.36
GOOF	1.91	1.62	1.68	1.69	1.70	1.68	5.96	1.40
CCDC	1580831	1580823	1580822	1580821	1580820	1580819	1580818	1580833
**MFM-305**		270K	230K	198K	150K	100K		SO_2
Formula	$Al(OH)(C_7H_3NO_4)$	$Al(OH)(C_7H_3NO_4)\cdot 0.50CO_2$	$Al(OH)(C_7H_3NO_4)\cdot 0.75CO_2$	$Al(OH)(C_7H_3NO_4)\cdot 0.92CO_2$	$Al(OH)(C_7H_3NO_4)\cdot 0.92CO_2$	$Al(OH)(C_7H_3NO_4)\cdot 0.88CO_2$		$Al(OH)(C_7H_3NO_4)\cdot 0.75SO_2$
Crystal system	Tetragonal	Tetragonal	Tetragonal	Tetragonal	Tetragonal	Tetragonal		Tetragonal
Space group	$I4_1/amd$	$I4_1/amd$	$I4_1/amd$	$I4_1/amd$	$I4_1/amd$	$I4_1/amd$		$I4_1/amd$
a, b / Å	21.4952(3)	21.4813(2)	21.4612(2)	21.4541(2)	21.4452(2)	21.4765(2)		21.4658(2)
c / Å	10.4572(2)	10.6140(2)	10.7306(2)	10.7853(2)	10.7670 (1)	10.7477(2)		10.7571(2)
V / Å^3	4831.6(1)	4897.8(1)	4942.3(1)	4964.2(1)	4951.7(1)	4957.3(1)		4956.7(1)
Z	8	8	8	8	8	8		8
Sample size (mm)	Cylinder, 20 × 0.7							Cylinder, 20 × 0.7
Radiation type	Synchrotron X-Ray λ = 0.825250							Synchrotron X-Ray λ = 0.825250
Scan method	Continuous scan							Continuous scan
R_{exp}	3.68	3.41	3.42	3.41	3.40	3.39		3.38
Rwp	5.95	5.75	6.15	5.92	5.62	6.04		5.95
Rp	4.63	4.39	4.64	4.50	4.30	4.61		4.53
GOOF	1.61	1.69	1.80	1.74	1.78	1.78		1.76
CCDC	1580832	1580829	1580828	1580827	1580826	1580825		1580834

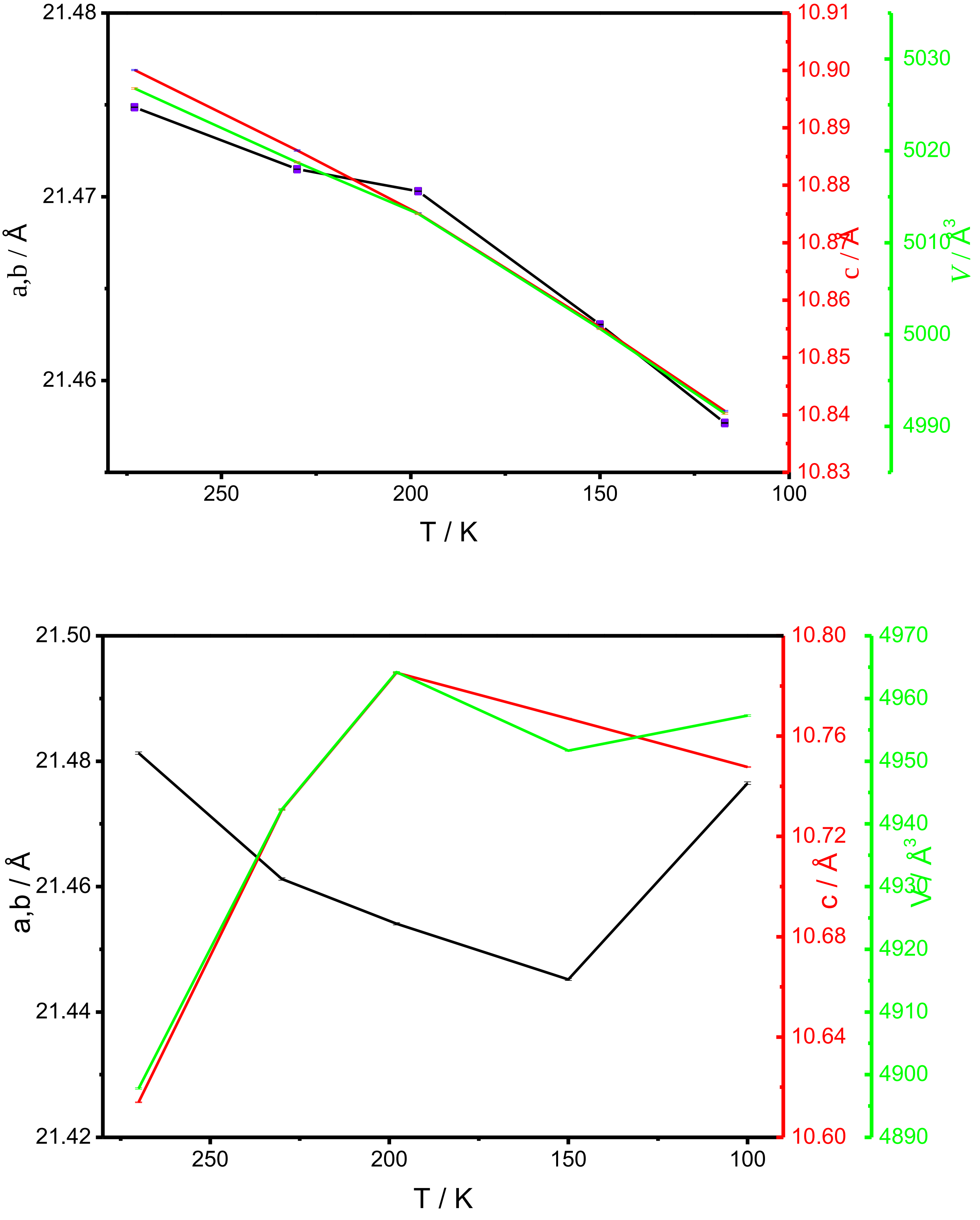

Figure S41. Cell parameters for CO_2-loaded MFM-305-CH_3 (top) and MFM-305 (bottom) at different temperature.

Table S4. Bond lengths of MFM-305-CH_3 and MFM-305.

	CO_2							SO_2
MFM-305-CH_3	273 K	230 K	198 K	150 K	117 K	7 K	MFM-305-CH_3	298 K
OC$\underline{O}^{I}$-$H_3\underline{C}$/ Å	3.10(2)	2.51(1)	2.54(1)	2.47(2)	2.38(2)	3.58(2)	OS$\underline{O}^{I}$-$H_3\underline{C}$/ Å	2.49(3)
OC$\underline{O}^{I}$-Cl/ Å	4.63(1)	3.83(1)	3.78(1)	3.74(1)	3.77(1)		OS$\underline{O}^{II}$-$H_3\underline{C}$/ Å	3.73(2)
OC$\underline{O}^{I}$-$\underline{H2}$C/ Å	3.00(2)	2.89(1)	2.87(2)	2.83(2)	2.78(2)	3.08(2)	OS$\underline{O}^{I}$-$\underline{H2}$C/ Å	2.54(2)
OC$\underline{O}^{II}$-$\underline{H1}$C/ Å	4.02(2)	3.44(2)	3.50(1)	3.44(1)	3.44(1)		OS$\underline{O}^{I}$ - O$\underline{S}$O^{II} /Å	3.97(3)
OC$\underline{O}^{I}$ - O$\underline{C}$O^{II} /Å	3.74(2)	3.81(2)	3.87(1)	3.86(2)	3.91(2)		O$\underline{S}$O^{I} - $\underline{O}$SO^{II} /Å	3.38(2)
O$\underline{C}$O^{I} - $\underline{O}$CO^{II} /Å	3.81(2)	4.06(1)	4.10(1)	4.14(1)	4.09(2)			
MFM-305	270 K	230 K	198 K	150 K	100 K		MFM-305	298 K
OC$\underline{O}^{I}$-$\underline{H}$O/ Å	4.20(3)	3.66(4)	3.45(3)	3.44(3)	3.34(4)		OS$\underline{O}$-$\underline{H}$O/Å	3.42(4)
OC$\underline{O}^{I}$-$\underline{H2}$C/ Å	3.18(1)	2.88(2)	2.73(1)	2.67(1)	2.72(2)		N-O$\underline{S}$O^{II}/ Å	2.78(1)
	2.67(2)	2.42(2)	2.44(1)	2.48(1)	2.38(2)		OS$\underline{O}^{I}$-$\underline{H2}$C/ Å	2.63(3)
N-O$\underline{C}$O^{I}/ Å	3.16(1)	2.97(1)	2.89(1)	2.86(0)	2.96(1)		OS$\underline{O}^{II}$-$\underline{H2}$C/ Å	3.14(3)
OC$\underline{O}^{I}$ - O$\underline{C}$O^{II} /Å	5.03(2)	3.06(2)	3.14(1)	3.11(1)	3.07(1)		O$\underline{S}$O^{I} - $\underline{O}$SO^{II} /Å	4.29(2)
O$\underline{C}$O^{I} - $\underline{O}$CO^{II} /Å	5.04(3)	2.99(2)	3.09(1)	3.09(1)	2.94(1)			

References

1 K. M. Broadus and S. R. Kass, *J. Am. Chem. Soc.*, 2000, **122**, 9014-9018.

2 G. Kresse and J. Furthmüller, *Phys. Rev. B*, 1996, **54**, 11169-11186.

3 P E. Blöchl, *Phys. Rev. B*, 1994, **50**, 1795317979.

4 G. Kresse and D. Joubert. *Phys. Rev. B*, 1999, **59**, 1758-1775.

5 J. P. Perdew, K, Burke and M. Ernzerhof, *Phys. Rev. Lett.*, 1996, **77**, 3865-3868.

6 J. Klimeš, D. R. Bowler and A. Michaelides, *J. Phys. Cond. Mat.*, 2010, **22**, 022201.

7 A. Togo and I. Tanaka, *Scripta Mat.*, 2015, **108**, 1-5.

8 A. J. Ramirez-Cuesta, *Comput. Phys. Commun.*, 2004, **157**, 226238.

9 C. A. Fernandez, P. K. Thallapally, R. K. Motkuri, S. K. Nune, S J. C. umrak, J. Tian and J. Liu, *Cryst. Growth Des.*, 2010, **10**, 1037-1039.

10 X. L. Cui, Q. W. Yang, L. F. Yang, R. Krishna, Z. G. Zhang, Z. B. Bao, H. Wu, Q. L. Ren, W. Zhou, B. L. Chen and H. B. Xing, *Adv. Mat.*, 2017, **29**, 1606929.

11 D. Britt, D. Tranchemontagne and O. M. Yaghi, *Proc. Natl. Acad. Sci. USA*, 2008, **105**, 11623-11627.

12 P. K.Thallapally, R. K. Motkuri, C. A. Fernandez, B. P. McGrail and G. S. Behrooz, *Inorg. Chem.*, 2010, **49**, 49094915.

13 S. H. Yang, J. L. Sun, A. J. Ramirez-Cuesta, S. K. Callear, W. I. F. David, D. P. Anderson, R. Newby, A. J. Blake, J. E. Parker, C. C. Tang and M. Schröder, *Nat. Chem.*, 2012, **4**, 887894.

14 M. Savage, Y. G. Cheng, T. L. Easun, J. E. Eyley, S. P. Argent, M. R. Warren, W. Lewis, C. Murray, C. C. Tang, M. D. Frogley, G. Cinque, J. L. Sun, S. Rudic, R. T. Murder, M. J. Benham, A. N. Fitch, A. J. Blake, A. J. Ramirez-Cuesta, S. H. Yang and M. Schröder, *Adv. Mat.*, 2016, **28**, 8705-8711.

15 K. Tan, P. Canepa, Q. H. Gong, J. Liu, D. H. Johnson, A. Dyevoich, P. K. Thallapally, T. Thonhauser, J. Li and Y. J. Chabal, *Chem. Mat.*, 2013, **25**, 4653-4662.

16 S. H. Yang, L. F. Liu, J. L. Sun, K. M. Thomas, A. J. Davies, M. W. George, A. J. Blake, A. H. Hill, A. N. Fitch, C. C. Tang and M. Schröder, *J. Am. Chem. Soc.*, 2013, **135**, 4954-4957.
